# Stability of cytokine and immunoglobulin concentrations in the general population: prepandemic basal concentrations and intraindividual changes until the COVID-19 pandemic

**DOI:** 10.3389/fpubh.2025.1548379

**Published:** 2025-07-02

**Authors:** Magda Gasull, José Pumarega, Ruth Aguilar, Laura Campi, David Prieto-Merino, Judit Villar-García, Cristina Rius, Francisco Bolúmar, Leonardo Trasande, Carlota Dobaño, Gemma Moncunill, Miquel Porta

**Affiliations:** ^1^Hospital del Mar Research Institute, Barcelona, Spain; ^2^Universitat Pompeu Fabra, Barcelona, Spain; ^3^CIBER de Epidemiología y Salud Pública (CIBERESP), Barcelona, Spain; ^4^School of Medicine, Universitat Autònoma de Barcelona, Barcelona, Spain; ^5^ISGlobal, Barcelona, Spain; ^6^Facultat de Medicina i Ciències de la Salut, Universitat de Barcelona (UB), Barcelona, Spain; ^7^University of Alcalá de Henares, Madrid, Spain; ^8^Agència de Salut Pública de Barcelona, Barcelona, Spain; ^9^City University of New York, New York, NY, United States; ^10^Division of Environmental Pediatrics, Department of Pediatrics, School of Medicine, New York University, New York, NY, United States; ^11^Department of Population Health, New York University, New York, NY, United States; ^12^Wagner School of Public Service, New York University, New York, NY, United States; ^13^CIBER de Enfermedades Infecciosas (CIBERINFEC), Barcelona, Spain; ^14^Department of Epidemiology, Gillings School of Global Public Health, University of North Carolina, Chapel Hill, NC, United States

**Keywords:** cytokines, immunoglobulins, SARS-CoV-2, COVID-19, population-based

## Abstract

**Background:**

While there is wide evidence on concentrations of cytokines in patients attending health care facilities, evidence is scant on physiological, basal concentrations of cytokines in the general population and across sociodemographic groups, as well as on their potential stability over time. Furthermore, from a public health perspective it is remarkable that no studies have analyzed intraindividual changes in such concentrations from before the COVID-19 pandemic until its outbreak.

**Objectives:**

To investigate: (a) prepandemic concentrations of cytokines and immunoglobulins to viral exposures in a general, non-institutionalized population, and their associated sociodemographic variables; (b) the intraindividual change in such concentrations between a prepandemic period (2016–17) and the initial pandemic period (2020–21); and (c) whether such change was similar in participants who in 2020–21 were SARS-CoV-2 seronegative and seropositive, and between participants who did and did not develop COVID-19.

**Methods:**

We conducted a prospective cohort study in 240 individuals from the general population of Barcelona, Spain. Thirty cytokines and 31 immunoglobulins were measured in paired serum samples collected in 2016–17 and 2020–21 in the same individuals.

**Results:**

The median value of the relative intraindividual change in cytokine concentrations between 2016 and 2020 was <15% for 29 of the 30 cytokines. A substantial number of participants had an intraindividual increase or decrease ≥15% in some cytokines. No major differences in intraindividual changes of cytokine and immunoglobulin levels between 2016 and 2020 were observed between participants who did and did not develop COVID-19.

**Conclusion:**

We provide novel information on physiological, basal ex-vivo concentrations of cytokines and immunoglobulins in a general population, which should be relevant for clinical practice and public health. Intraindividual changes in cytokines and immunoglobulins during the 4 years from 2016–17 to 2020–21 were moderate, and they did not differ between participants who in 2020–21 were SARS-CoV-2 seropositive and seronegative, nor between participants who did and did not develop COVID-19 disease. These findings are also novel and relevant for medicine and public health. In particular, the stability in the biomarkers is relevant to assess the role of the immunological and inflammatory state (measured through baseline levels of cytokines and immunoglobulins) in the development of SARS-CoV-2 seropositivity and COVID-19 disease, as well as in the susceptibility to other infections and pathologies.

## 1 Introduction

While there is wide evidence on concentrations of cytokines in patients attending health care facilities, evidence is scant on physiological, basal ex-vivo concentrations of cytokines in the general population and across sociodemographic groups, as well as on their potential stability over time. Variation within individuals of cytokine concentrations is not well characterized either (1–11). Furthermore, from a public health perspective it is remarkable that no studies have analyzed intraindividual changes in concentrations of cytokines and immunoglobulins from the period before the COVID-19 pandemic until its outbreak (12, 13).

It is certainly well known that individual levels of cytokines fluctuate considerably during the clinical course of many diseases (1–4, 9, 11), as was –and is– also the case in patients with COVID-19 (14–21). While concentrations of cytokines and immunoglobulins return to baseline levels at convalescence or recovery, others seem to persist altered for longer periods of time, reflecting more persistent alterations of the cellular immune system (22–27). Assessing the influence of basal physiological ex-vivo levels of cytokines and immunoglobulins on the risk of SARS-CoV-2 infection and COVID-19 disease (28) requires that such biomarkers be measured before the pandemic, and that they remain relatively stable over time. Additional biomarkers of interest related to baseline immune state, which could also predict infection and disease susceptibility are total antibody isotypes and subclasses, and antibodies against chronic viruses such as cytomegalovirus (CMV) and Epstein–Barr virus (EBV), known to affect the immune system (3, 4, 29). EBV infects B cells and alters the development of regulatory NKT subsets (30). CMV activates many arms of the immune system, and together with its modulatory strategies results in a major impact on immune system homeostasis (31). Other viral exposures may shape as well the immune system and influence responses to further challenges; particularly, pre-existing immunity to common cold human coronaviruses (HCoV) may affect the risk of SARS-CoV-2 infection and COVID-19 susceptibility through antibody crossreactivity (12, 13). It has been reported that pre-existing humoral and cellular immunity to HCoV impacts the outcomes of SARS-CoV-2 infection and COVID-19 disease (32–34).

Assessing the variation over time of cytokine and immunoglobulin concentrations, and the influence on such levels of sociodemographic and lifestyle factors in a general Western population can provide novel information with potential uses in clinical practice and research on biomarkers of disease susceptibility. The present study stands out for its unique value of having paired pre-pandemic and pandemic immune profiles from the same individuals. Thus, the longitudinal approach advances current understanding compared to previous cross-sectional or disease-specific studies.

The objectives of the present study were to investigate (a) prepandemic concentrations of cytokines, total immunoglobulins and immunoglobulins to viral exposures in a general, non-institutionalized population, and their associated sociodemographic variables; (b) the intraindividual change in such concentrations between a prepandemic period (2016–17) and the initial pandemic period (2020–21); and (c) whether such change was similar or different in participants who in 2020–21 were SARS-CoV-2 seronegative and seropositive, and between participants who did and did not develop COVID-19.

## 2 Methods

### 2.1 Study population

The present prospective cohort study was based on the Barcelona Health Survey (BHS) of 2016, whose methods have been described in detail (35–37). The BHS generated a sample representative of the general, adult, non-institutionalized population of the city of Barcelona (Spain). Through face-to-face interviews, the survey collected information about sociodemographic factors, chronic disorders, life styles, uses of healthcare services and preventive practices. At the end of the 2016 BHS interview, participants were offered to take part in a health examination, and 240 individuals accepted. Subsequently, between 15 July 2016 and 4 May 2017, a nurse interviewed again face-to-face such individuals, measured body parameters, and collected blood and urine samples (35, 37). Participants had been asked to fast for at least 8 h before blood extraction. Blood was collected in a vacuum system tube and centrifuged for 15 min × 3,000 rpm at 4°C to obtain serum, which was divided in 1–3 mL aliquots and stored at −80°C (35, 37). The prepandemic levels of the cytokines and immunoglobulins assessed in the present report were analyzed in such serum samples (see sections 2.3., 2.4., and 2.5. below).

After scientific, financial and logistic preparations, on October 2020, in a severe phase of the pandemic, the 240 participants began to be invited to a follow-up visit, which 174 (72.5%) attended between 18 November 2020 and 7 June 2021 (35). Thus, for the present analyses our study spans from 2016 to 17, when the baseline interviews and collection of biological samples first took place, to 2020–21, when the follow-up visit and collection of biological samples took place again. During the follow-up visit a nurse measured their weight, height. She also collected a nasopharyngeal swap, and new blood and urine samples, which constitute a crucial scientific resource of the present cohort study to analyze immunological components of the SARS-CoV-2 infection. The median time between the extraction of biological samples in 2016–17 and 2020–21 was 4.1 years. Compared to the 66 subjects who did not attend the follow-up visit, the 174 participants were more commonly women, younger, born in Catalonia, with a lower body mass index (BMI), more affluent, and with better self-perceived health (35). While some analyses reported in the present paper are based on the 174 individuals, analyses of the intraindividual change (from 2016–17 to 2020–21) of cytokines and immunoglobulins are based on the 154 participants who had not received any COVID-19 vaccine at the time of the follow-up visit (35). Characteristics of the 154 participants have been published in Table 1 of Porta et al. (35).

The Ethics Committee of the Parc de Salut Mar reviewed and approved the study protocols, and all participants signed an informed consent before sample collection and completing questionnaires (37). All methods were performed in accordance with the relevant guidelines and regulations.

### 2.2 Socioeconomic and living conditions

Shortly before the follow-up visit in 2020–21, the participants completed an online survey concerning signs and symptoms of COVID-19, diagnostic tests performed and their results, use of healthcare services, and vaccination, all during the previous months of the pandemic. This information was ascertained as well with the data bases of the System of Diseases of Mandatory Reporting of the Agency of Public Health of Barcelona, and of the Public Data Analysis for Health Research and Innovation Program of Catalonia (PADRIS) of the Healthcare Quality and Evaluation Agency of Catalonia (AQUAS) (38). The PADRIS databases contain detailed records on demographics, laboratory results, medications dispensed by pharmacies, Primary Care physician visits, procedures, and medical admissions from public hospitals across Catalonia; therefore, PADRIS allows the retrieval of diagnoses of all diseases and health disorders and conditions recorded in primary care and public hospitals, including chronic diseases such as hypertension, dyslipidemia, osteoarthritis, among others (see section 2.7). This data was used to complement information collected during the study. During follow-up the study also collected information on participants’ lifestyle and living conditions during the pandemic (35). During the visit, the nurse clarified answers to the online survey and asked further questions on vaccination, weight changes, and pregnancies. A household outdoor index was computed taking into account the number of individuals living in the same household, the availability and use of an outdoor space; the score of the index increased as the number of individuals increased and the availability and frequency of use of the outdoor space decreased. Other factors included in the online survey were: work conditions, use of public and private transport, and individual measures taken to avoid infection (35).

### 2.3 Quantification of cytokines, chemokines and growth factors

**Figure 1:**
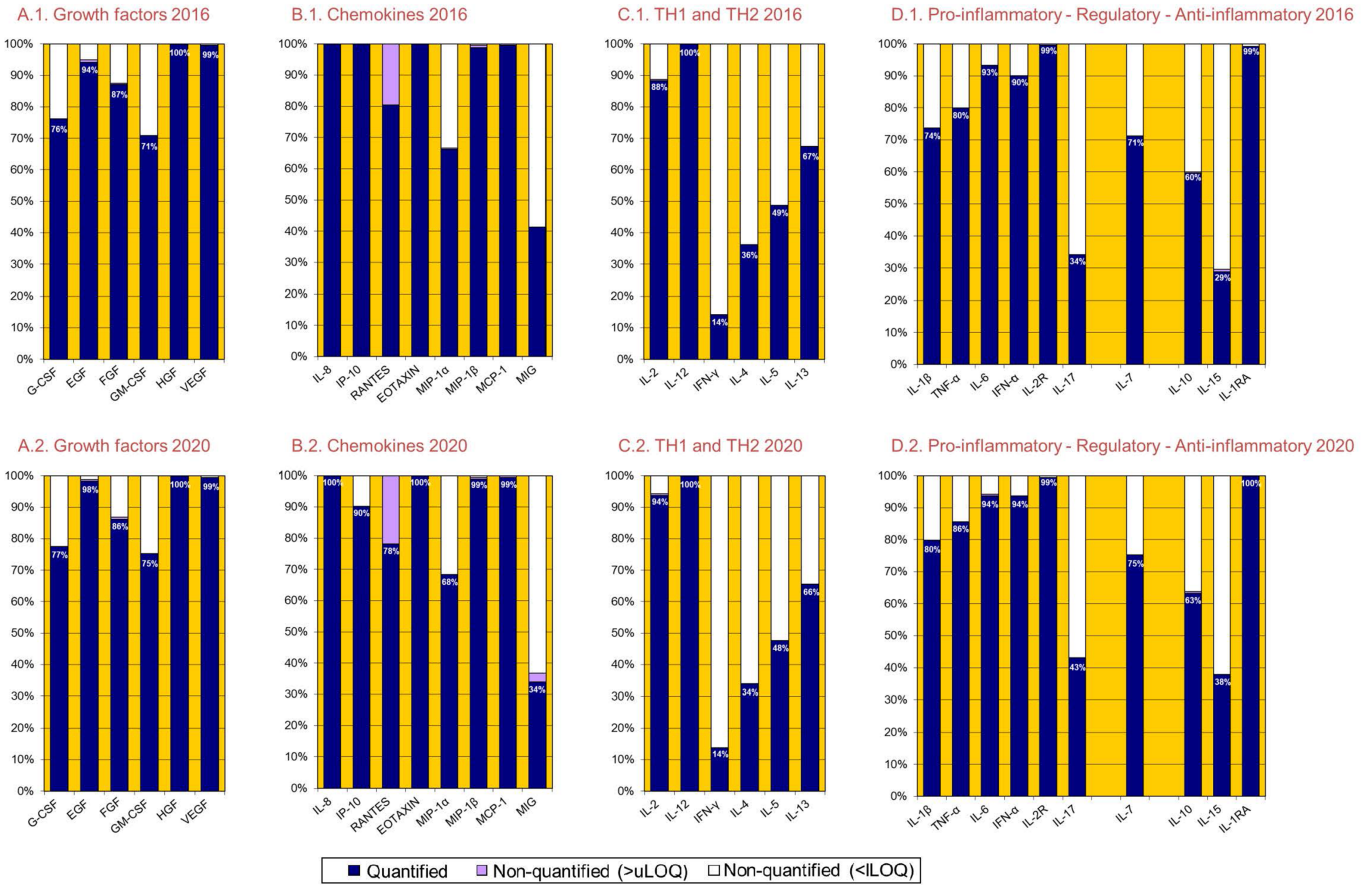
Percentage of quantification of 30 cytokines in 2016–17 and 2020–21.

The Cytokine Human Magnetic 30-Plex Panel from Invitrogen™ was used to measure concentrations (pg/mL) of the following 30 cytokines, chemokines and growth factors in the prepandemic (2016–17) and pandemic (2020–21) serum samples: (39, 40) epidermal growth factor (EGF), fibroblast growth factor (FGF), granulocyte colony-stimulating factor (G-CSF), granulocyte-macrophage colony-stimulating factor (GM-CSF), hepatocyte growth factor (HGF), vascular endothelial growth factor (VEGF), tumor necrosis factor (TNF), interferon (IFN)-*α*, IFN-*γ*, interleukin (IL)-1RA, IL-1β, IL-2, IL-2R, IL-4, IL-5, IL-6, IL-7, IL-8, IL-10, IL-12(p40/p70), IL-13, IL-15, IL-17, IFN-γ induced protein (IP-10), monocyte chemoattractant protein (MCP-1), monokine induced by IFN-γ (MIG), macrophage inflammatory protein (MIP)-1α, MIP-1β, regulated on activation normal T cell expressed and secreted (RANTES) and eotaxin. Individually paired prepandemic and pandemic samples were tested in the same assay plate. Each plate included 16 serial dilutions (2-fold) of a standard curve, and two blank controls. Samples were acquired on a Luminex 100/200 instrument and analyzed in xPONENT software 3.1. The concentration of each analyte was obtained by interpolating the median fluorescent intensity (MFI) to a 5-parameter logistic regression curve and reported as pg./mL using the drLumi R package. Methods to measure cytokines, chemokines and growth factors concentrations were based on a previous study in which the performance of several commercial kits was compared; we chose the method with the most accurate results. In addition, the technique was improved by making some changes to the experimental design of the controls and the standard curve (41). Limits of quantification (LOQ) were estimated based on cutoff values of the 30% coefficient of variation (CV) of the standard curve for each analyte. When the value of an analyte was below the lower LOQ, the mid-value of this limit for the corresponding plate was assigned; and when a sample value was above the corresponding upper LOQ, the assigned value was twice this LOQ. Lower LOQ ranged from 0.07 pg./mL for VEGF to 16.2 pg./mL for G-CSF; upper LOQ ranged from 981 pg./mL for IP-10 to 77,553 pg./mL for IL-1RA (Supplementary Table 1). For most cytokines, the percentage of quantification (i.e., the percentage of participants with concentrations between the lower and the upper LOQ) was > 70%, while for 9 cytokines the percentage of quantification ranged from 14% for IFN-*γ* to 68% for MIP-1α (Supplementary Table 1; Figure 1).

The intraindividual changes in the concentrations reported in this paper were not due to changes in the percentages of quantification of each cytokine or due to changes in the limits of quantification: the two samples of each individual (2016–17 and 2020–21) were analyzed in the same laboratory plate (thus, at the same time) with the same lower and upper limit of quantification (Supplementary Table 1; Figure 1).

### 2.4 Serology of viral exposures

The levels of IgM, IgA and IgG against the Nucleocapsid protein of the 4 human common cold coronaviruses (HCoV-229E, OC43, NL63, HKU1), two Epstein–Barr virus (EBV) antigens (EA-D, VCA p18), and two Cytomegalovirus (CMV) antigens (pp65, pp150), were assessed by high-throughput multiplex quantitative suspension array technology (qSAT) in a FlexMap3D instrument, as previously described (12, 13), and data QA/QC and preprocessing were performed with R. Briefly, antigen-coupled beads were added to a 384-well μClear® flat bottom plate in multiplex. A hyper-immune plasma pool at 3-fold 10 serial dilutions starting from 1:250 was used as positive control in each assay plate for QA/QC and calibration purposes. Final dilution of test samples was 1:500. To quantify IgA and IgM, samples and controls were pre-treated with anti-human IgG (Gullsorb) at 1:10 dilution, to avoid IgG interferences. Median fluorescence intensity (MFI) was reported for each isotype-antigen.

### 2.5 Quantification of total immunoglobulins

The quantification of total immunoglobulins (IgE, IgA, IgM, IgG1, IgG2, IgG3, IgG4) was performed with the Antibody Isotyping 7-Plex Human ProcartaPlex™ panel (Thermo Fisher Scientific, Vienna, Austria) following the manufacturer’s instructions. Samples were tested at 1/200000 and 1/500000 dilutions, acquired on a Luminex 100/200 instrument and analyzed in xPONENT software 3.1. The concentration of each isotype was obtained by interpolating the median fluorescent intensity (MFI) to a 5-parameter logistic regression curve and reported as μg/mL.

In the 240 prepandemic samples, no significant correlations among cytokines and IgA and IgG isotypes against HCoV, CMV and EBV were observed. By contrast, 12 cytokines showed positive, statistically significant correlations with all 8 IgMs against HCoV, CMV and EBV, whereas two chemokines showed inverse correlations (all *ρ* ≤ 0.35; Supplementary Figure 1; Supplementary Table 2).

Some significant correlations were observed among pairs of cytokines; the highest correlation coefficients were observed between IL-2 and IFN-*α*, IL-2 and MIP-1β, and IFN-α and MIP-1β (all three *ρ* > 0.84 and *p* < 0.001; Supplementary Figure 2). A correlation coefficient of 0.73 between G-CSF and TNF-α was also observed (*p* < 0.001).

While most correlation coefficients among the 24 isotype-antigen combinations and the 8 total immunoglobulins were <0.43, some high coefficients were observed between total IgM and the IgMs against CMV, EBV and HCoV; e.g., among total IgM and IgM EBV EAD, IgM N HKU1, and IgM CMV pp65 (all three ρ > 0.66 and *p* < 0.001; Supplementary Figure 3).

### 2.6 Determination of SARS-CoV-2 infection and COVID-19 disease

#### 2.6.1 SARS-CoV-2 infection

SARS-CoV-2 infection was determined at the Center for Genomic Regulation (CRG) in all 174 members of the cohort who attended the follow-up visit in 2020–21 by real time reverse-transcriptase polymerase chain reaction (rRT-PCR) in nasopharyngeal swabs. Briefly, samples were collected in 600 μL of lysis solution (DNA/RNA Shield, Zymo) to inactivate the virus, break membranes and stabilize the RNA. Samples were processed in a TECAN Dreamprep robot to isolate the RNA using the Quick-DNA/RNA Viral MagBead kit (Zymo; #R2140), and the purified RNA was analyzed by rRT-PCR in an ABI 7900 HT (384 wells) following the CDC standard procedure. Positive and negative controls were included in each assay plate. Among the 174 participants, there were 4 rRT-PCR-positives (35).

To detect previous SARS-CoV-2 infections, antibody serological status of each participant was assessed in serum samples analyzed at the ISGlobal Immunology Laboratory in Barcelona. The MFI levels of IgG, IgM and IgA were assessed by high-throughput multiplex quantitative suspension array technology, including 5 SARS-CoV-2 antigens (35), as described in section 2.4 for the other viral exposures (15, 42).

Of the 154 participants mentioned above, 41 were SARS-CoV-2 seropositive (26.6%) at the time of the follow-up visit in 2020–21 (including all 4 positives by the follow-up rRT-PCR), 9 indeterminate (5.8%), and 104 seronegative (67.5%). There were no major differences in the main characteristics of seropositive and seronegative participants (Supplementary Table 5 of 35).

#### 2.6.2 COVID-19 disease

Cases of COVID-19 disease have been described in detail (35). In total there were 20 cases of COVID-19 disease at the time of the follow-up visit in 2020–21. All were seropositive for SARS-CoV-2 in our immunological assay, and all reported COVID-19 related symptoms. Specifically, 10 cases provided information of a positive diagnostic test for SARS-CoV-2 infection (including all 4 positives at the follow-up rRT-PCR), and 2 or more COVID-19 related signs or symptoms; 2 were diagnosed of COVID-19 by a physician; and 8 had COVID-19 related signs or symptoms (35, 43). There were no major differences in the main characteristics of participants with and without COVID-19 (Table 1 of 35).

### 2.7 Comorbidities

Detailed information on comorbidities was obtained from PADRIS (section 2.2) and the BHS (section 2.1). Specifically, comorbidities were identified based on diagnostic codes from PADRIS, using the International Classification of Diseases, 10th Revision (ICD-10). All available diagnoses recorded prior to the COVID-19 pandemic were reviewed and classified into major disease categories such as cardiovascular, respiratory, and musculoskeletal disorders, among others. This strategy aligns with previous research that has employed PADRIS data to assess chronic disease prevalence and multimorbidity patterns in the Catalan population (44–46).

No associations among prepandemic comorbidities, and cytokines or immunoglobulins, were observed: most correlation coefficients among comorbidities and the 30 cytokines, the 24 isotype-antigen combinations and the 8 total immunoglobulins were <0.25, and not statistically significant (Supplementary Figures 4a,b). Only some modest negative coefficients were observed between IgMs and BMI, and between IgMs, some total IgGs and dyslipidemia (Supplementary Figure 4b): the highest correlations were for IgMs against CMV pp65 and EBV VCA p18 with BMI (*ρ* = −0.324 and −0.280, respectively, *p* < 0.001), and for total IgG4 and total IgM with dyslipidemia (ρ = −0.269 and −0.256, respectively, *p* = 0.001).

### 2.8 Statistical analyses

Univariate statistics were computed as customary (47). Spearman’s rank correlation coefficient (ρ) was used to evaluate correlations between pairs of cytokines, total immunoglobulins and isotype-antigen combinations. Scatterplots of concentrations of cytokines and immunoglobulins in 2020–21 against concentrations in 2016–17 were used to compare individual concentrations between both periods. The paired correlation test was used to compare paired continuous data.

To analyze the intraindividual change of concentrations of cytokines and immunoglobulins between the baseline/prepandemic period (2016–17) and the pandemic period (2020–21), absolute intraindividual change and relative intraindividual change were obtained by computing the difference between the individual concentrations in 2020–21 with respect to the individual concentrations in 2016–17, in absolute terms (pg/mL, μg/mL or MFIs) and in relative terms (percentage of change of concentrations base 10 log-transformed), respectively. The percentage of individuals with a relative increase and with a relative decrease equal to or greater than 15% was also computed using the relative changes of concentrations (base 10 log-transformed) of each cytokine and immunoglobulin.

We computed the number of cytokines in each person with a relative intraindividual change ≥15% as follows: for each subject we added the number of cytokines whose relative change (increase or decrease) in levels from 2016–17 to 2020–201 was equal to or greater than 15%. Similarly, we calculated the number of immunoglobulins in each person with a relative intraindividual change ≥15%.

The relative intraindividual change of cytokine and immunoglobulin concentrations from 2016–17 to 2020–21 was compared between participants who were SARS-CoV-2 seronegative and seropositive, between participants who did and did not develop COVID-19 disease, by sociodemographic variables (sex, age, BMI, tobacco smoking, and educational level), and by comorbidities. Thus, to avoid biases, analyses considered the whole population of 154 persons who were at risk for infection, rather than only the seropositives at risk for COVID-19 (48). Mann–Whitney’s *U* test was used to assess differences in concentrations and differences in relative change.

Cytokine levels measured in samples collected in 2020–21 might be altered by COVID-19 in participants who had developed the disease when their sample was collected. Therefore, for participants who developed COVID-19, we assessed whether the individual concentration and the intraindividual change was related to the interval of time elapsed between onset of COVID-19 and the blood draw in which levels of cytokines (and immunoglobulins) were analyzed. The median of such time interval was 8.4 months (range: 0.6 to 13 months).

All tests were two-tailed. Statistical analyses were conducted using R, version 4.3.3 (Boston, MA, 2024), and SPSS version 22.0.0.0 (IBM SPSS Statistics, Armonk, NY, 2013).

## 3 Results

### 3.1 Intraindividual change in concentrations of cytokines

The median value of the relative intraindividual change in cytokine levels between 2016 and 2020 was <15% for 29 of the 30 cytokines; specifically, between −2.83 and 13.77% (Table 1). However, a value of zero or near zero in such median was compatible, in some cytokines, with a substantial number of participants having an intraindividual increase or decrease ≥15% in the same cytokine. For instance, the mentioned median was 0 for G-CSF while 33% of individuals had an increase in G-CSF ≥ 15 and 16% a decrease in G-CSF ≥ 15%.

**Table 1:** Concentrations of 30 cytokines in 2016–17 and 2020–21.

Cytokine	Concentrations (pg/mL)		Change of concentrations 2016–2020	
	2016–2017	2020–2021	Δ absolute (pg/mL)	Δ relative^a^ (%)
Growth factors				
G-CSF (median)	32.39	36.50	0.00	0.00
(P25, P75)	(<LOQ, 111.7)	(12.70, 129.4)	(−8.50, 41.88)	(−3.92, 23.80)
Geometric mean	31.48	39.37		
∆ ≥ 15%				33.1
∇ ≥ 15%				16.2
EGF (median)	85.28	119.4^b^	28.36	7.15
(P25, P75)	(43.16, 142.4)	(78.74, 173.5)	(−14.75, 74.80)	(−3.06, 20.73)
Geometric mean	82.17	119.4		
∆ ≥ 15%				32.5
∇ ≥ 15%				7.1
FGF (median)	12.90	14.79	0.00	0.00
(P25, P75)	(4.85, 33.57)	(6.26, 31.31)	(−7.00, 7.52)	(−18.09, 22.68)
Geometric mean	14.40	13.81		
∆ ≥ 15%				29.9
∇ ≥ 15%				32.5
GM-CSF (median)	6.98	9.81	0.00	0.00
(P25, P75)	(1.09, 38.19)	(1.09, 38.35)	(−5.32, 10.30)	(−14.44, 32.47)
Geometric mean	6.78	8.41		
∆ ≥ 15%				31.2
∇ ≥ 15%				24.0
HGF (median)	367.4	490.3^b^	103.7	5.42
(P25, P75)	(262.6, 550.1)	(333.3, 734.5)	(15.15, 291.4)	(0.88, 10.21)
Geometric mean	377.7	489.5		
∆ ≥ 15%				9.1
∇ ≥ 15%				3.2
VEGF (median)	5.46	5.97	0.70	6.75
(P25, P75)	(2.57, 8.73)	(3.41, 10.20)	(−0.40, 2.22)	(−8.18, 36.68)
Geometric mean	4.54	5.50		
∆ ≥ 15%				42.9
∇ ≥ 15%				17.5
Chemokines				
IL-8 (median)	16.42	21.22^b^	3.39	8.44
(P25, P75)	(11.58, 24.10)	(14.31, 28.52)	(0.00, 9.46)	(0.00, 16.32)
Geometric mean	16.03	21.14		
∆ ≥ 15%				28.6
∇ ≥ 15%				1.3
IP-10 (median)	6.51	1.06^b^	−5.01	−96.40
(P25, P75)	(4.17, 9.71)	(0.46, 2.45)	(−7.77, −2.73)	(−153.2, −60.50)
Geometric mean	6.46	1.01		
∆ ≥ 15%				4.5
∇ ≥ 15%				90.9
RANTES (median)	3,313	3,264	−1.26	0.00
(P25, P75)	(2,618, 5,970)	(2,521, 4,643)	(−443.0, 132.0)	(−1.88, 0.63)
Geometric mean	3,626	3,400		
∆ ≥ 15%				0.0
∇ ≥ 15%				0.0
EOTAXIN (median)	74.52	75.04	−1.59	−0.51
(P25, P75)	(58.23, 100.8)	(52.46, 101.3)	(−17.50, 13.57)	(−4.89, 4.74)
Geometric mean	74.76	73.73		
∆ ≥ 15%				4.5
∇ ≥ 15%				3.9
MIP-1α (median)	36.29	50.81	0.00	0.00
(P25, P75)	(<LOQ, 109.0)	(<LOQ, 121.7)	(−18.36, 45.41)	(−8.20, 21.71)
Geometric mean	34.34	40.33		
∆ ≥ 15%				27.9
∇ ≥ 15%				19.5
MIP-1β (median)	166.1	205.0	13.08	2.23
(P25, P75)	(90.91, 348.0)	(116.7, 466.3)	(−42.55, 93.55)	(−3.60, 11.15)
Geometric mean	212.5	238.1		
∆ ≥ 15%				15.6
∇ ≥ 15%				6.5
MCP-1 (median)	639.4	654.5	43.13	0.99
(P25, P75)	(466.1, 870.7)	(526.9, 874.8)	(−93.31, 159.1)	(−2.42, 4.01)
Geometric mean	678.9	699.8		
∆ ≥ 15%				0.6
∇ ≥ 15%				1.3
MIG (median)	<LOQ	<LOQ	0.00	0.00
(P25, P75)	(<LOQ, 20.63)	(<LOQ, 20.63)	(0.00, 0.00)	(0.00, 0.00)
Geometric mean	<LOQ	<LOQ		
∆ ≥ 15%				15.6
∇ ≥ 15%				17.5
TH1				
IL-2 (median)	9.45	19.94	1.16	7.12
(P25, P75)	(2.48, 51.20)	(3.96, 61.05)	(−3.90, 23.73)	(−16.77, 54.71)
Geometric mean	13.65	18.04		
∆ ≥ 15%				46.1
∇ ≥ 15%				26.6
IL-12 (median)	56.31	58.08	1.99	0.95
(P25, P75)	(41.20, 87.28)	(37.04, 87.84)	(−13.44, 13.04)	(−5.63, 5.75)
Geometric mean	58.80	56.87		
∆ ≥ 15%				6.5
∇ ≥ 15%				7.8
IFN-γ (median)	<LOQ	<LOQ	0.00	0.00
(P25, P75)	(<LOQ, 0.22)	(<LOQ, <LOQ)	(0.00, 0.00)	(0.00, 0.00)
Geometric mean	0.22	0.22		
∆ ≥ 15%				9.7
∇ ≥ 15%				8.4
TH2				
IL-4 (median)	1.54	<LOQ	0.00	0.00
(P25, P75)	(1.47, 4.87)	(<LOQ, 7.14)	(0.00, 0.00)	(0.00, 0.00)
Geometric mean	3.35	3.60		
∆ ≥ 15%				17.5
∇ ≥ 15%				15.6
IL-5 (median)	<LOQ	<LOQ	0.00	0.00
(P25, P75)	(<LOQ, 1.11)	(<LOQ, 1.11)	(0.00, 0.41)	(0.00, 26.29)
Geometric mean	0.83	0.88		
∆ ≥ 15%				26.6
∇ ≥ 15%				21.4
IL-13 (median)	6.70	6.70	0.00	0.00
(P25, P75)	(<LOQ, 16.22)	(<LOQ, 20.66)	(−4.02, 4.81)	(−13.56, 25.51)
Geometric mean	6.10	6.47		
∆ ≥ 15%				28.6
∇ ≥ 15%				24.0
Pro-inflammatory				
IL-1β (median)	0.92	1.09	0.00	0.00
(P25, P75)	(<LOQ, 2.26)	(0.55, 1.83)	(−0.32, 0.40)	(−18.72, 40.19)
Geometric mean	1.05	1.10		
∆ ≥ 15%				34.4
∇ ≥ 15%				26.6
TNF-α (median)	3.12	4.97	0.93	13.77
(P25, P75)	(1.34, 14.20)	(1.34, 25.75)	(−0.19, 8.59)	(−4.00, 86.00)
Geometric mean	3.97	5.62		
∆ ≥ 15%				48.7
∇ ≥ 15%				20.1
IL-6 (median)	14.10	23.76	1.53	4.53
(P25, P75)	(4.52, 54.29)	(8.28, 67.14)	(−6.86, 14.99)	(−6.95, 35.91)
Geometric mean	16.27	22.64		
∆ ≥ 15%				39.0
∇ ≥ 15%				16.9
IFN-α (median)	17.89	22.05	1.21	0.10
(P25, P75)	(5.41, 52.65)	(7.94, 52.64)	(−7.20, 12.70)	(−8.66, 20.91)
Geometric mean	18.29	21.38		
∆ ≥ 15%				33.8
∇ ≥ 15%				15.6
IL-2R (median)	138.1	149.5	7.99	1.12
(P25, P75)	(50.46, 399.8)	(61.52, 447.7)	(−63.43, 85.15)	(−9.86, 13.41)
Geometric mean	158.6	162.7		
∆ ≥ 15%				22.7
∇ ≥ 15%				16.2
IL-17 (median)	<LOQ	<LOQ	0.00	0.00
(P25, P75)	(<LOQ, 1.56)	(<LOQ, 1.65)	(0.00, 0.92)	(0.00, 30.14)
Geometric mean	<LOQ	<LOQ		
∆ ≥ 15%				26.6
∇ ≥ 15%				18.2
Regulatory				
IL-7 (median)	15.40	17.50	0.00	0.00
(P25, P75)	(<LOQ, 32.51)	(<LOQ, 39.21)	(−5.98, 10.40)	(−6.44, 14.57)
Geometric mean	12.00	13.78		
∆ ≥ 15%				24.7
∇ ≥ 15%				18.2
Anti-inflammatory				
IL-10 (median)	3.02	3.28	0.00	0.00
(P25, P75)	(<LOQ, 19.45)	(<LOQ, 24.10)	(−2.62, 4.44)	(−15.30, 32.51)
Geometric mean	3.79	4.15		
∆ ≥ 15%				29.9
∇ ≥ 15%				25.3
IL-15 (median)	<LOQ	<LOQ	0.00	0.00
(P25, P75)	(<LOQ, 45.30)	(<LOQ, 54.65)	(0.00, 2.34)	(0.00, 0.70)
Geometric mean	9.47	11.50		
∆ ≥ 15%				19.5
∇ ≥ 15%				14.9
IL-1RA (median)	263.9	238.1	−29.94	−2.83
(P25, P75)	(154.3, 515.4)	(118.5, 521.9)	(−121.4, 77.22)	(−9.60, 4.84)
Geometric mean	290.3	255.2		
∆ ≥ 15%				7.8
∇ ≥ 15%				11.7

Absolute intraindividual change and relative intraindividual change (*N* = 154). *Δ absolute:* Difference between the individual concentrations in 2020–21 with respect to the individual concentrations in 2016–17, in absolute terms (pg/mL). Δ relative: Difference between the individual concentrations in 2020–21 with respect to the individual concentrations in 2016–17, in relative terms (percentage of change). P25: percentile 25th. P75: percentile 75th. <LOQ: value lower than the minimum concentration of the corresponding range of the limits of quantification. See Supplementary Table 1. ∆ ≥ 15%: percentage of individuals with a relative increase in the concentrations between 2016 and 2020 equal to or greater than 15%. Includes individuals with concentrations unquantified in 2016 and quantified in 2020 (as explained in Methods, the concentration in 2016 was half the lower limit of quantification). ∇ ≥ 15%: percentage of individuals with a relative decrease in the concentrations between 2016 and 2020 equal to or greater than 15%. Includes individuals with concentrations quantified in 2016 and unquantified in 2020 (as explained in Methods, the concentration in 2020 was half the lower limit of quantification).

a Units used for computing the relative intraindividual change (Δ relative) were base 10 logtransformed pg/mL.

b *p* value <0.05, Mann–Whitney’s U test (two-tail).

More specifically, 15 of the 30 cytokines had a null *median* relative intraindividual change. Twelve other cytokines had a modest positive *median* relative intraindividual change, ranging from 0.10 to 13.77%. Finally, 2 other cytokines had a modest negative *median* relative intraindividual change (EOTAXIN and IL-1RA; Table 1). For 11 cytokines, the *percentage of participants* having a relative intraindividual increase ≥15% ranged from 29.9 to 48.7%. For these 11 cytokines, the percentage of participants with an intraindividual decrease ≥15% ranged from 7.1 to 32.5%. For IP-10, the percentage of participants having a relative intraindividual decrease ≥15% was 90.9%. Thus, for the vast majority of cytokines, either (a) the percentage of participants with an increase in concentrations was similar to the percentage of participants with a decrease in concentrations, or (b) the percentage of participants with an increase was slightly larger than the percentage with a decrease (Table 1). Neither of these two predominant patterns was specific of one type of cytokines. Concentrations in 2016–17 are presented as possible reference values.

**Figure 2:**
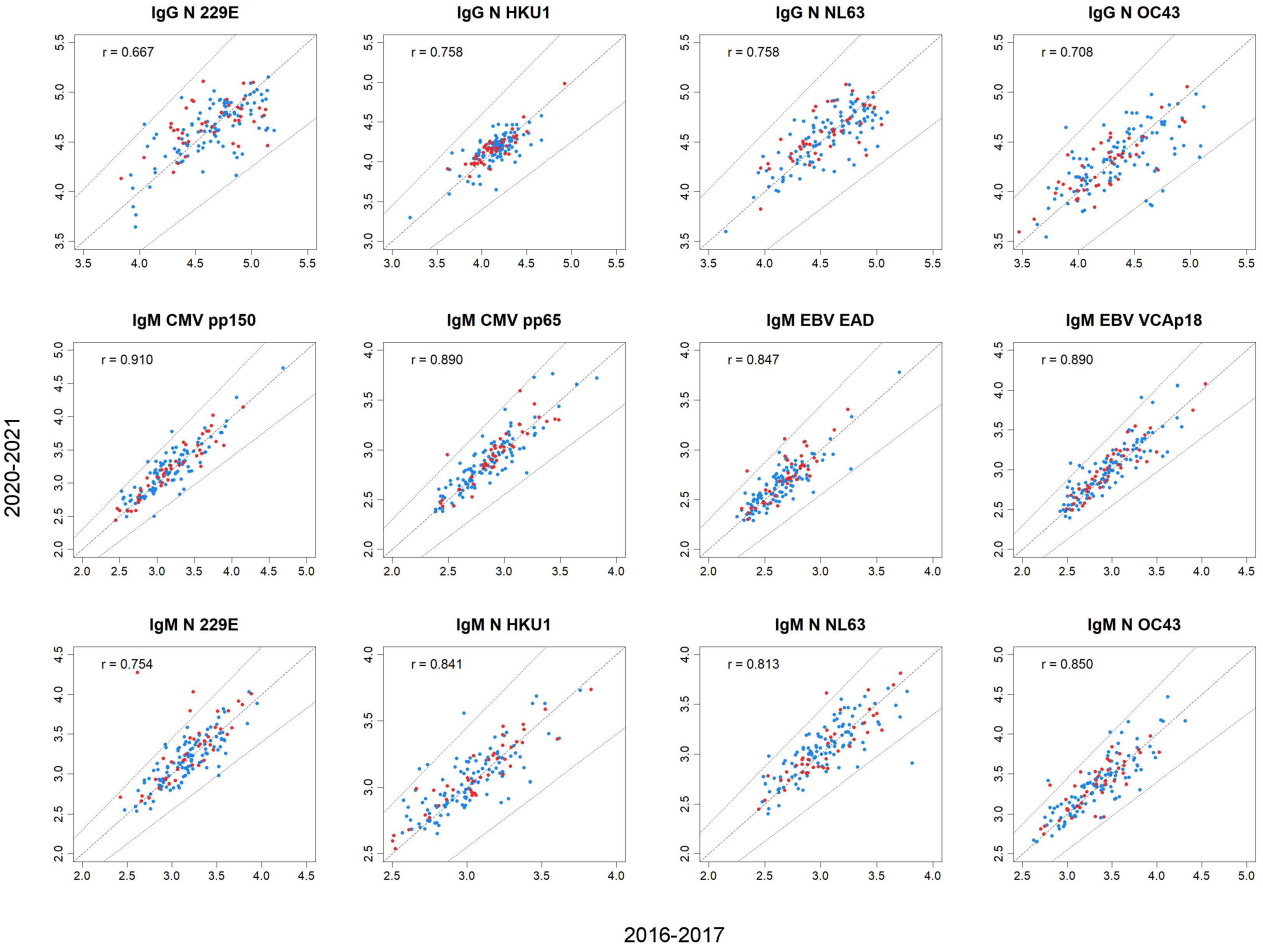
Scatterplots of concentrations (log10, pg./mL) of cytokines in 2020–21 against concentrations in 2016–17 by SARS-CoV-2 seropositivity. Blue dots, seronegative; red dots, seropositive. A 15% change is delimited by the two lines at both sides of the diagonal line indicating no change. All *p*-values <0.001.

No major differences in cytokine intraindividual changes between 2016 and 2020 were observed between participants who were SARS-CoV-2 seronegative and seropositive, nor between participants who did and did not develop COVID-19 disease (Table 2; Figure 2). The difference in the median change was statistically significant in only two instances (IL-8 and IL-4); even then, the difference was null or only slightly higher in SARS-CoV-2 seropositives than in seronegatives. The change tended to be similar (in seropositives and seronegatives, and in participants with and without COVID-19 disease) when using the median of the change and the 4 percentages shown in Table 2: increase (∆) ≥ 15% and decrease (∇) ≥ 15% by outcome. IP-10, the only cytokine that showed a substantial decrease in the overall population (Table 1), showed a highly similar decrease in participants who were SARS-CoV-2 seronegative and seropositive, and in participants who did and did not develop COVID-19.

**Table 2:** Relative intraindividual change (%) of cytokine concentrations from 2016–17 to 2020–21 in participants SARS-CoV-2 seronegative and seropositive, and in participants without COVID-19 disease and with COVID-19 disease.

Cytokine^a^	SARS-CoV-2 status		P^b^	COVID-19 disease		P^b^
	Seronegative (*N* = 104)	Seropositive (*N* = 41)		No COVID (*N* = 134)	COVID (*N* = 20)	
Growth factors						
G-CSF (median)	0.00	0.86	0.439	0.00	0.00	0.489
(P25, P75)	(−5.25, 23.32)	(0.00, 27.34)		(−5.30, 23.30)	(0.00, 34.99)	
∆ ≥ 15%	32.7	36.6		32.8	35.0	
∇ ≥ 15%	16.3	12.2		17.2	10.0	
EGF (median)	7.89	3.01	0.122	7.54	0.64	0.255
(P25, P75)	(−2.56, 20.90)	(−4.63, 13.78)		(−2.64, 20.73)	(−12.04, 24.00)	
∆ ≥ 15%	35.6	22.0		32.8	30.0	
∇ ≥ 15%	4.8	14.6		5.2	20.0	
FGF (median)	0.00	0.00	0.479	0.00	−2.88	0.186
(P25, P75)	(−17.21, 24.71)	(−23.36, 22.33)		(−17.79, 24.33)	(−24.90, 2.32)	
∆ ≥ 15%	30.8	29.3		31.3	20.0	
∇ ≥ 15%	27.9	31.7		29.9	35.0	
GM-CSF (median)	0.00	0.00	0.222	0.00	0.00	0.292
(P25, P75)	(−10.69, 45.56)	(−17.73, 15.64)		(−11.55, 35.13)	(−23.97, 15.38)	
∆ ≥ 15%	34.6	24.4		32.1	25.0	
∇ ≥ 15%	23.1	26.8		23.1	30.0	
HGF (median)	5.95	4.29	0.329	5.65	3.97	0.319
(P25, P75)	(1.83, 10.20)	(−1.86, 9.49)		(1.35, 10.23)	(−3.02, 8.08)	
∆ ≥ 15%	5.8	12.2		9.7	5.0	
∇ ≥ 15%	3.8	2.4		3.0	5.0	
VEGF (median)	8.71	2.30	0.246	7.73	2.05	0.191
(P25, P75)	(−6.51, 39.93)	(−8.66, 23.11)		(−7.97, 39.75)	(−17.63, 21.68)	
∆ ≥ 15%	45.2	41.5		44.8	30.0	
∇ ≥ 15%	13.5	22.0		16.4	25.0	
Chemokines						
IL-8 (median)	5.44	12.04	0.012	7.25	9.67	0.248
(P25, P75)	(−1.13, 16.46)	(4.26, 23.96)		(0.00, 16.32)	(2.49, 21.61)	
∆ ≥ 15%	28.8	34.1		28.4	30.0	
∇ ≥ 15%	1.9	0.0		1.5	0.0	
IP-10 (median)	−96.08	−108.8	0.582	−94.22	−113.7	0.503
(P25, P75)	(−157.6, −68.37)	(−149.7, −42.46)		(−153.2, −60.50)	(−177.5, −60.16)	
∆ ≥ 15%	2.9	9.8		3.7	10.0	
∇ ≥ 15%	93.3	82.9		91.0	90.0	
RANTES (median)	0.00	−0.02	0.829	0.00	−0.01	0.722
(P25, P75)	(−1.72, 0.66)	(−2.70, 0.61)		(−1.89, 0.63)	(−1.89, 1.64)	
∆ ≥ 15%	0.0	0.0		0.0	0.0	
∇ ≥ 15%	0.0	0.0		0.0	0.0	
EOTAXIN (median)	−0.29	−1.60	0.779	−0.16	−1.99	0.378
(P25, P75)	(−4.89, 5.14)	(−4.78, 3.43)		(−4.93, 5.28)	(−4.37, 2.44)	
∆ ≥ 15%	2.9	9.8		5.2	0.0	
∇ ≥ 15%	5.8	0.0		4.5	0.0	
MIP-1α (median)	0.00	0.00	0.571	0.00	0.00	0.199
(P25, P75)	(−6.97, 23.91)	(−9.41, 16.42)		(−4.03, 23.82)	(−20.69, 7.85)	
∆ ≥ 15%	27.9	26.8		29.1	20.0	
∇ ≥ 15%	19.2	19.5		18.7	25.0	
MIP-1β (median)	3.68	0.98	0.683	2.90	0.49	0.425
(P25, P75)	(−4.11, 11.19)	(−3.07, 10.17)		(−3.12, 11.37)	(−5.85, 6.59)	
∆ ≥ 15%	14.4	19.5		15.7	15.0	
∇ ≥ 15%	6.7	4.9		6.7	5.0	
MCP-1 (median)	0.55	2.32	0.424	1.14	−0.17	0.320
(P25, P75)	(−2.74, 3.60)	(−2.66, 4.14)		(−1.86, 4.08)	(−4.59, 3.74)	
∆ ≥ 15%	1.0	0.0		0.7	0.0	
∇ ≥ 15%	1.0	2.4		0.7	5.0	
MIG (median)	0.00	0.00	0.724	0.00	0.00	0.344
(P25, P75)	(0.00, 0.00)	(−11.90, 0.00)		(0.00, 0.00)	(−36.97, 0.00)	
∆ ≥ 15%	17.3	12.2		15.7	15.0	
∇ ≥ 15%	16.3	19.5		16.4	25.0	
TH1						
IL-2 (median)	6.24	7.58	0.887	7.12	4.83	0.604
(P25, P75)	(−16.89, 57.47)	(−13.94, 39.53)		(−16.28, 58.64)	(−32.22, 42.01)	
∆ ≥ 15%	46.2	46.3		46.3	45.0	
∇ ≥ 15%	26.9	24.4		25.4	35.0	
IL-12 (median)	1.37	0.00	0.968	0.60	1.52	0.656
(P25, P75)	(−5.43, 5.64)	(−6.13, 7.28)		(−5.71, 5.51)	(−5.37, 7.36)	
∆ ≥ 15%	5.8	7.3		6.7	5.0	
∇ ≥ 15%	9.6	4.9		8.2	5.0	
IFN-γ (median)	0.00	0.00	0.260	0.00	0.00	0.347
(P25, P75)	(0.00, 0.00)	(0.00, 0.00)		(0.00, 0.00)	(0.00, 0.00)	
∆ ≥ 15%	8.7	14.6		10.4	5.0	
∇ ≥ 15%	9.6	7.3		8.2	10.0	
TH2						
IL-4 (median)	0.00	0.00	0.038	0.00	0.00	0.482
(P25, P75)	(0.00, 0.00)	(0.00, 15.24)		(0.00, 0.00)	(0.00, 0.00)	
∆ ≥ 15%	16.3	24.4		17.2	20.0	
∇ ≥ 15%	16.3	9.8		17.2	5.0	
IL-5 (median)	0.00	0.00	0.847	0.00	0.00	0.898
(P25, P75)	(0.00, 13.80)	(−15.42, 45.86)		(0.00, 20.69)	(−18.57, 50.66)	
∆ ≥ 15%	24.0	34.1		26.1	30.0	
∇ ≥ 15%	19.2	24.4		20.9	25.0	
IL-13 (median)	0.00	0.00	0.878	0.00	0.00	0.793
(P25, P75)	(−11.36, 31.84)	(−12.87, 20.51)		(−13.90, 25.51)	(−13.70, 41.03)	
∆ ≥ 15%	29.8	29.3		29.1	25.0	
∇ ≥ 15%	24.0	22.0		24.6	20.0	
Pro-inflammatory						
IL-1β (median)	0.00	0.00	0.145	0.00	0.00	0.512
(P25, P75)	(−6.67, 42.57)	(−61.24, 0.00)		(−15.98, 40.19)	(−75.74, 62.24)	
∆ ≥ 15%	39.4	22.0		35.8	25.0	
∇ ≥ 15%	23.1	31.7		25.4	35.0	
TNF-α (median)	17.48	12.93	0.975	14.75	0.00	0.698
(P25, P75)	(−6.95, 87.01)	(0.00, 57.90)		(−4.82, 87.12)	(0.00, 57.77)	
∆ ≥ 15%	50.0	48.8		49.3	45.0	
∇ ≥ 15%	21.2	17.1		20.1	20.0	
IL-6 (median)	4.78	1.91	0.806	6.67	0.00	0.182
(P25, P75)	(−7.41, 31.49)	(−2.69, 38.44)		(−6.76, 39.18)	(−13.71, 14.36)	
∆ ≥ 15%	38.5	36.6		41.8	20.0	
∇ ≥ 15%	15.4	17.1		15.7	25.0	
IFN-α (median)	0.10	4.22	0.518	0.10	2.11	0.565
(P25, P75)	(−8.46, 25.19)	(−10.43, 18.87)		(−8.26, 21.80)	(−16.40, 20.70)	
∆ ≥ 15%	36.5	26.8		33.6	35.0	
∇ ≥ 15%	15.4	17.1		14.2	25.0	
IL-2R (median)	1.51	0.00	0.533	1.71	−4.16	0.177
(P25, P75)	(−7.80, 13.30)	(−11.75, 15.05)		(−9.19, 13.79)	(−19.78, 7.83)	
∆ ≥ 15%	22.1	24.4		23.1	20.0	
∇ ≥ 15%	13.5	19.5		14.2	30.0	
IL-17 (median)	0.00	0.00	0.327	0.00	0.00	0.387
(P25, P75)	(0.00, 42.16)	(−0.19, 13.93)		(0.00, 38.42)	(0.00, 0.00)	
∆ ≥ 15%	28.8	24.4		28.4	15.0	
∇ ≥ 15%	17.3	19.5		17.9	20.0	
Regulatory						
IL-7 (median)	0.00	0.00	0.510	0.00	0.00	0.259
(P25, P75)	(−6.29, 13.76)	(−3.95, 27.48)		(−6.06, 14.98)	(−11.82, 12.01)	
∆ ≥ 15%	23.1	29.3		24.6	25.0	
∇ ≥ 15%	18.3	12.2		17.9	20.0	
Anti-inflammatory						
IL-10 (median)	0.00	0.00	0.106	0.00	0.00	0.140
(P25, P75)	(−11.39, 55.37)	(−25.45, 8.32)		(−11.48, 35.17)	(−26.85, 0.00)	
∆ ≥ 15%	32.7	22.0		32.1	15.0	
∇ ≥ 15%	22.1	31.7		23.1	40.0	
IL-15 (median)	0.00	0.00	0.816	0.00	0.00	0.772
(P25, P75)	(0.00, 0.58)	(−1.69, 6.23)		(0.00, 0.57)	(0.00, 18.43)	
∆ ≥ 15%	19.2	22.0		18.7	25.0	
∇ ≥ 15%	13.5	14.6		11.9	20.0	
IL-1RA (median)	−2.47	−3.44	0.458	−2.04	−7.36	0.061
(P25, P75)	(−9.62, 6.01)	(−9.32, 3.10)		(−9.30, 5.93)	(−12.62, −0.33)	
∆ ≥ 15%	7.7	4.9		8.2	5.0	
∇ ≥ 15%	12.5	12.2		10.4	20.0	

a Units used for computing the relative intraindividual change were base 10 logtransformed pg/mL.

b Mann–Whitney’s U test (two-tail).

Among the 20 participants who developed COVID-19, no associations were observed between cytokine concentrations measured in 2020–21 and the time from disease onset to the blood draw in which cytokine concentrations were analyzed (Supplementary Figure 4). Remarkably, the 4 participants with the shortest intervals from disease onset to blood draw (<2 months) did not have higher concentrations of cytokines (Supplementary Figure 5a), neither higher relative intraindividual increases (Supplementary Figure 5b), than participants with longer intervals. Similarly, participants with the longest intervals from disease onset to blood draw did not show lower cytokine concentrations or higher relative decreases.

98% of participants had 4 or more cytokines with a relative intraindividual change (increase or decrease) of concentrations from 2016–17 to 2020–21 ≥ 15%. 21% of participants had between 15 to 27 cytokines with a relative change ≥15%, while no participants had all 30 cytokines with a relative change lower than 15%. The percentage of changes was not different by SARS-CoV-2 infection seropositivity or by COVID-19 disease (Supplementary Table 3).

The intraindividual change in cytokine concentrations from 2016–17 to 2020–21 was similar by sex (Table 3). Only IL-2 increased more in men, although women had higher IL-2 concentrations in 2016–17 than men (Supplementary Table 4). Prepandemic concentrations and intraindividual change in cytokines were also similar across age groups (Supplementary Tables 5a,b), although concentrations of some cytokines were higher in younger participants (e.g., G-CSF, TNF-*α*), and concentrations of others were lower in younger participants (e.g., IL-8, EOTAXIN, MCP-1). Prepandemic concentrations and change were also similar by BMI (Supplementary Tables 6a,b), by tobacco smoking (Supplementary Tables 7a,b), and by educational level (Supplementary Tables 8a,b). Although differences for a few intraindividual changes by age or by smoking were statistically significant, the magnitude of such differences was small. Again, concentrations in 2016–17 are presented as possible reference values in different sociodemographic groups.

**Table 3:** Relative intraindividual change (%) from 2016–17 to 2020–21 of concentrations of cytokines, by sex (*N* = 154).

Cytokine^a^	Men (*N* = 72)	Women (*N* = 82)	P^b^
Growth factors			
G-CSF (median)	0.00	0.00	0.170
(P25, P75)	(0.00, 26.70)	(−13.53, 20.14)	
∆ ≥ 15%	34.7	31.7	
∇ ≥ 15%	6.9	24.4	
EGF (median)	7.21	6.35	0.244
(P25, P75)	(−1.86, 22.35)	(−3.82, 16.81)	
∆ ≥ 15%	37.5	28.0	
∇ ≥ 15%	8.3	6.1	
FGF (median)	0.00	0.00	0.205
(P25, P75)	(−15.24, 25.49)	(−20.19, 20.02)	
∆ ≥ 15%	33.3	26.8	
∇ ≥ 15%	25.0	35.4	
GM-CSF (median)	0.00	0.00	0.771
(P25, P75)	(−13.11, 35.30)	(−16.33, 31.51)	
∆ ≥ 15%	33.3	29.3	
∇ ≥ 15%	22.2	25.6	
HGF (median)	5.32	6.10	0.559
(P25, P75)	(0.86, 9.78)	(1.53, 10.38)	
∆ ≥ 15%	8.3	9.8	
∇ ≥ 15%	2.8	3.7	
VEGF (median)	9.92	4.09	0.523
(P25, P75)	(−8.27, 47.50)	(−7.98, 28.51)	
∆ ≥ 15%	45.8	40.2	
∇ ≥ 15%	19.4	15.9	
Chemokines			
IL-8 (median)	10.35	5.68	0.365
(P25, P75)	(0.08, 16.81)	(−0.26, 14.85)	
∆ ≥ 15%	33.3	24.4	
∇ ≥ 15%	1.4	1.2	
IP-10 (median)	−98.79	−93.31	0.497
(P25, P75)	(−158.9, −63.92)	(−137.1, −60.19)	
∆ ≥ 15%	6.9	2.4	
∇ ≥ 15%	88.9	92.7	
RANTES (median)	0.00	−0.45	0.066
(P25, P75)	(−1.40, 0.62)	(−2.73, 0.64)	
∆ ≥ 15%	0.0	0.0	
∇ ≥ 15%	0.0	0.0	
EOTAXIN (median)	−0.57	−0.51	0.891
(P25, P75)	(−5.10, 5.14)	(−4.60, 4.27)	
∆ ≥ 15%	4.2	4.9	
∇ ≥ 15%	2.8	4.9	
MIP-1β (median)	3.77	0.61	0.115
(P25, P75)	(−2.97, 13.74)	(−3.91, 7.42)	
∆ ≥ 15%	18.1	13.4	
∇ ≥ 15%	4.2	8.5	
MCP-1 (median)	1.41	0.76	0.643
(P25, P75)	(−1.73, 4.33)	(−2.6, 3.43)	
∆ ≥ 15%	1.4	0.0	
∇ ≥ 15%	1.4	1.2	
TH1			
IL-2 (median)	21.49	0.00	0.029
(P25, P75)	(−10.80, 66.76)	(−21.33, 36.26)	
∆ ≥ 15%	55.6	37.8	
∇ ≥ 15%	20.8	31.7	
IL-12 (median)	0.97	0.69	0.937
(P25, P75)	(−5.88, 5.41)	(−5.50, 6.01)	
∆ ≥ 15%	8.3	4.9	
∇ ≥ 15%	9.7	6.1	
Pro-inflammatory			
IL-1β (median)	0.00	0.00	0.079
(P25, P75)	(−3.90, 60.81)	(−32.29, 29.28)	
∆ ≥ 15%	41.7	28.0	
∇ ≥ 15%	23.6	29.3	
TNF-α (median)	22.21	6.41	0.262
(P25, P75)	(0.00, 82.08)	(−16.5, 86.98)	
∆ ≥ 15%	52.8	45.1	
∇ ≥ 15%	12.5	26.8	
IL-6 (median)	11.98	3.76	0.150
(P25, P75)	(−5.92, 71.89)	(−7.6, 21.98)	
∆ ≥ 15%	43.1	35.4	
∇ ≥ 15%	16.7	17.1	
IFN-α (median)	5.00	0.00	0.280
(P25, P75)	(−3.59, 23.50)	(−11.83, 20.74)	
∆ ≥ 15%	37.5	30.5	
∇ ≥ 15%	15.3	15.9	
IL-2R (median)	2.70	−0.95	0.026
(P25, P75)	(−4.45, 17.10)	(−13.23, 11.12)	
∆ ≥ 15%	29.2	17.1	
∇ ≥ 15%	11.1	20.7	
Regulatory			
IL-7 (median)	0.00	0.00	0.222
(P25, P75)	(−6.15, 30.31)	(−8.94, 12.49)	
∆ ≥ 15%	31.9	18.3	
∇ ≥ 15%	18.1	18.3	
Anti-inflammatory			
IL-1RA (median)	−2.83	−2.38	0.696
(P25, P75)	(−8.89, 5.97)	(−10.40, 4.33)	
∆ ≥ 15%	11.1	4.9	
∇ ≥ 15%	12.5	11.0	

Cytokines quantified in more than 70% of participants. P25: percentile 25th. P75: percentile 75th.

a Units used for computing the relative intraindividual change were base 10 logtransformed pg/mL.

b Mann–Whitney’s U test (two-tail).

### 3.2 Intraindividual change in concentrations of immunoglobulins against viral exposures and of total immunoglobulins

The *median* value of the relative intraindividual change in immunoglobulin levels between 2016 and 2020 was virtually null for the 24 isotype-antigen combinations for CMV, EBV, and human common cold coronaviruses (HCoV-229E, HCoV-OC43, HCoV-NL63 i HCoV-HKU1) and for the seven total immunoglobulins (as well as for the sum of IgG 1–4 subclasses). *Median* values of the relative change ranged from −0.46% for IgA NL63 to 2.61% for total IgE (Table 4). Similarly, the *percentage of participants* having a relative intraindividual change (increase or decrease) ≥ 15% was virtually null for all immunoglobulins (total and virus-specific), ranging from 0.0 to 6.5%, except for total IgE, for which 17% of participants had a relative intraindividual increase ≥15 and 9% of participants had a relative decrease ≥15%. The highest median absolute intraindividual changes were observed for IgGs, the immunoglobulin isotype with the highest concentrations.

**Table 4:** Concentrations of 24 isotype-antigen combinations for cytomegalovirus, Epstein–Barr and common cold infections, and of total Igs from 2016–17 to 2020–21.

Immunoglobulin	Concentrations (MFI)		Change of concentrations 2016–2020	
	2016–2017	2020–2021	Δ absolute (MFI)	Δ relative^a^ (%)
IgA				
IgA CMV pp150 (median)	504.0	540.0	11.25	0.37
(P25, P75)	(357.9, 832.5)	(380.8, 876.5)	(−89.63, 104.1)	(−2.36, 3.38)
Geometric mean	622.4	665.5		
∆ ≥ 15%				4.5
∇ ≥ 15%				0.6
IgA CMV pp65 (median)	599.5	650.3	28.00	0.96
(P25, P75)	(422.2, 867.4)	(447.9, 876.1)	(−92.13, 152.9)	(−2.30, 3.65)
Geometric mean	692.2	732.3		
∆ ≥ 15%				1.9
∇ ≥ 15%				0.6
IgA EBV EAD (median)	442.5	463.5	7.00	0.34
(P25, P75)	(304.8, 637.8)	(331.8, 717.5)	(−94.38, 90.13)	(−2.29, 3.69)
Geometric mean	500.7	516.1		
∆ ≥ 15%				1.3
∇ ≥ 15%				1.3
IgA VCAp18 (median)	492.8	512.5	17.50	0.62
(P25, P75)	(349.8, 759.8)	(373.8, 844.8)	(−63.13, 121.4)	(−2.08, 3.48)
Geometric mean	584.4	615.9		
∆ ≥ 15%				1.9
∇ ≥ 15%				0.6
IgA N 229E (median)	1,366	1,316	−20.00	−0.32
(P25, P75)	(746.1, 3,534)	(813.6, 3,102)	(−614.8, 359.4)	(−3.80, 3.45)
Geometric mean	1813	1,693		
∆ ≥ 15%				2.6
∇ ≥ 15%				5.2
IgA N HKU1 (median)	728.8	767.5	29.25	0.91
(P25, P75)	(476.4, 1,186)	(501.8, 1,343)	(−120.5, 159.5)	(−2.51, 3.11)
Geometric mean	854.5	900.8		
∆ ≥ 15%				2.6
∇ ≥ 15%				0.0
IgA NL 63 (median)	1,470	1,610	−39.25	−0.46
(P25, P75)	(780.1, 4,399)	(842.8, 4,143)	(−738.0, 343.6)	(−3.76, 4.54)
Geometric mean	2008	1986		
∆ ≥ 15%				6.5
∇ ≥ 15%				4.5
IgA N OC43 (median)	1,492	1,541	34.25	0.39
(P25, P75)	(916.5, 2,900)	(998.8, 2,785)	(−271.1, 399.4)	(−2.74, 3.18)
Geometric mean	1758	1818		
∆ ≥ 15%				2.6
∇ ≥ 15%				0.6
IgG				
IgG CMV pp150 (median)	15,503	18,515	1,201	1.40
(P25, P75)	(5,506, 29,463)	(5,842, 38,776)	(−1,066, 9,371)	(−1.36, 3.99)
Geometric mean	13,823	15,842		
∆ ≥ 15%				1.9
∇ ≥ 15%				0.0
IgG CMV pp65 (median)	6,902	7,422	472.5	0.81
(P25, P75)	(4,665, 10,348)	(4,770, 10,334)	(−1,393, 2013)	(−1.92, 3.13)
Geometric mean	6,811	7,237		
∆ ≥ 15%				0.6
∇ ≥ 15%				0.0
IgG EBV EAD (median)	7,021	6,891	279.0	0.49
(P25, P75)	(4,939, 9,931)	(5,372, 9,830)	(−1790, 1800)	(−2.25, 3.06)
Geometric mean	7,041	7,116		
∆ ≥ 15%				0.0
∇ ≥ 15%				0.0
IgG VCAp18 (median)	8,049	8,612	654.3	0.69
(P25, P75)	(4,280, 14,355)	(4,842, 16,546)	(−1,011, 2,890)	(−1.93, 3.39)
Geometric mean	8,936	9,703		
∆ ≥ 15%				1.9
∇ ≥ 15%				0.6
IgG N 229E (median)	46,792	46,290	766.3	0.15
(P25, P75)	(26,485, 75,860)	(29,671, 68,957)	(−11,162, 12,147)	(−2.56, 2.70)
Geometric mean	43,851	43,610		
∆ ≥ 15%				0.6
∇ ≥ 15%				0.0
IgG N HKU1 (median)	14,316	14,930	727.8	0.63
(P25, P75)	(10,244, 18,907)	(11,510, 18,319)	(−2,581, 2,936)	(−1.57, 2.40)
Geometric mean	13,970	14,393		
∆ ≥ 15%				0.0
∇ ≥ 15%				0.0
IgG NL 63 (median)	38,033	38,897	−68.75	−0.04
(P25, P75)	(22,612, 61,267)	(23,036, 56,672)	(−8,797, 8,325)	(−2.45, 2.58)
Geometric mean	35,810	36,023		
∆ ≥ 15%				0.0
∇ ≥ 15%				0.0
IgG N OC43 (median)	19,501	20,370	167.8	0.15
(P25, P75)	(11,251, 36,874)	(11,784, 34,252)	(−6,438, 5,787)	(−3.02, 3.03)
Geometric mean	20,837	20,054		
∆ ≥ 15%				0.6
∇ ≥ 15%				2.6
IgM				
IgM CMV pp150 (median)	1,303	1,451	76.00	1.01
(P25, P75)	(788.9, 2,301)	(778.0, 2,600)	(−149.9, 404.1)	(−2.06, 3.72)
Geometric mean	1,406	1,497		
∆ ≥ 15%				1.3
∇ ≥ 15%				0.6
IgM CMV pp65 (median)	690.3	705.8	22.50	0.60
(P25, P75)	(466.8, 1,039)	(482.9, 1,090)	(−79.50, 155.5)	(−1.73, 3.63)
Geometric mean	718.5	764.8		
∆ ≥ 15%				0.6
∇ ≥ 15%				0.0
IgM EBV EAD (median)	429.5	456.3	12.00	0.51
(P25, P75)	(296.5, 590.2)	(311.6, 621.9)	(−52.50, 81.25)	(−2.25, 3.66)
Geometric mean	445.0	464.5		
∆ ≥ 15%				1.3
∇ ≥ 15%				0.0
IgM VCAp18 (median)	853.3	947.0	51.75	1.11
(P25, P75)	(495.5, 1,482)	(561.6, 1,565)	(−90.00, 282.6)	(−2.33, 4.08)
Geometric mean	901.4	977.5		
∆ ≥ 15%				1.9
∇ ≥ 15%				0.0
IgM N 229E (median)	1,500	1,641	73.00	0.87
(P25, P75)	(866.9, 2,419)	(880.3, 2,618)	(−322.1, 490.6)	(−2.96, 4.52)
Geometric mean	1,483	1,636		
∆ ≥ 15%				2.6
∇ ≥ 15%				0.6
IgM N HKU1 (median)	1,032	1,103	48.50	0.85
(P25, P75)	(700.8, 1,598)	(781.8, 1,648)	(−117.3, 271.9)	(−1.42, 3.86)
Geometric mean	1,061	1,147		
∆ ≥ 15%				1.9
∇ ≥ 15%				0.0
IgM NL 63 (median)	1,023	1,067	72.25	1.12
(P25, P75)	(668.0, 1,597)	(724.4, 1,661)	(−160.4, 339.5)	(−2.54, 4.37)
Geometric mean	1,053	1,120		
∆ ≥ 15%				1.3
∇ ≥ 15%				0.6
IgM N OC43 (median)	2,134	2,279	53.75	0.67
(P25, P75)	(1,188, 3,516)	(1,184, 3,685)	(−342.9, 657.5)	(−2.63, 4.27)
Geometric mean	2,140	2,257		
∆ ≥ 15%				1.9
∇ ≥ 15%				0.0
Total immunoglobulin				
IgG1 (median)	6,175	6,867	588.6	1.05
(P25, P75)	(5,050, 8,691)	(5,455, 9,168)	(−1,101, 1860)	(−1.55, 3.21)
Geometric mean	6,518	7,020		
∆ ≥ 15%				0.0
∇ ≥ 15%				0.0
IgG2 (median)	3,147	3,196	179.6	0.71
(P25, P75)	(2,480, 3,719)	(2,679, 3,905)	(−154.2, 489.3)	(−0.64, 2.12)
Geometric mean	3,072	3,248		
∆ ≥ 15%				0.0
∇ ≥ 15%				0.0
IgG3 (median)	1,289	1,225	−23.11	−0.28
(P25, P75)	(1,038, 1,537)	(1,022, 1,444)	(−228.6, 135.7)	(−2.65, 1.65)
Geometric mean	1,242	1,187		
∆ ≥ 15%				0.0
∇ ≥ 15%				0.0
IgG4 (median)	221.9	231.4	11.83	1.00
(P25, P75)	(123.9, 405.6)	(139.0, 424.0)	(−20.63, 47.45)	(−1.72, 4.46)
Geometric mean	229.2	245.3		
∆ ≥ 15%				1.3
∇ ≥ 15%				0.6
Sum of IgGs (median)	11,361	11,946	670.3	0.64
(P25, P75)	(9,364, 14,489)	(9,920, 14,370)	(−1,122, 2,488)	(−1.01, 2.35)
Geometric mean	11,396	12,091		
∆ ≥ 15%				0.0
∇ ≥ 15%				0.0
IgE (median)	0.23	0.24	0.01	2.61
(P25, P75)	(0.15, 0.35)	(0.16, 0.37)	(−0.01, 0.04)	(−3.91, 11.04)
Geometric mean	0.23	0.25		
∆ ≥ 15%				16.9
∇ ≥ 15%				9.1
IgA (median)	249.8	265.8	11.37	0.92
(P25, P75)	(195.9, 300.6)	(206.8, 319.9)	(−22.74, 47.55)	(−1.74, 3.71)
Geometric mean	243.9	257.4		
∆ ≥ 15%				0.6
∇ ≥ 15%				0.0
IgM (median)	935.5	972.8	26.02	0.32
(P25, P75)	(727.7, 1,387)	(752.2, 1,433)	(−105.8, 186.0)	(−1.51, 3.34)
Geometric mean	985.6	1,038		
∆ ≥ 15%				0.0
∇ ≥ 15%				0.0

Absolute intraindividual change and relative intraindividual change (*N* = 154). Δ absolute: Difference between the individual concentrations in 2020–21 with respect to the individual concentrations in 2016–17, in absolute terms (MFIs for 24 isotype-antigen combinations, μg/mL for total immunoglobulins). Δ relative: Difference between the individual concentrations in 2020–21 with respect to the individual concentrations in 2016–17, in relative terms (percentage of change). P25: percentile 25th. P75: percentile 75th. <LOQ: value lower than the minimum concentration of the corresponding range of the limits of quantification. See Supplementary Table 1. ∆ ≥ 15%: percentage of individuals with a relative increase in the concentrations between 2016 and 2020 equal to or greater than 15%. ∇ ≥ 15%: percentage of individuals with a relative decrease in the concentrations between 2016 and 2020 equal to or greater than 15%.

a Units used for computing the relative intraindividual change were base 10 logtransformed MFI for levels of isotype-antigen combinations, and base 10 logtransformed μg/mL for levels of total Igs.

**Figure 3:**
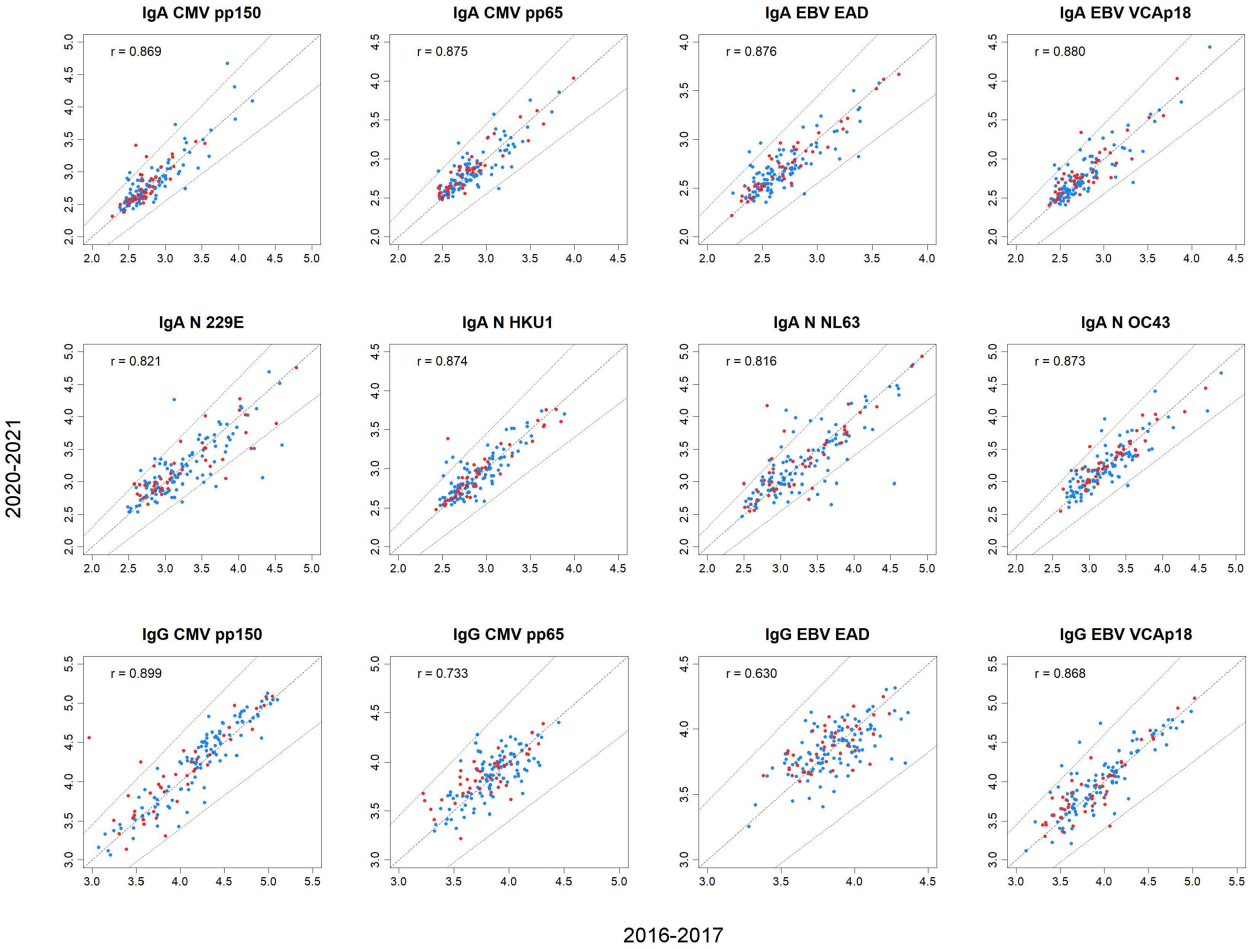
Scatterplots of concentrations (log10, MFI, μg/mL) of immunoglobulins in 2020–21 against concentrations in 2016–17 by SARS-CoV-2 seropositivity. Blue dots, seronegative; red dots, seropositive. A 15% change is delimited by the two lines at both sides of the diagonal line indicating no change. All *p*-values <0.001.

No major differences in intraindividual changes in immunoglobulin levels (in isotype-antigen combinations and in total immunoglobulins) were observed between participants who were SARS-CoV-2 seronegative and seropositive, nor between participants who did and did not develop COVID-19 disease (Table 5; Figure 3). The change tended to be similar (in seropositives and seronegatives, in participants who did and did not develop COVID-19 disease) when using the median of the change and the 4 percentages (∆ ≥ 15% and ∇ ≥ 15%).

**Table 5:** Relative intraindividual change (%) of 24 isotype-antigen combinations for cytomegalovirus, Epstein–Barr and common cold infections, and of total Igs concentrations from 2016–17 to 2020–21 in participants SARS-CoV-2 seronegative and seropositive, and in participants without COVID-19 disease and with COVID-19 disease.

Immunoglobulin^a^	SARS-CoV-2 status		P^b^	COVID-19 disease		P^b^
	Seronegative (*N* = 104)	Seropositive (*N* = 41)		No COVID (*N* = 134)	COVID (*N* = 20)	
IgA						
IgA CMV pp150 (median)	0.25	0.41	0.777	0.37	0.18	0.809
(P25, P75)	(−2.86, 3.38)	(−2.18, 3.06)		(−2.66, 3.43)	(−2.04, 3.09)	
∆ ≥ 15%	4.8	4.9		4.5	5.0	
∇ ≥ 15%	1.0	0.0		0.7	0.0	
IgA CMV pp65 (median)	0.50	1.55	0.139	0.77	1.60	0.275
(P25, P75)	(−2.86, 3.31)	(−0.90, 4.08)		(−2.49, 3.82)	(−0.91, 3.63)	
∆ ≥ 15%	2.9	0.0		2.2	0.0	
∇ ≥ 15%	1.0	0.0		0.7	0.0	
IgA EBV EAD (median)	0.08	0.16	0.450	0.48	0.00	0.522
(P25, P75)	(−2.63, 3.85)	(−0.90, 3.75)		(−2.34, 3.55)	(−1.45, 4.00)	
∆ ≥ 15%	1.9	0.0		1.5	0.0	
∇ ≥ 15%	1.9	0.0		1.5	0.0	
IgA VCAp18 (median)	0.13	1.35	0.254	0.50	1.08	0.220
(P25, P75)	(−2.48, 3.13)	(−1.63, 4.34)		(−2.50, 3.46)	(0.18, 5.27)	
∆ ≥ 15%	1.9	2.4		2.2	0.0	
∇ ≥ 15%	1.0	0.0		0.7	0.0	
IgA N 229E (median)	−0.58	−0.02	0.409	−0.12	−0.93	0.830
(P25, P75)	(−4.40, 3.21)	(−3.03, 4.95)		(−3.70, 3.36)	(−4.40, 5.16)	
∆ ≥ 15%	2.9	2.4		3.0	0.0	
∇ ≥ 15%	5.8	4.9		6.0	0.0	
IgA N HKU1 (median)	0.64	1.21	0.871	0.92	0.56	0.367
(P25, P75)	(−2.65, 3.39)	(−1.98, 2.22)		(−2.36, 3.32)	(−2.94, 1.64)	
∆ ≥ 15%	2.9	2.4		2.2	5.0	
∇ ≥ 15%	0.0	0.0		0.0	0.0	
IgA NL 63 (median)	−0.19	−0.78	0.617	−0.28	−1.05	0.855
(P25, P75)	(−4.62, 4.84)	(−3.44, 4.33)		(−3.76, 4.74)	(−4.35, 4.09)	
∆ ≥ 15%	5.8	9.8		6.0	10.0	
∇ ≥ 15%	4.8	4.9		5.2	0.0	
IgA N OC43 (median)	−0.44	0.85	0.139	0.39	0.51	0.408
(P25, P75)	(−3.78, 3.23)	(−1.04, 3.73)		(−2.86, 2.89)	(−2.00, 4.44)	
∆ ≥ 15%	2.9	2.4		2.2	5.0	
∇ ≥ 15%	1.0	0.0		0.7	0.0	
IgG						
IgG CMV pp150 (median)	1.40	2.11	0.499	1.17	2.12	0.169
(P25, P75)	(−1.38, 4.00)	(−1.28, 4.66)		(−1.55, 3.94)	(0.04, 5.26)	
∆ ≥ 15%	0.0	4.9		1.5	5.0	
∇ ≥ 15%	0.0	0.0		0.0	0.0	
IgG CMV pp65 (median)	0.33	2.08	0.039	0.67	1.98	0.384
(P25, P75)	(−2.48, 2.78)	(−1.33, 5.71)		(−2.03, 3.50)	(−1.75, 2.73)	
∆ ≥ 15%	1.0	0.0		0.7	0.0	
∇ ≥ 15%	0.0	0.0		0.0	0.0	
IgG EBV EAD (median)	0.30	1.51	0.102	0.37	1.38	0.405
(P25, P75)	(−3.06, 2.81)	(−1.38, 4.09)		(−2.32, 2.88)	(−1.21, 3.72)	
∆ ≥ 15%	0.0	0.0		0.0	0.0	
∇ ≥ 15%	0.0	0.0		0.0	0.0	
IgG VCAp18 (median)	0.62	1.32	0.422	0.62	1.37	0.573
(P25, P75)	(−2.25, 3.41)	(−1.08, 4.21)		(−1.93, 3.23)	(−3.14, 4.27)	
∆ ≥ 15%	2.9	0.0		2.2	0.0	
∇ ≥ 15%	0.0	2.4		0.7	0.0	
IgG N 229E (median)	0.06	1.43	0.189	0.15	0.30	0.957
(P25, P75)	(−2.49, 2.26)	(−2.62, 5.62)		(−2.56, 2.63)	(−2.90, 3.01)	
∆ ≥ 15%	1.0	0.0		0.7	0.0	
∇ ≥ 15%	0.0	0.0		0.0	0.0	
IgG N HKU1 (median)	0.34	1.02	0.319	0.41	0.94	0.302
(P25, P75)	(−2.28, 2.50)	(−1.10, 2.48)		(−1.63, 2.37)	(−0.70, 2.78)	
∆ ≥ 15%	0.0	0.0		0.0	0.0	
∇ ≥ 15%	0.0	0.0		0.0	0.0	
IgG NL 63 (median)	−0.18	1.31	0.084	−0.04	0.55	0.648
(P25, P75)	(−2.88, 2.42)	(−1.51, 4.28)		(−2.48, 2.68)	(−2.33, 2.50)	
∆ ≥ 15%	0.0	2.4		0.0	0.0	
∇ ≥ 15%	0.0	0.0		0.0	0.0	
IgG N OC43 (median)	0.04	0.30	0.453	−0.06	1.01	0.369
(P25, P75)	(−3.93, 3.02)	(−2.39, 3.38)		(−3.56, 3.03)	(−0.94, 3.16)	
∆ ≥ 15%	1.0	0.0		0.7	0.0	
∇ ≥ 15%	3.8	0.0		3.0	0.0	
IgM						
IgM CMV pp150 (median)	1.14	0.80	0.645	1.25	−0.19	0.305
(P25, P75)	(−2.00, 4.69)	(−2.13, 3.26)		(−1.85, 4.01)	(−2.99, 3.62)	
∆ ≥ 15%	1.9	0.0		1.5	0.0	
∇ ≥ 15%	1.0	0.0		0.7	0.0	
IgM CMV pp65 (median)	0.34	0.75	0.881	0.55	0.85	0.961
(P25, P75)	(−2.07, 3.92)	(−1.32, 2.85)		(−1.87, 3.81)	(−0.99, 2.09)	
∆ ≥ 15%	0.0	2.4		0.7	0.0	
∇ ≥ 15%	0.0	0.0		0.0	0.0	
IgM EBV EAD (median)	0.63	0.09	0.607	0.53	−0.06	0.801
(P25, P75)	(−2.88, 3.50)	(−1.20, 3.85)		(−2.50, 3.73)	(−1.52, 2.52)	
∆ ≥ 15%	0.0	4.9		1.5	0.0	
∇ ≥ 15%	0.0	0.0		0.0	0.0	
IgM VCAp18 (median)	0.98	1.38	0.944	1.11	1.15	0.813
(P25, P75)	(−2.26, 4.05)	(−2.35, 4.23)		(−2.33, 4.02)	(−2.10, 5.07)	
∆ ≥ 15%	2.9	0.0		2.2	0.0	
∇ ≥ 15%	0.0	0.0		0.0	0.0	
IgM N 229E (median)	0.19	1.66	0.080	0.66	1.57	0.644
(P25, P75)	(−3.43, 4.17)	(−1.23, 4.97)		(−3.28, 4.60)	(−0.13, 2.80)	
∆ ≥ 15%	1.0	7.3		3.0	0.0	
∇ ≥ 15%	1.0	0.0		0.7	0.0	
IgM N HKU1 (median)	0.71	1.58	0.843	0.69	1.60	0.847
(P25, P75)	(−1.82, 4.33)	(−1.35, 2.63)		(−1.63, 4.18)	(−1.15, 2.81)	
∆ ≥ 15%	2.9	0.0		2.2	0.0	
∇ ≥ 15%	0.0	0.0		0.0	0.0	
IgM NL 63 (median)	1.12	0.48	0.399	1.27	0.29	0.384
(P25, P75)	(−2.78, 5.40)	(−2.58, 3.71)		(−2.57, 5.08)	(−2.29, 3.46)	
∆ ≥ 15%	1.0	2.4		1.5	0.0	
∇ ≥ 15%	1.0	0.0		0.7	0.0	
IgM N OC43 (median)	0.43	1.19	0.440	0.53	1.27	0.719
(P25, P75)	(−2.68, 4.20)	(−1.43, 4.32)		(−2.63, 4.37)	(−2.67, 3.48)	
∆ ≥ 15%	1.9	2.4		1.5	5.0	
∇ ≥ 15%	0.0	0.0		0.0	0.0	
Total Immunoglobulin						
IgG1 (median)	1.12	1.07	0.696	0.79	1.93	0.026
(P25, P75)	(−1.66, 3.23)	(−1.01, 3.97)		(−1.88, 3.00)	(0.79, 5.09)	
∆ ≥ 15%	0.0	0.0		0.0	0.0	
∇ ≥ 15%	0.0	0.0		0.0	0.0	
IgG2 (median)	0.79	0.52	0.775	0.61	0.82	0.468
(P25, P75)	(−0.80, 2.47)	(−0.52, 1.61)		(−0.68, 2.12)	(−0.43, 2.47)	
∆ ≥ 15%	0.0	0.0		0.0	0.0	
∇ ≥ 15%	0.0	0.0		0.0	0.0	
IgG3 (median)	−0.62	−0.01	0.920	−0.62	0.33	0.171
(P25, P75)	(−2.31, 1.51)	(−3.51, 1.77)		(−2.65, 1.53)	(−2.10, 1.81)	
∆ ≥ 15%	0.0	0.0		0.0	0.0	
∇ ≥ 15%	0.0	0.0		0.0	0.0	
IgG4 (median)	0.73	1.05	0.745	1.08	0.71	0.772
(P25, P75)	(−1.82, 4.52)	(−1.76, 4.43)		(−1.72, 4.42)	(−2.63, 6.09)	
∆ ≥ 15%	1.0	2.4		1.5	0.0	
∇ ≥ 15%	1.0	0.0		0.7	0.0	
Sum of IgGs (median)	0.79	0.55	0.864	0.59	1.93	0.038
(P25, P75)	(−1.34, 2.32)	(−0.83, 2.97)		(−1.40, 2.19)	(−0.49, 3.68)	
∆ ≥ 15%	0.0	0.0		0.0	0.0	
∇ ≥ 15%	0.0	0.0		0.0	0.0	
IgE (median)	2.57	2.64	0.414	2.80	2.13	0.679
(P25, P75)	(−3.79, 9.26)	(−4.83, 16.56)		(−3.65, 10.64)	(−4.62, 20.48)	
∆ ≥ 15%	14.4	26.8		14.9	30	
∇ ≥ 15%	10.6	4.9		10.4	0.0	
IgA (median)	0.90	0.98	0.375	0.90	1.16	0.458
(P25, P75)	(−2.54, 3.62)	(−1.61, 5.32)		(−2.50, 3.71)	(−1.49, 4.72)	
∆ ≥ 15%	1.0	0.0		0.7	0.0	
∇ ≥ 15%	0.0	0.0		0.0	0.0	
IgM (median)	0.16	0.89	0.083	0.24	1.21	0.072
(P25, P75)	(−1.98, 2.95)	(−0.56, 4.58)		(−1.78, 3.22)	(−0.41, 5.20)	
∆ ≥ 15%	0.0	0.0		0.0	0.0	
∇ ≥ 15%	0.0	0.0		0.0	0.0	

a Units used for computing the relative intraindividual change were base 10 logtransformed MFI for levels of isotype-antigen combinations, and base 10 logtransformed μg/mL for levels of total Igs.

b Mann–Whitney’s U test (two-tail).

Differences in prepandemic levels of total IgM and of IgMs against viral exposures were observed between men and women and across age groups: levels of total IgM and all eight IgMs against CMV, EBV, and HCoV were statistically significantly higher in women than men, and in younger groups than in older participants (Supplementary Tables 9a and 10a). Differences were also observed in prepandemic levels of IgMs by BMI and tobacco smoking: higher concentrations of IgMs were observed in normal weight participants than in overweight or obese participants (Supplementary Table 11a; Supplementary Figure 4b), while lower concentrations were found in former smokers (Supplementary Table 12a). Nevertheless, no differences by sex, age, BMI or smoking were found in intraindividual changes from 2016–17 to 2020–21 in levels of IgMs (Supplementary Tables 9b, 10b, 11b, 12b).

Higher prepandemic levels of total IgGs were also observed in women, and in younger participants. Younger participants also had higher levels of IgAs against CMV and EBV (Supplementary Table 10a); while higher levels of IgA CMV pp65 and IgA EBV EAD were observed in obese participants (Supplementary Table 11a). Again, virtually no significant differences in the intraindividual change of levels of the mentioned immunoglobulins were found by sex, age or BMI (Supplementary Tables 9b, 10b, 11b). Higher intraindividual changes of total immunoglobulins were observed in participants with dyslipidemia (Supplementary Table 13). Lower levels of IgG CMV pp150 were found in participants with higher education. No other significant differences in prepandemic levels or in their change were observed by educational level (Supplementary Tables 14a,b).

## 4 Discussion

Intraindividual changes in concentrations of cytokines and immunoglobulins during the period from 2016–17 to 2020–21 were moderate –perhaps surprisingly so, given the epidemiological context. But maybe less surprisingly, since the long-term stability of an individual’s immune system in the absence of immunological challenge does not require that stability is maintained during infection (4). The 4-year stability suggests that in spite of brief changes due to infections o reactivations of viruses such as EBV, or vaccinations, concentrations return fairly quickly to basal levels. Results thus indicate a rather stable basal state of the immune system (3, 4). This study appears to be the first to document this relative stability of cytokine blood biomarkers in a general, non-institutionalized population, during a period that precedes and includes the pandemic, and with repeated blood samples from the same individuals.

Immune biomarker stability could have implications for clinical and public health practice: it might contribute to personalized preventive strategies, informing on patient vaccine or treatment responsiveness, or serve for patient monitoring in infectious disease contexts.

Previous studies have shown that many individuals have stable immune systems. This was reported by Brodin et al., for instance, in 99 healthy adults and 210 healthy twins (2, 3). Certain functional units of immunity, involving cytokines, vary across individuals primarily as a consequence of non-heritable factors, suggesting that the immune system of healthy individuals is much shaped by the environment, including chronic viral infection as well as socioeconomic factors as cohabitation and housing conditions (2–5).

Cytokine concentrations measured in 2020–21 were not associated with the time interval from disease onset to blood draw among the 20 participants who developed COVID-19 (Supplementary Figure 4; the interval was less than 2 months in only 4 of the 20 individuals, and in such 4 participants concentrations were not particularly increased). The observation suggests that factors other than that interval, such as the basal status of the immune system, might play a more significant role in determining cytokine concentrations post-infection. The present report did not aim at analyzing the long-term consequences of COVID-19 on the immunological system.

In addition to the immunological interest, the result mentioned in the previous paragraph has methodological relevance as well, because intraindividual changes in cytokines and immunoglobulins during the 4 years were similar between participants who in 2020–21 were SARS-CoV-2 seropositive and seronegative, and between participants who did and did not develop COVID-19 disease. The similarity indicates that it is valid to use prepandemic levels of cytokines and immunoglobulins to assess their risk relationship (protective or harmful) with the development of SARS-CoV-2 seropositivity and COVID-19 disease (28). The longitudinal time sequence is unequivocal (2016–17 vs. 2020–21). By contrast, the cross-sectional analysis of the possible association between levels of cytokines and immunoglobulins in 2020–21 and SARS-CoV-2 seropositivity and COVID-19 disease (obviously, also in 2020–21) would not allow to analyze that relationship, because the putative cause and the effect were measured very near in time.

We did not observe many differences in cytokine concentrations by main sociodemographic groups; essentially, higher concentrations of some cytokines (G-CSF, TNF-*α*) in younger participants, and higher concentrations of Il-2 in women.

More differences were apparent for immunoglobulins: higher levels in younger participants of total IgG, total IgM, and IgMs and IgAs against CMV, EBV, and HCoV, and higher levels in women than men of total IgG, total IgM and all eight IgMs against CMV, EBV, and HCoV. Levels of IgMs were higher in normal weight participants than in overweight or obese participants, and lower in former smokers. Levels of IgG CMV pp150 were lower in participants with higher education, as previously observed (8, 29). Differences in immunological markers are expected by age, sex, lifestyle, and living conditions (3–7, 10, 29, 49, 50). One possible explanation for the decrease in the concentration of IgMs with age can be found in the Tagonski’s work, which states that there is a reduction of new antibodies in the aging process of the immune system due to a decreased proliferative capacity of the B cells (51). In relation to women having higher IgMs than men (6, 7), evidence suggests that women have stronger humoral and T-cell immune responses than men (52). Our results on concentrations of cytokines and immunoglobulins by sociodemographic factors in a general Western population provide novel information, with potential uses in clinical practice and research; e.g., as reference values for population subgroups defined by sex, age, education, BMI, and smoking (1, 6–8).

Changes observed in the concentrations of cytokines during the study period were of higher magnitude than for immunoglobulins. This was in part expected due to the increase of cytokines when infectious / inflammatory processes occur (1, 3, 9), whereas total antibody isotypes and subclasses are comprised by polyclonal antibodies against numerous different antigens / pathogens and specific exposures are diluted. Also, antibodies against CMV or EBV would increase only in case of first infections that may have happened during childhood or early adulthood, or reactivations, which should not occur often in healthy populations. Of particular interest is the stability of antibodies against coronaviruses of common cold, despite regular exposure; the stability suggests that they induce short-lived antibody responses (53).

The choice to Log10 transform cytokine concentrations before calculating the percent relative change underlies the primary finding that cytokine concentrations are stable. Small perturbations in cytokine levels of less than 10-fold (e.g., 2–5 times) may have a substantial effect on the immune response during disease and vaccination. Some previous studies took the Log10 transformation after assessing the fold-change, not before (20, 21). The two main reasons why results on the relative change are based on log-transformed data are: First, the relative changes with original concentrations could sometimes be large; e.g., in the interquartile ranges, six times or more with respect to the baseline concentration. As the Mann–Whitney’s *U* test, also known as the Wilcoxon Rank Sum test, was practically identical when we used the original concentrations and when we used log-transformed concentrations, we preferred to give tables with more homogeneous values. And second, having a more homogeneous scale of relative change in the percentages, with a same cut-off point (i.e., at less the 15% of increase or decrease in the relative change) for all cytokines and all immunoglobulins, the reader can get a quick idea of whether a specific cytokine or immunoglobulin has varied more over time compared to the 2016–17 values. The log-transformed concentrations are only for presentation purposes. The relevant results are that intra-individual variations in cytokine and immunoglobulin levels in *unvaccinated* citizens are similar between those who were infected with SARS and those who were not, and between those who developed COVID-19 and those who did not.

Some limitations and strengths of the study have been previously discussed (35) and are just summarized here. Because the amount of information and results that we report is already quite high, given the size of the study population, we did not report other possible predictors of the change in cytokines and immunoglobulins, such as seasonality, temperature, other infections, or other immunological and genetic parameters. In section 2.7 above we inform that no associations among prepandemic comorbidities, and cytokines or immunoglobulins, were observed; only some modest negative relationships were observed between IgMs and BMI, and between IgMs, some total IgGs and dyslipidemia (Supplementary Figure 4b).

The generalizability of the findings may be somewhat limited by some characteristics of the cohort, even if this is a cohort from the general population of Barcelona (i.e., different from cohorts based on hospitals or other health care facilities); naturally, a Western population is not representative of other populations, which have other environmental and social pressures, from endemic infectious diseases to pollution to poverty. Future replication in larger and diverse populations is necessary. Also, further studies focused on how environmental exposures (e.g., nutritional status, other contaminants) may influence longitudinal immune biomarker dynamics will be necessary, stimulated by the present findings. Some of the strengths of the present study are assay reproducibility, the use of optimized standard assay protocols, and timepoint consistency; they support the robustness and validity of our conclusions.

The study size, statistical power and precision were often low; yet, numerous estimates were precise. On the other hand, the imputation of concentrations in the less detected and quantified cytokines (e.g., MIG, IFN-*γ*, and IL-17) may overestimate their intraindividual change. We analyzed 30 cytokines, 24 isotype-antigen combination immunoglobulins, and 7 total immunoglobulins, a relatively large amount in itself, although common in the clinical literature. We could thus perform a considerable number of comparisons and, since ours is the first study of its kind (assessing intraindividual change in a general, non-institutionalized population), it is only logical that we assessed comprehensively such change and the influence of SARS-CoV-2 infection, COVID-19, and sociodemographic factors. These features of the study may generate false positives, and replication of our findings in larger population-based, longitudinal studies is required; but they have also strengths, since the number of potentially relevant cytokines and immunoglobulins is high. The comparisons (e.g., in intraindividual changes in concentrations across cytokines by COVID-19 disease status) could not generally be based on *a priori* clinical knowledge, because virtually none exists for such changes.

## 5 Conclusion

We provide novel information on physiological, basal ex-vivo concentrations of cytokines and immunoglobulins in a general population, which should be relevant for clinical practice and public health. Intraindividual changes in cytokines and immunoglobulins during the 4 years from 2016–17 to 2020–21 were moderate, and they did not differ between participants who in 2020–21 were SARS-CoV-2 seropositive and seronegative, nor between participants who did and did not develop COVID-19 disease. These findings are also novel and relevant for medicine and public health. In particular, the stability in the biomarkers is relevant to assess the role of the immunological and inflammatory state (measured through baseline levels of cytokines and immunoglobulins) in the development of SARS-CoV-2 seropositivity and COVID-19 disease, as well as in the susceptibility to other infections and pathologies. Immune biomarker stability might contribute to personalized preventive strategies, informing on patient vaccine or treatment responsiveness, or serve for patient monitoring in infectious disease contexts.

## Data Availability

The raw data supporting the conclusions of this article may be made available by the authors upon reasonable request, without undue reservation.

## Abbreviations

BHS: Barcelona Health Survey
BMI: body mass index
CI: confidence interval
CMV: cytomegalovirus
COVID-19: coronavirus disease 2019
EBV: Epstein–Barr virus
EGF: epidermal growth factor
FGF: fibroblast growth factor
G-CSF: granulocyte colony-stimulating factor
GM-CSF: granulocyte-macrophage colony-stimulating factor
HCoV: human common cold coronavirus
HGF: hepatocyte growth factor
IFN: interferon
Igs: immunoglobulins
IL: interleukin
IP-10: IFN-*γ* induced protein
LOQ: limit of quantification
MCP-1: monocyte chemoattractant protein
MFI: median fluorescent intensity
MIG: monokine induced by IFN-γ
MIP: macrophage inflammatory protein
OR: odds ratio
RANTES: regulated on activation normal T cell expressed and secreted
SARS-CoV-2: severe acute respiratory syndrome coronavirus 2
TNF: tumor necrosis factor
VEGF: vascular endothelial growth factor.
