# Supplementary material for: Stability of cytokine and immunoglobulin concentrations in the general population: prepandemic basal concentrations and intraindividual changes until the COVID-19 pandemic
Source: Front Public Health. 2025 Jul 2;13:1548379. doi: 10.3389/fpubh.2025.1548379 (PMC12263939; doi:10.3389/fpubh.2025.1548379)
Supplement: Supplementary file 1 [file Data_Sheet_1.docx]

**Supplementary material**

**General Index**

***A. Supplemental Tables***

**Supplemental Table 1.** Limits of quantification, and percentage of participants with concentrations between the lower and the upper limits of quantification.

**Supplemental Table 2.** Correlations between cytokines and IgM antibodies against CMV, EBV and HCoV in 2016-17.

**Supplemental Table 3.** Percentage of participants with relative intraindividual changes of cytokine concentrations from 2016-17 to 2020-21 ≥15% by SARS-CoV-2 seropositivity, and by COVID-19 disease.

**Supplemental Table 4.** Concentrations of 30 cytokines in 2016-17 by sex (N=240).

**Supplemental Table 5a.** Concentrations of 30 cytokines in 2016-17 by age group (N=240).

**Supplemental Table 5b.** Relative intraindividual change (%) from 2016-17 to 2020-21 of concentrations of cytokines, by age group (N=154).

**Supplemental Table 6a.** Concentrations of 30 cytokines in 2016-17 by body mass index (N=240).

**Supplemental Table 6b.** Relative intraindividual change (%) from 2016-17 to 2020-21 of concentrations of cytokines, by body mass index (N=154).

**Supplemental Table 7a.** Concentrations of 30 cytokines in 2016-17 by tobacco smoking (N=240).

**Supplemental Table 7b.** Relative intraindividual change (%) from 2016-17 to 2020-21 of concentrations of cytokines, by tobacco smoking (N=154).

**Supplemental Table 8a.** Concentrations of 30 cytokines in 2016-17 by educational level (N=240).

**Supplemental Table 8b.** Relative intraindividual change (%) from 2016-17 to 2020-21 of concentrations of cytokines, by educational level (N=154).

**Supplemental Table 9a.** Concentrations of 24 isotype-antigen combinations for cytomegalovirus, Epstein-Barr and common cold infections, and of total Igs in 2016-17 by sex (N=240).

**Supplemental Table 9b.** Relative intraindividual change (%) from 2016-17 to 2020-21 of concentrations of 24 isotype-antigen combinations for cytomegalovirus, Epstein-Barr and common cold infections, and of total Igs, by sex (N=154).

**Supplemental Table 10a.** Concentrations of 24 isotype-antigen combinations for cytomegalovirus, Epstein-Barr and common cold infections, and of total Igs in 2016-17 by age group (N=240).

**Supplemental Table 10b.** Relative intraindividual change (%) from 2016-17 to 2020-21 of concentrations of 24 isotype-antigen combinations for cytomegalovirus, Epstein-Barr and common cold infections, and of total Igs, by age group (N=154).

**Supplemental Table 11a.** Concentrations of 24 isotype-antigen combinations for cytomegalovirus, Epstein-Barr and common cold infections, and of total Igs in 2016-17 by body mass index (N=240).

**Supplemental Table 11b.** Relative intraindividual change (%) from 2016-17 to 2020-21 of concentrations of 24 isotype-antigen combinations for cytomegalovirus, Epstein-Barr and common cold infections, and of total Igs, by body mass index (N=154).

**Supplemental Table 12a.** Concentrations of 24 isotype-antigen combinations for cytomegalovirus, Epstein-Barr and common cold infections, and of total Igs in 2016-17 by tobacco smoking (N=240).

**Supplemental Table 12b.** Relative intraindividual change (%) from 2016-17 to 2020-21 of concentrations of 24 isotype-antigen combinations for cytomegalovirus, Epstein-Barr and common cold infections, and of total Igs, by tobacco smoking (N=154).

**Supplemental Table 13.** Relative intraindividual change (%) from 2016-17 to 2020-21 of concentrations of 24 isotype-antigen combinations for cytomegalovirus, Epstein-Barr and common cold infections, and of total Igs, by presence or absence of dyslipidemia (N=154).

**Supplemental Table 14a.** Concentrations of 24 isotype-antigen combinations for cytomegalovirus, Epstein-Barr and common cold infections, and of total Igs in 2016-17 by educational level (N=240).

**Supplemental Table 14b.** Relative intraindividual change (%) from 2016-17 to 2020-21 of concentrations of 24 isotype-antigen combinations for cytomegalovirus, Epstein-Barr and common cold infections, and of total Igs, by educational level (N=154).

***B. Supplemental Figures***

**Supplemental Figure 1**. Correlations between concentrations of cytokines and immunoglobulins in 2016-2017.

**Supplemental Figure 2**. Correlations between concentrations of cytokines in 2016-2017.

**Supplemental Figure 3**. Correlations between concentrations of immunoglobulins against CMV, EBV and HCoV, and total immunoglobulins in 2016-2017.

**Supplemental Figure 4a.** Correlations between concentrations of cytokines and comorbidities in 2016-2017.

**Supplemental Figure 4b.** Correlations between concentrations of immunoglobulins against CMV, EBV and HCoV, total immunoglobulins and comorbidities in 2016-2017.

**Supplemental Figure 5.** Concentrations of IL-8, IL-2R, IL-6 and TNF-α in 2020-21 in the 20 participants who developed COVID-19 (panel A); and relative intraindividual change of IL-8, IL-2R, IL-6 and TNF-α concentrations in 2020-21 with respect to concentrations in 2016-17 in the 20 participants who developed COVID-19 (panel B).

**Index of Tables and Figures, including supplemental material.**

By order of appearance in the text.

*Methods*

**Supplemental Table 1.** Limits of quantification, and percentage of participants with concentrations between the lower and the upper limits of quantification.

**Figure 1.** Percentage of quantification of 30 cytokines in 2016-17 and 2020-21.

**Supplemental Figure 1.** Correlations between concentrations of cytokines and immunoglobulins in 2016-17.

**Supplemental Table 2.** Correlations between cytokines and IgM antibodies against CMV, EBV and HCoV in 2016-17.

**Supplemental Figure 2.** Correlations between concentrations of cytokines in 2016-17.

**Supplemental Figure 3.** Correlations between concentrations of immunoglobulins against CMV, EBV and HCoV, and total immunoglobulins in 2016-17.

**Supplemental Figure 4a.** Correlations between concentrations of cytokines and comorbidities in 2016-2017.

**Supplemental Figure 4b.** Correlations between concentrations of immunoglobulins against CMV, EBV and HCoV, total immunoglobulins and comorbidities in 2016-2017.

*Results*

*Cytokines*

**Table 1.** Concentrations of 30 cytokines in 2016-17 and 2020-21. Absolute intraindividual change and relative intraindividual change (N=154).

**Table 2.** Relative intraindividual change (%) of cytokine concentrations from 2016-17 to 2020-21 in participants SARS-CoV-2 seronegative and seropositive, and in participants without COVID-19 disease and with COVID-19 disease.

**Figure 2.** Scatterplots of concentrations (log10, pg/mL) of cytokines in 2020-21 against concentrations in 2016-17 by SARS-CoV-2 seropositivity.

**Supplemental Figure 5.** Concentrations of IL-8, IL-2R, IL-6 and TNF-α in 2020-21 in the 20 participants who developed COVID-19 (panel A); and relative intraindividual change of IL-8, IL-2R, IL-6 and TNF-α concentrations in 2020-21 with respect to concentrations in 2016-17 in the 20 participants who developed COVID-19 (panel B).

**Supplemental Table 3.** Percentage of participants with relative intraindividual changes of cytokine concentrations from 2016-17 to 2020-21 ≥15% by SARS-CoV-2 seropositivity, and by COVID-19 disease.

**Table 3.** Relative intraindividual change (%) from 2016-17 to 2020-21 of concentrations of cytokines, by sex (N=154). Cytokines quantified in more than 70% of participants.

**Supplemental Table 4.** Concentrations of 30 cytokines in 2016-17 by sex (N=240).

**Supplemental Table 5a.** Concentrations of 30 cytokines in 2016-17 by age group (N=240).

**Supplemental Table 5b.** Relative intraindividual change (%) from 2016-17 to 2020-21 of concentrations of cytokines, by age group (N=154).

**Supplemental Table 6a.** Concentrations of 30 cytokines in 2016-17 by body mass index (N=240).

**Supplemental Table 6b.** Relative intraindividual change (%) from 2016-17 to 2020-21 of concentrations of cytokines, by body mass index (N=154).

**Supplemental Table 7a.** Concentrations of 30 cytokines in 2016-17 by tobacco smoking (N=240).

**Supplemental Table 7b.** Relative intraindividual change (%) from 2016-17 to 2020-21 of concentrations of cytokines, by tobacco smoking (N=154).

**Supplemental Table 8a.** Concentrations of 30 cytokines in 2016-17 by educational level (N=240).

**Supplemental Table 8b.** Relative intraindividual change (%) from 2016-17 to 2020-21of concentrations of cytokines, by educational level (N=154).

*Immunoglobulins*

**Table 4.** Concentrations of 24 isotype-antigen combinations for cytomegalovirus, Epstein-Barr, and common cold infections, and total Igs from 2016-17 to 2020-21. Absolute intraindividual change and relative intraindividual change (N=154).

**Table 5.** Relative intraindividual change (%) of 24 isotype-antigen combinations for cytomegalovirus, Epstein-Barr, and common cold infections, and total Igs concentrations from 2016-17 to 2020-21 in participants SARS-CoV-2 seronegative and seropositive, and in participants without COVID-19 disease and with COVID-19 disease.

**Figure 3.** Scatterplots of concentrations (log10, MFI) of immunoglobulins in 2020-21 against concentrations in 2016-17 by SARS-CoV-2 seropositivity.

**Supplemental Table 9a.** Concentrations of 24 isotype-antigen combinations for cytomegalovirus, Epstein-Barr and common cold infections, and of total Igs in 2016-17 by sex (N=240).

**Supplemental Table 9b.** Relative intraindividual change (%) from 2016-17 to 2020-21 of concentrations of 24 isotype-antigen combinations for cytomegalovirus, Epstein-Barr and common cold infections, and of total Igs, by sex (N=154).

**Supplemental Table 10a.** Concentrations of 24 isotype-antigen combinations for cytomegalovirus, Epstein-Barr and common cold infections, and of total Igs in 2016-17 by age group (N=240).

**Supplemental Table 10b.** Relative intraindividual change (%) from 2016-17 to 2020-21 of concentrations of 24 isotype-antigen combinations for cytomegalovirus, Epstein-Barr and common cold infections, and of total Igs, by age group (N=154).

**Supplemental Table 11a.** Concentrations of 24 isotype-antigen combinations for cytomegalovirus, Epstein-Barr and common cold infections, and of total Igs in 2016-17 by body mass index (N=240).

**Supplemental Table 11b.** Relative intraindividual change (%) from 2016-17 to 2020-21 of concentrations of 24 isotype-antigen combinations for cytomegalovirus, Epstein-Barr and common cold infections, and of total Igs, by body mass index (N=154).

**Supplemental Table 12a.** Concentrations of 24 isotype-antigen combinations for cytomegalovirus, Epstein-Barr and common cold infections, and of total Igs in 2016-17 by tobacco smoking (N=240).

**Supplemental Table 12b.** Relative intraindividual change (%) from 2016-17 to 2020-21 of concentrations of 24 isotype-antigen combinations for cytomegalovirus, Epstein-Barr and common cold infections, and of total Igs, by tobacco smoking (N=154).

**Supplemental Table 13.** Relative intraindividual change (%) from 2016-17 to 2020-21 of concentrations of 24 isotype-antigen combinations for cytomegalovirus, Epstein-Barr and common cold infections, and of total Igs, by dyslipidemia (N=154).

**Supplemental Table 14a.** Concentrations of 24 isotype-antigen combinations for cytomegalovirus, Epstein-Barr and common cold infections, and of total Igs in 2016-17 by educational level (N=240).

**Supplemental Table 14b.** Relative intraindividual change (%) from 2016-17 to 2020-21 of concentrations of 24 isotype-antigen combinations for cytomegalovirus, Epstein-Barr and common cold infections, and of total Igs, by educational level (N=154).

**Supplemental Table 1.** Limits of quantification, and percentage of participants with concentrations between the lower and the upper limits of quantification.

|  | **Period 2016-17**  (N = 240) | |  | **Period 2020-21**  (N = 174) | |  |
| --- | --- | --- | --- | --- | --- | --- |
| **Cytokine** | **Lower LOQ**  (range) | **Upper LOQ**  (range) | **Quantification (%)** | **Lower LOQ**  (range) | **Upper LOQ**  (range) | **Quantification (%)** |
| **Growth factors** |  |  |  |  |  |  |
| G-CSF | 5.30 – 16.18 | 26117 – 40458 | 76.3 | 5.30 – 12.57 | 26117 – 40458 | 77.6 |
| EGF | 3.01 – 9.48 | 4120 – 5465 | 94.2 | 3.95 – 9.48 | 4120 – 5465 | 98.3 |
| FGF | 2.10 – 3.27 | 3155^a^ | 87.1 | 2.10 – 3.27 | 3155^a^ | 86.2 |
| GM-CSF | 0.43 – 2.19 | 6740 – 7103 | 70.8 | 0.43 – 2.19 | 6740 – 7103 | 75.3 |
| HGF | 3.35 – 11.18 | 13001 – 13550 | 100.0 | 5.04 – 11.18 | 13001 – 13550 | 100.0 |
| VEGF | 0.07 – 0.10 | 1103 – 1634 | 99.6 | 0.07 – 0.10 | 1103 – 1634 | 99.4 |
|  |  |  |  |  |  |  |
| **Chemokines** |  |  |  |  |  |  |
| IL-8 | 0.26 – 0.82 | 5205 – 5790 | 100.0 | 0.34 – 0.82 | 5205 – 5790 | 100.0 |
| IP-10 | 0.14 – 0.46 | 981 – 1829 | 100.0 | 0.14 – 0.25 | 981 – 1829 | 90.2 |
| RANTES | 5.80 – 13.38 | 2985 – 7633 | 80.4 | 6.96 – 13.38 | 2985 – 7633 | 78.2 |
| EOTAXIN | 0.45 – 1.39 | 1237 – 1721 | 100.0 | 0.45 – 1.12 | 1237 – 1721 | 100.0 |
| MIP-1α | 9.37 – 15.29 | 13529 – 14115 | 66.3 | 9.37 – 15.29 | 13529 – 14115 | 68.4 |
| MIP-1β | 1.60 – 4.15 | 12184 – 13057 | 98.8 | 1.87 – 4.15 | 12184 – 12672 | 98.9 |
| MCP-1 | 4.04 – 11.49 | 16148 – 18550 | 99.6 | 4.04 – 11.35 | 16783 – 18550 | 99.4 |
| MIG | 6.26 – 12.54 | 3285 – 3592 | 41.3 | 6.26 – 9.70 | 3285 – 3592 | 36.8 |
|  |  |  |  |  |  |  |
| **TH1** |  |  |  |  |  |  |
| IL-2 | 0.53 – 0.98 | 12958 – 13778 | 88.3 | 0.53 – 0.98 | 13024 – 13778 | 93.7 |
| IL-12 | 0.66 – 2.34 | 5649 – 6880 | 100.0 | 0.66 – 2.34 | 5649 – 6880 | 100.0 |
| IFN-γ | 0.22 – 0.45 | 1638 – 1764 | 14.2 | 0.28 – 0.45 | 1638 – 1764 | 13.8 |
|  |  |  |  |  |  |  |
| **TH2** |  |  |  |  |  |  |
| IL-4 | 1.35 – 3.74 | 29732 – 34733 | 36.3 | 1.99 – 3.74 | 29732 – 32423 | 33.9 |
| IL-5 | 0.60 – 1.11 | 2802 – 3937 | 48.8 | 0.60 – 1.11 | 2802 – 3937 | 47.7 |
| IL-13 | 1.40 – 4.30 | 7930 – 9986 | 67.5 | 1.40 – 3.54 | 7930 – 9986 | 65.5 |
|  |  |  |  |  |  |  |
| **Pro-inflammatory** |  |  |  |  |  |  |
| IL-1β | 0.32 – 0.55 | 1909 – 2676 | 73.8 | 0.32 – 0.55 | 1909 – 2676 | 79.9 |
| TNF-α | 0.47 – 0.89 | 4313 – 4615 | 80.0 | 0.47 – 0.89 | 4313 – 4615 | 85.6 |
| IL-6 | 0.70 – 1.87 | 7800 – 9425 | 93.3 | 0.70 – 1.87 | 7800 – 9425 | 93.7 |
| IFN-α | 2.27 – 5.51 | 7350 – 8270 | 90.0 | 2.27 – 3.30 | 7390 – 8270 | 93.7 |
| IL-2R | 2.07 – 5.03 | 16856 – 17210 | 99.6 | 2.07 – 5.03 | 16856 – 17210 | 99.4 |
| IL-17 | 0.89 – 1.22 | 16006 – 17321 | 34.2 | 0.89 – 1.22 | 16006 – 17321 | 43.1 |
|  |  |  |  |  |  |  |
| **Regulatory** |  |  |  |  |  |  |
| IL-7 | 2.80 – 5.69 | 9585 – 12205 | 71.3 | 2.80 – 5.69 | 9585 – 12205 | 75.3 |
|  |  |  |  |  |  |  |
| **Anti-inflammatory** |  |  |  |  |  |  |
| IL-10 | 0.55 – 1.06 | 11047 – 11810 | 59.6 | 0.55 – 1.06 | 11047 – 11689 | 63.2 |
| IL-15 | 3.45 – 6.68 | 17496 – 18539 | 28.7 | 3.45 – 6.68 | 17496 – 18539 | 37.9 |
| IL-1RA | 3.94 – 10.77 | 68762 – 77553 | 99.2 | 3.94 – 10.77 | 68871 – 77553 | 100.0 |
|  |  |  |  |  |  |  |

LOQ: limit of quantification (pg/mL), see Methods section *2.3. Quantification of cytokines, chemokines and growth factors*

^a^ Same upper limit of quantification in all plates in the laboratory analyses.

Participants of the 2020-21 period were the 174 participants (out of the 240 participants of the baseline period), who attended the follow-up visit (i.e. all 174 participants are part of the group of 240), see Methods section *2.1. Study population*

**Supplemental Table 2.** Correlations between cytokines and IgM antibodies against CMV, EBV and HCoV in 2016-17.

|  | | **G-CSF** | **EGF** | **FGF** | **GM-CSF** | **IL-8** | **EOTAXIN** | **MIP-1α** | **MIP-1β** | **IL-2** | **IL-12** | **IL-4** | **IL-13** | **TNF-α** | **IL-6** | **IFN-α** | **IL-2R** | **IL-17** | **IL-7** | **IL-10** | **IL-15** | **IL-1RA** |
| --- | --- | --- | --- | --- | --- | --- | --- | --- | --- | --- | --- | --- | --- | --- | --- | --- | --- | --- | --- | --- | --- | --- |
|  |  |  |  |  |  |  |  |  |  |  |  |  |  |  |  |  |  |  |  |  |  |  |
| **IgM_CMV_pp150** | ρ | **0.249** | **0.300** | **0.239** | **0.259** | **-0.199** | **-0.245** | **0.286** | **0.323** | **0.374** | **0.174** | 0.036 | 0.112 | **0.215** | **0.186** | **0.341** | **0.230** | **0.133** | 0.113 | **0.309** | **0.150** | **0.243** |
|  | p value | <0.001 | <0.001 | <0.001 | <0.001 | 0.002 | <0.001 | <0.001 | <0.001 | <0.001 | 0.007 | 0.582 | 0.085 | 0.001 | 0.004 | <0.001 | <0.001 | 0.039 | 0.080 | <0.001 | 0.020 | <0.001 |
|  |  |  |  |  |  |  |  |  |  |  |  |  |  |  |  |  |  |  |  |  |  |  |
| **IgM_CMV_pp65** | ρ | **0.181** | **0.250** | **0.149** | **0.177** | **-0.248** | **-0.226** | 0.107 | **0.276** | **0.298** | -0.018 | 0.050 | 0.071 | **0.236** | 0.069 | **0.228** | **0.183** | 0.087 | -0.012 | **0.189** | 0.110 | **0.195** |
|  | p value | 0.005 | <0.001 | 0.021 | 0.006 | <0.001 | <0.001 | 0.097 | <0.001 | <0.001 | 0.776 | 0.441 | 0.274 | <0.001 | 0.288 | <0.001 | 0.004 | 0.180 | 0.853 | 0.003 | 0.090 | 0.002 |
|  |  |  |  |  |  |  |  |  |  |  |  |  |  |  |  |  |  |  |  |  |  |  |
| **IgM_EBV_EAD** | ρ | **0.210** | **0.329** | **0.213** | **0.220** | **-0.235** | **-0.161** | **0.147** | **0.340** | **0.355** | 0.101 | 0.047 | **0.147** | **0.254** | **0.166** | **0.335** | **0.235** | **0.197** | 0.062 | **0.233** | **0.146** | **0.231** |
|  | p value | 0.001 | <0.001 | 0.001 | 0.001 | <0.001 | 0.013 | 0.023 | <0.001 | <0.001 | 0.120 | 0.468 | 0.023 | <0.001 | 0.010 | <0.001 | <0.001 | 0.002 | 0.337 | <0.001 | 0.023 | <0.001 |
|  |  |  |  |  |  |  |  |  |  |  |  |  |  |  |  |  |  |  |  |  |  |  |
| **lgM_EBV_VCAp18** | ρ | **0.142** | **0.247** | **0.135** | **0.150** | **-0.239** | **-0.200** | **0.128** | **0.258** | **0.282** | 0.119 | 0.095 | 0.076 | **0.217** | 0.061 | **0.221** | **0.152** | 0.096 | -0.015 | **0.158** | 0.106 | **0.249** |
|  | p value | 0.028 | <0.001 | 0.037 | 0.020 | <0.001 | 0.002 | 0.048 | <0.001 | <0.001 | 0.066 | 0.143 | 0.241 | 0.001 | 0.347 | 0.001 | 0.019 | 0.137 | 0.817 | 0.014 | 0.101 | <0.001 |
|  |  |  |  |  |  |  |  |  |  |  |  |  |  |  |  |  |  |  |  |  |  |  |
| **IgM_N_229E** | ρ | **0.314** | **0.319** | **0.234** | **0.292** | **-0.181** | **-0.160** | **0.189** | **0.311** | **0.353** | **0.135** | 0.044 | **0.229** | **0.341** | **0.239** | **0.310** | **0.247** | **0.166** | **0.141** | **0.247** | 0.113 | **0.298** |
|  | p value | <0.001 | <0.001 | <0.001 | <0.001 | 0.005 | 0.013 | 0.003 | <0.001 | <0.001 | 0.037 | 0.500 | <0.001 | <0.001 | <0.001 | <0.001 | <0.001 | 0.010 | 0.029 | <0.001 | 0.079 | <0.001 |
|  |  |  |  |  |  |  |  |  |  |  |  |  |  |  |  |  |  |  |  |  |  |  |
| **IgM_N_HKU1** | ρ | **0.240** | **0.301** | **0.186** | **0.266** | **-0.184** | **-0.198** | **0.173** | **0.286** | **0.340** | 0.112 | 0.108 | **0.155** | **0.246** | **0.158** | **0.285** | **0.222** | **0.141** | 0.082 | **0.289** | **0.131** | **0.216** |
|  | p value | <0.001 | <0.001 | 0.004 | <0.001 | 0.004 | 0.002 | 0.007 | <0.001 | <0.001 | 0.084 | 0.096 | 0.017 | <0.001 | 0.014 | <0.001 | 0.001 | 0.029 | 0.204 | <0.001 | 0.043 | 0.001 |
|  |  |  |  |  |  |  |  |  |  |  |  |  |  |  |  |  |  |  |  |  |  |  |
| **IgM_N_NL63** | ρ | **0.207** | **0.241** | **0.151** | **0.159** | **-0.196** | **-0.226** | **0.127** | **0.242** | **0.251** | 0.110 | 0.068 | 0.073 | **0.248** | **0.133** | **0.222** | **0.197** | 0.089 | 0.034 | **0.178** | 0.109 | **0.155** |
|  | p value | 0.001 | <0.001 | 0.019 | 0.014 | 0.002 | <0.001 | 0.050 | <0.001 | <0.001 | 0.088 | 0.296 | 0.259 | <0.001 | 0.040 | 0.001 | 0.002 | 0.169 | 0.600 | 0.006 | 0.093 | 0.016 |
|  |  |  |  |  |  |  |  |  |  |  |  |  |  |  |  |  |  |  |  |  |  |  |
| **IgM_N_OC43** | ρ | **0.226** | **0.325** | **0.148** | **0.230** | **-0.139** | **-0.106** | **0.137** | **0.313** | **0.318** | **0.198** | **0.167** | 0.120 | **0.287** | **0.195** | **0.294** | **0.248** | **0.178** | 0.045 | **0.253** | **0.180** | **0.296** |
|  | p value | <0.001 | <0.001 | 0.022 | <0.001 | 0.032 | 0.102 | 0.034 | <0.001 | <0.001 | 0.002 | 0.009 | 0.064 | <0.001 | 0.002 | <0.001 | <0.001 | 0.006 | 0.487 | <0.001 | 0.005 | <0.001 |
|  |  |  |  |  |  |  |  |  |  |  |  |  |  |  |  |  |  |  |  |  |  |  |

ρ: Spearman’s rho. Bold ρ: p<0.05.

Marked in orange: cytokines positively associated with all or virtually all IgM.

Marked in green: cytokines inversely associated with all or virtually all IgM.

Cytokines HGF, VEGF, IP-10, RANTES, MCP-1, MIG, IFN-γ, IL-5, and IL-1β are not shown because no ρ had a p<0.05.

**Supplemental Table 3.** Percentage of participants with different numbers of cytokines with a relative intraindividual change of concentrations from 2016-17 to 2020-21 equal or greater than 15%, by SARS-CoV-2 seropositivity, and by COVID-19 disease.

|  |  |  | **SARS-CoV-2 status** | |  |  | **COVID-19 disease** | |  |
| --- | --- | --- | --- | --- | --- | --- | --- | --- | --- |
| **Change in cytokine concentrations^a^** | **Total**  (N = 154) |  | **Seronegative**  (N = 104) | **Seropositive**  (N = 41) | **P** |  | **No COVID**  (N = 134) | **COVID**  (N = 20) | **P** |
|  | (%) |  | (%) | (%) |  |  | (%) | (%) |  |
| **Change ≥15%**  **(∆ ≥15% or ∇ ≥15%)** (median)^b^ | 12.0 |  | 12.0 | 12.0 | 0.940^c^ |  | 12.0 | 12.5 | 0.985^c^ |
| No change ≥15% | 0.0 |  | – | – |  |  | – | – |  |
| Change ≥15% in 1 to 10 CKs | 40.3 |  | 38.5 | 43.9 | 0.576^d^ |  | 39.6 | 45.0 | 0.635^d^ |
| Change ≥15% in >10 CKs | 59.7 |  | 61.5 | 56.1 |  |  | 60.4 | 55.0 |  |
|  |  |  |  |  |  |  |  |  |  |
| **∆ ≥15%** (median)^b^ | 6.0 |  | 6.0 | 6.0 | 0.682^c^ |  | 6.0 | 3.0 | 0.238^c^ |
| No ∆ ≥15% | 6.5 |  | 6.7 | 4.9 | >0.999^d^ |  | 6.7 | 5.0 | >0.999^d^ |
| ∆ ≥15% in 1 to 10 CKs | 68.2 |  | 67.3 | 68.3 |  |  | 67.9 | 70.0 |  |
| ∆ ≥15% in >10 CKs | 25.3 |  | 26.0 | 26.8 |  |  | 25.4 | 25.0 |  |
|  |  |  |  |  |  |  |  |  |  |
| **∇ ≥15%** (median)^b^ | 4.0 |  | 3.0 | 5.0 | 0.529^c^ |  | 3.5 | 6.5 | 0.104^c^ |
| No ∇≥15% | 3.2 |  | 1.0 | 9.8 | 0.031^d^ |  | 3.0 | 5.0 | 0.739^d^ |
| ∇ ≥15% in 1 to 10 CKs | 84.4 |  | 85.5 | 82.9 |  |  | 84.3 | 85.0 |  |
| ∇ ≥15% in >10 CKs | 12.3 |  | 13.5 | 7.3 |  |  | 12.7 | 10.0 |  |
|  |  |  |  |  |  |  |  |  |  |

^a^ Units used for computing the relative intraindividual change were base 10 logtransformed pg/mL.

^b^ Median number of cytokines per participant that meet the corresponding intraindividual change concentration.

^c^ Mann-Whitney’s *U* test (two-tail).

^d^ Fisher’s exact test (two-tail).

68% of participants had between 1 and 10 cytokines with a relative intraindividual increase of concentrations ≥15%. The maximum number of cytokines with an increase ≥15% in one person was 24, while 6.5% of participants had no cytokine with an increase ≥15%. The number of cytokines with an increase ≥15% was similar for seropositive and seronegative SARS-CoV-2 infection, and for participants with and without COVID-19 disease.

63% of participants had between 1 and 5 cytokines with a relative intraindividual decrease ≥15%. The maximum number of cytokines with a decrease ≥15% in one person was 27 (90% of 30 cytokines), and while 3.2% of participants had no cytokine with a decrease ≥15%. The number of cytokines with a decrease ≥15% for participant was higher for SARS-CoV-2 seropositives participants (vs. seronegatives), and for participants who developed COVID-19 disease (vs. participants without COVID-19).

**Supplemental Table 4.** Concentrations of 30 cytokines in 2016-17 by sex (N=240).

|  |  |  |  | |  |
| --- | --- | --- | --- | --- | --- |
| **Cytokine** (pg/mL) | **Total**  (N = 240) |  | **Men**  (N = 113) | **Women**  (N = 127) | **P^a^** |
| **Growth factors** |  |  |  |  |  |
| **G-CSF** (median) | 33.35 |  | 32.39 | 49.48 | 0.180 |
| (P25, P75) | (8.09, 108.0) |  | (6.29, 98.33) | (11.30, 125.5) |  |
| Geometric mean | 35.72 |  | 31.65 | 39.78 |  |
|  |  |  |  |  |  |
| **EGF** (median) | 85.75 |  | 72.05 | 88.83 | 0.062 |
| (P25, P75) | (41.3, 154.9) |  | (38.03, 119.7) | (47.89, 211.6) |  |
| Geometric mean | 80.02 |  | 64.91 | 96.41 |  |
|  |  |  |  |  |  |
| **FGF** (median) | 12.90 |  | 12.34 | 13.36 | 0.634 |
| (P25, P75) | (4.35, 33.03) |  | (4.60, 30.96) | (4.19, 33.42) |  |
| Geometric mean | 14.61 |  | 14.13 | 15.05 |  |
|  |  |  |  |  |  |
| **GM-CSF** (median) | 8.83 |  | 8.53 | 10.49 | 0.607 |
| (P25, P75) | (1.09, 46.36) |  | (1.09, 46.17) | (1.09, 47.64) |  |
| Geometric mean | 8.04 |  | 7.24 | 8.82 |  |
|  |  |  |  |  |  |
| **HGF** (median) | 367.4 |  | 371.3 | 363.6 | 0.249 |
| (P25, P75) | (262.6, 525.0) |  | (286.1, 582.6) | (246.0, 511.9) |  |
| Geometric mean | 381.4 |  | 404.7 | 361.7 |  |
|  |  |  |  |  |  |
| **VEGF** (median) | 5.37 |  | 4.54 | 5.46 | 0.978 |
| (P25, P75) | (2.36, 9.53) |  | (2.55, 9.87) | (2.13, 8.75) |  |
| Geometric mean | 4.53 |  | 4.66 | 4.42 |  |
| **Chemokines** |  |  |  |  |  |
| **IL-8** (median) | 16.25 |  | 17.44 | 15.25 | 0.109 |
| (P25, P75) | (12.16, 23.47) |  | (12.77, 24.68) | (11.17, 22.66) |  |
| Geometric mean | 16.13 |  | 17.15 | 15.28 |  |
|  |  |  |  |  |  |
| **IP-10** (median) | 6.68 |  | 6.71 | 6.52 | 0.810 |
| (P25, P75) | (4.18, 10.13) |  | (4.43, 9.80) | (3.98, 10.18) |  |
| Geometric mean | 6.73 |  | 6.84 | 6.63 |  |
|  |  |  |  |  |  |
| **RANTES** (median) | 3414 |  | 3455 | 3339 | 0.899 |
| (P25, P75) | (2808, 4212) |  | (2829, 3980) | (2733, 4760) |  |
| Geometric mean | 3603 |  | 3519 | 3679 |  |
|  |  |  |  |  |  |
| **EOTAXIN** (median) | 77.07 |  | 87.08 | 70.98 | 0.001 |
| (P25, P75) | (58.44, 100.9) |  | (63.62, 110.9) | (54.96, 95.66) |  |
| Geometric mean | 76.92 |  | 85.94 | 69.70 |  |
|  |  |  |  |  |  |
| **MIP-1α** (median) | 36.29 |  | 28.91 | 38.02 | 0.183 |
| (P25, P75) | (<LOQ, 113.6) |  | (<LOQ, 109.3) | (<LOQ, 118.1) |  |
| Geometric mean | 37.09 |  | 33.05 | 41.10 |  |
|  |  |  |  |  |  |
| **MIP-1β** (median) | 172.9 |  | 147.3 | 200.5 | 0.042 |
| (P25, P75) | (93.08, 446.9) |  | (85.39, 316.3) | (100.2, 578.1) |  |
| Geometric mean | 216.2 |  | 176.5 | 259.0 |  |
|  |  |  |  |  |  |
| **MCP-1** (median) | 654.4 |  | 672.0 | 624.5 | 0.159 |
| (P25, P75) | (493.9, 894.9) |  | (506.6, 969.8) | (488.1, 834.2) |  |
| Geometric mean | 707.8 |  | 736.6 | 683.1 |  |
|  |  |  |  |  |  |
| **MIG** (median) | <LOQ |  | <LOQ | <LOQ | – |
| (P25, P75) | (<LOQ, 26.85) |  | (<LOQ, 30.51) | <LOQ, 20.63) |  |
| Geometric mean | 10.52 |  | 11.03 | 10.09 |  |
|  |  |  |  |  |  |

[ continued ]

**Supplemental Table 4.** Continued.

|  |  |  |  | |  |
| --- | --- | --- | --- | --- | --- |
| **Cytokine** (pg/mL) | **Total** |  | **Men** | **Women** | **P^a^** |
| **TH1** |  |  |  |  |  |
| **IL-2** (median) | 12.60 |  | 9.13 | 18.87 | 0.028 |
| (P25, P75) | (2.46, 61.21) |  | (1.9, 39.10) | (3.63, 101.4) |  |
| Geometric mean | 14.13 |  | 9.79 | 19.60 |  |
|  |  |  |  |  |  |
| **IL-12** (median) | 57.61 |  | 51.66 | 61.49 | 0.078 |
| (P25, P75) | (40.98, 89.25) |  | (32.02, 82.86) | (45.36, 92.09) |  |
| Geometric mean | 60.24 |  | 55.81 | 64.47 |  |
|  |  |  |  |  |  |
| **IFN-γ** (median) | <LOQ |  | <LOQ | <LOQ | – |
| (P25, P75) | (<LOQ, 0.22) |  | (<LOQ, 0.22) | (<LOQ, 0.22) |  |
| Geometric mean | <LOQ |  | 0.22 | <LOQ |  |
| **TH2** |  |  |  |  |  |
| **IL-4** (median) | 1.54 |  | 1.54 | 1.54 | 0.461 |
| (P25, P75) | (1.42, 4.57) |  | (1.42, 4.70) | (1.42, 4.55) |  |
| Geometric mean | 3.46 |  | 3.57 | 3.37 |  |
|  |  |  |  |  |  |
| **IL-5** (median) | <LOQ |  | 0.77 | <LOQ | – |
| (P25, P75) | (<LOQ, 1.11) |  | (<LOQ, 1.11) | (<LOQ, 1.11) |  |
| Geometric mean | 0.84 |  | 0.93 | 0.78 |  |
|  |  |  |  |  |  |
| **IL-13** (median) | 6.90 |  | 5.48 | 8.35 | 0.227 |
| (P25, P75) | (<LOQ, 17.34) |  | (<LOQ, 17.83) | (1.77, 16.25) |  |
| Geometric mean | 7.12 |  | 6.38 | 7.84 |  |
| **Pro-inflammatory** |  |  |  |  |  |
| **IL-1β** (median) | 0.91 |  | 0.93 | 0.85 | 0.600 |
| (P25, P75) | (<LOQ, 2.00) |  | (<LOQ, 2.22) | (<LOQ, 1.80) |  |
| Geometric mean | 1.07 |  | 1.14 | 1.01 |  |
|  |  |  |  |  |  |
| **TNF-α** (median) | 3.28 |  | 2.80 | 4.97 | 0.131 |
| (P25, P75) | (1.34, 14.30) |  | (1.13, 12.22) | (1.39, 15.81) |  |
| Geometric mean | 4.02 |  | 3.34 | 4.73 |  |
|  |  |  |  |  |  |
| **IL-6** (median) | 15.74 |  | 16.37 | 15.59 | 0.242 |
| (P25, P75) | (4.71, 51.39) |  | (4.15, 41.15) | (5.54, 76.24) |  |
| Geometric mean | 16.29 |  | 14.08 | 18.56 |  |
|  |  |  |  |  |  |
| **IFN-α** (median) | 19.42 |  | 16.62 | 22.44 | 0.105 |
| (P25, P75) | (6.86, 54.94) |  | (5.89, 45.91) | (7.00, 70.70) |  |
| Geometric mean | 19.46 |  | 16.17 | 22.93 |  |
|  |  |  |  |  |  |
| **IL-2R** (median) | 141.6 |  | 127.3 | 156.8 | 0.185 |
| (P25, P75) | (55.03, 427.8) |  | (47.53, 420.6) | (57.75, 436.7) |  |
| Geometric mean | 169.4 |  | 149.9 | 188.8 |  |
|  |  |  |  |  |  |
| **IL-17** (median) | <LOQ |  | <LOQ | <LOQ | – |
| (P25, P75) | (<LOQ, 1.56) |  | (<LOQ, 1.58) | (<LOQ, 1.54) |  |
| Geometric mean | 1.19 |  | 1.35 | 1.06 |  |
| **Regulatory** |  |  |  |  |  |
| **IL-7** (median) | 14.01 |  | 14.01 | 13.89 | 0.631 |
| (P25, P75) | (2.84, 31.06) |  | (2.84, 32.51) | (2.84, 30.34) |  |
| Geometric mean | 12.33 |  | 12.82 | 11.90 |  |
|  |  |  |  |  |  |

[ continued ]

**Supplemental Table 4.** Continued.

|  |  |  |  | |  |
| --- | --- | --- | --- | --- | --- |
| **Cytokine** (pg/mL) | **Total** |  | **Men** | **Women** | **P^a^** |
| **Anti-inflammatory** |  |  |  |  |  |
| **IL-10** (median) | 3.34 |  | 3.02 | 4.18 | 0.179 |
| (P25, P75) | (<LOQ, 30.62) |  | (<LOQ, 23.89) | (<LOQ, 36.85) |  |
| Geometric mean | 4.46 |  | 3.77 | 5.18 |  |
|  |  |  |  |  |  |
| **IL-15** (median) | <LOQ |  | <LOQ | <LOQ | – |
| (P25, P75) | (<LOQ, 42.13) |  | (<LOQ, 9.90) | (<LOQ, 63.66) |  |
| Geometric mean | 9.28 |  | 7.90 | 10.70 |  |
|  |  |  |  |  |  |
| **IL-1RA** (median) | 262.5 |  | 228.4 | 288.4 | 0.012 |
| (P25, P75) | (166.7, 504.7) |  | (143.5, 390.6) | (182.4, 576.8) |  |
| Geometric mean | 295.7 |  | 257.8 | 334.2 |  |
|  |  |  |  |  |  |

P25: percentile 25th. P75: percentile 75th.

<LOQ: value lower than the minimum concentration of the corresponding range of limits of quantification.

See Supplemental Table 1.

^a^ Mann-Whitney’s *U* test (two-tail).

**Supplemental Table 5a.** Concentrations of 30 cytokines in 2016-17 by age group (N=240).

|  |  |  |  |  |
| --- | --- | --- | --- | --- |
| **Cytokine** (pg/mL) | **<45 years**  (N = 88) | **45 – 64 years**  (N = 105) | **≥65 years**  (N = 47) | **P^a^** |
| **Growth factors** |  |  |  |  |
| **G-CSF** (median) | 70.91 | 22.84 | 22.94 | 0.012 |
| (P25, P75) | (14.06, 158.9) | (<LOQ, 93.45) | (8.09, 92.29) |  |
| Geometric mean | 52.69 | 27.83 | 30.14 |  |
|  |  |  |  |  |
| **EGF** (median) | 92.48 | 76.33 | 75.12 | 0.400 |
| (P25, P75) | (52.16, 161.4) | (39.38, 195.9) | (38.41, 110.3) |  |
| Geometric mean | 81.04 | 78.98 | 80.48 |  |
|  |  |  |  |  |
| **FGF** (median) | 14.10 | 12.34 | 10.49 | 0.426 |
| (P25, P75) | (5.29, 36.18) | (4.35, 38.90) | (3.86, 22.02) |  |
| Geometric mean | 15.99 | 14.70 | 12.16 |  |
|  |  |  |  |  |
| **GM-CSF** (median) | 11.84 | 9.26 | 4.70 | 0.293 |
| (P25, P75) | (1.15, 51.59) | (0.74, 57.23) | (0.74, 32.54) |  |
| Geometric mean | 9.59 | 8.46 | 5.15 |  |
|  |  |  |  |  |
| **HGF** (median) | 344.9 | 389.7 | 375.9 | 0.060 |
| (P25, P75) | (222.2, 469.8) | (262.6, 654.2) | (310.1, 489.7) |  |
| Geometric mean | 333.0 | 401.6 | 437.9 |  |
|  |  |  |  |  |
| **VEGF** (median) | 4.49 | 5.46 | 5.78 | 0.541 |
| (P25, P75) | (2.08, 10.21) | (2.28, 9.06) | (3.06, 10.20) |  |
| Geometric mean | 4.43 | 4.16 | 5.71 |  |
| **Chemokines** |  |  |  |  |
| **IL-8** (median) | 14.40 | 15.14 | 22.10 | <0.001 |
| (P25, P75) | (10.63, 21.64) | (11.93, 22.38) | (16.32, 30.70) |  |
| Geometric mean | 14.95 | 15.28 | 21.02 |  |
|  |  |  |  |  |
| **IP-10** (median) | 6.29 | 5.92 | 8.50 | 0.006 |
| (P25, P75) | (4.23, 9.52) | (3.69, 9.64) | (6.26, 15.27) |  |
| Geometric mean | 6.21 | 6.26 | 9.17 |  |
|  |  |  |  |  |
| **RANTES** (median) | 3331 | 3369 | 3529 | 0.382 |
| (P25, P75) | (2821, 4095) | (2742, 4322) | (2992, 4760) |  |
| Geometric mean | 3497 | 3574 | 3877 |  |
|  |  |  |  |  |
| **EOTAXIN** (median) | 62.49 | 81.72 | 95.42 | <0.001 |
| (P25, P75) | (50.28, 86.62) | (62.12, 109.0) | (66.58, 134.7) |  |
| Geometric mean | 64.33 | 81.03 | 95.68 |  |
|  |  |  |  |  |
| **MIP-1α** (median) | 37.16 | 36.29 | 31.81 | 0.968 |
| (P25, P75) | (<LOQ, 108.6) | (<LOQ, 131.2) | (<LOQ, 95.02) |  |
| Geometric mean | 34.65 | 39.74 | 36.12 |  |
|  |  |  |  |  |
| **MIP-1β** (median) | 219.2 | 135.9 | 140.4 | 0.138 |
| (P25, P75) | (124.4, 458.0) | (82.22, 513.9) | (91.02, 268.9) |  |
| Geometric mean | 240.7 | 213.3 | 182.3 |  |
|  |  |  |  |  |
| **MCP-1** (median) | 583.0 | 677.1 | 750.8 | 0.023 |
| (P25, P75) | (471.2, 741.7) | (509.2, 930.9) | (541.1, 976.3) |  |
| Geometric mean | 628.5 | 743.4 | 792.3 |  |
|  |  |  |  |  |
| **MIG** (median) | <LOQ | <LOQ | 6.27 | – |
| (P25, P75) | (<LOQ, 20.63) | (<LOQ, 26.85) | (<LOQ, 44.62) |  |
| Geometric mean | 9.15 | 10.75 | 13.01 |  |
|  |  |  |  |  |

[ continued ]

**Supplemental Table 5a.** Continued.

|  |  |  |  |  |
| --- | --- | --- | --- | --- |
| **Cytokine** (pg/mL) | **<45 years**  (N = 88) | **45 – 64 years**  (N = 105) | **≥65 years**  (N = 47) | **P^a^** |
| **TH1** |  |  |  |  |
| **IL-2** (median) | 18.55 | 11.05 | 6.07 | 0.131 |
| (P25, P75) | (5.22, 77.37) | (1.90, 106.1) | (2.20, 32.26) |  |
| Geometric mean | 17.60 | 14.52 | 8.83 |  |
|  |  |  |  |  |
| **IL-12** (median) | 58.60 | 51.98 | 63.59 | 0.369 |
| (P25, P75) | (42.95, 81.19) | (33.82, 87.07) | (43.60, 108.7) |  |
| Geometric mean | 58.86 | 57.03 | 71.07 |  |
|  |  |  |  |  |
| **IFN-γ** (median) | <LOQ | <LOQ | <LOQ | – |
| (P25, P75) | (<LOQ, 0.22) | (<LOQ, 0.22) | (<LOQ, 0.22) |  |
| Geometric mean | <LOQ | <LOQ | <LOQ |  |
| **TH2** |  |  |  |  |
| **IL-4** (median) | 1.54 | 1.59 | 1.54 | 0.817 |
| (P25, P75) | (1.42, 4.41) | (1.42, 7.25) | (1.42, 3.94) |  |
| Geometric mean | 3.34 | 3.73 | 3.12 |  |
|  |  |  |  |  |
| **IL-5** (median) | <LOQ | 0.66 | 0.66 | – |
| (P25, P75) | (<LOQ, 1.11) | (<LOQ, 1.35) | (<LOQ, 0.91) |  |
| Geometric mean | 0.82 | 0.94 | 0.67 |  |
|  |  |  |  |  |
| **IL-13** (median) | 8.48 | 5.48 | 5.09 | 0.353 |
| (P25, P75) | (1.77, 20.22) | (<LOQ, 20.12) | (1.77, 11.66) |  |
| Geometric mean | 8.41 | 7.04 | 5.33 |  |
| **Pro-inflammatory** |  |  |  |  |
| **IL-1β** (median) | 0.88 | 0.85 | 0.99 | 0.667 |
| (P25, P75) | (<LOQ, 1.60) | (<LOQ, 2.29) | (0.52, 2.26) |  |
| Geometric mean | 0.99 | 1.07 | 1.23 |  |
|  |  |  |  |  |
| **TNF-α** (median) | 8.84 | 2.43 | 1.74 | 0.001 |
| (P25, P75) | (1.53, 25.83) | (0.44, 11.34) | (1.13, 6.84) |  |
| Geometric mean | 6.86 | 3.05 | 2.74 |  |
|  |  |  |  |  |
| **IL-6** (median) | 22.91 | 15.22 | 11.04 | 0.780 |
| (P25, P75) | (4.36, 70.10) | (4.44, 48.57) | (5.46, 41.55) |  |
| Geometric mean | 17.18 | 15.63 | 16.20 |  |
|  |  |  |  |  |
| **IFN-α** (median) | 23.30 | 17.65 | 15.11 | 0.379 |
| (P25, P75) | (8.01, 64.43) | (4.93, 64.82) | (5.78, 35.91) |  |
| Geometric mean | 21.29 | 19.58 | 16.19 |  |
|  |  |  |  |  |
| **IL-2R** (median) | 195.2 | 127.3 | 134.4 | 0.280 |
| (P25, P75) | (63.68, 574.6) | (47.41, 382.7) | (54.48, 397.1) |  |
| Geometric mean | 194.3 | 152.6 | 165.3 |  |
|  |  |  |  |  |
| **IL-17** (median) | <LOQ | <LOQ | <LOQ | – |
| (P25, P75) | (<LOQ, 1.54) | (<LOQ, 1.81) | (<LOQ, 1.59) |  |
| Geometric mean | 1.06 | 1.30 | 1.20 |  |
| **Regulatory** |  |  |  |  |
| **IL-7** (median) | 14.01 | 17.36 | 10.51 | 0.616 |
| (P25, P75) | (3.72, 32.51) | (<LOQ, 31.06) | (2.84, 28.63) |  |
| Geometric mean | 13.60 | 12.21 | 10.47 |  |
|  |  |  |  |  |

[ continued ]

**Supplemental Table 5a.** Continued.

|  |  |  |  |  |
| --- | --- | --- | --- | --- |
| **Cytokine** (pg/mL) | **<45 years**  (N = 88) | **45 – 64 years**  (N = 105) | **≥65 years**  (N = 47) | **P^a^** |
| **Anti-inflammatory** |  |  |  |  |
| **IL-10** (median) | 4.14 | 3.54 | 1.24 | 0.508 |
| (P25, P75) | (<LOQ, 36.49) | (<LOQ, 38.87) | (<LOQ, 14.33) |  |
| Geometric mean | 4.66 | 4.98 | 3.21 |  |
|  |  |  |  |  |
| **IL-15** (median) | <LOQ | <LOQ | <LOQ | – |
| (P25, P75) | (<LOQ, 29.08) | (<LOQ, 108.0) | (<LOQ, <LOQ) |  |
| Geometric mean | 8.46 | 10.97 | 7.57 |  |
|  |  |  |  |  |
| **IL-1RA** (median) | 344.2 | 255.0 | 206.5 | 0.105 |
| (P25, P75) | (177.0, 514.7) | (173.0, 519.7) | (150.2, 326.5) |  |
| Geometric mean | 295.3 | 311.6 | 263.9 |  |
|  |  |  |  |  |

P25: percentile 25th. P75: percentile 75th.

<LOQ: value lower than the minimum concentration of the corresponding range of limits of quantification.

See Supplemental Table 1.

^a^ Kruskal-Wallis test (two-tailed).

**Supplemental Table 5b.** Relative intraindividual change (%) from 2016-17 to 2020-21 of concentrations of cytokines, by age group (N=154).

|  |  | | |  |
| --- | --- | --- | --- | --- |
| **Cytokine^a^** | **<46 years**  (N = 51) | **46 – 62 years**  (N = 49) | **≥63 years**  (N = 54) | **P^b^** |
| **Growth factors** |  |  |  |  |
| **G-CSF** (median) | 6.77 | 0.00 | 0.00 | 0.175 |
| (P25, P75) | (-7.42, 25.67) | (0.00, 36.81) | (-5.62, 13.98) |  |
| ∆ ≥15% | 39.2 | 36.7 | 24.1 |  |
| ∇ ≥15% | 11.8 | 16.3 | 20.4 |  |
|  |  |  |  |  |
| **EGF** (median) | 9.29 | 5.47 | 3.85 | 0.038 |
| (P25, P75) | (1.84, 27.08) | (-5.13, 30.29) | (-3.83, 12.20) |  |
| ∆ ≥15% | 41.2 | 34.7 | 22.2 |  |
| ∇ ≥15% | 2.0 | 16.3 | 9.3 |  |
|  |  |  |  |  |
| **FGF** (median) | 0.00 | 0.00 | 0.00 | 0.176 |
| (P25, P75) | (-15.65, 30.47) | (-26.45, 12.87) | (-19.60, 23.04) |  |
| ∆ ≥15% | 41.2 | 22.4 | 25.9 |  |
| ∇ ≥15% | 25.5 | 36.7 | 29.6 |  |
|  |  |  |  |  |
| **GM-CSF** (median) | 0.00 | 0.00 | 0.00 | 0.792 |
| (P25, P75) | (-14.42, 20.50) | (-12.63, 61.56) | (-17.94, 34.45) |  |
| ∆ ≥15% | 29.4 | 34.7 | 29.6 |  |
| ∇ ≥15% | 23.5 | 22.4 | 25.9 |  |
|  |  |  |  |  |
| **HGF** (median) | 7.81 | 4.87 | 4.00 | 0.011 |
| (P25, P75) | (3.50, 11.94) | (0.60, 9.52) | (-1.16, 7.43) |  |
| ∆ ≥15% | 13.7 | 10.2 | 3.7 |  |
| ∇ ≥15% | 0.0 | 8.2 | 1.9 |  |
|  |  |  |  |  |
| **VEGF** (median) | 17.03 | 2.62 | 5.45 | 0.299 |
| (P25, P75) | (-1.41, 71.24) | (-7.89, 37.69) | (-9.07, 26.70) |  |
| ∆ ≥15% | 51.0 | 34.7 | 42.6 |  |
| ∇ ≥15% | 17.6 | 18.4 | 16.7 |  |
| **Chemokines** |  |  |  |  |
| **IL-8** (median) | 8.67 | 6.34 | 9.30 | 0.963 |
| (P25, P75) | (0.70, 16.87) | (-1.09, 19.17) | (0.23, 15.33) |  |
| ∆ ≥15% | 29.4 | 30.6 | 25.9 |  |
| ∇ ≥15% | 2.0 | 2.0 | 0.0 |  |
|  |  |  |  |  |
| **IP-10** (median) | -94.40 | -99.23 | -96.41 | 0.791 |
| (P25, P75) | (-134.5, -68.95) | (-167.1, -66.62) | (-149.3, -58.51) |  |
| ∆ ≥15% | 5.9 | 4.1 | 3.7 |  |
| ∇ ≥15% | 94.1 | 87.8 | 90.7 |  |
|  |  |  |  |  |
| **RANTES** (median) | 0.00 | -0.62 | 0.00 | 0.346 |
| (P25, P75) | (-0.96, 0.63) | (-2.73, 0.58) | (-1.72, 0.82) |  |
| ∆ ≥15% | 0.0 | 0.0 | 0.0 |  |
| ∇ ≥15% | 0.0 | 0.0 | 0.0 |  |
|  |  |  |  |  |
| **EOTAXIN** (median) | 0.04 | -1.14 | -0.51 | 0.640 |
| (P25, P75) | (-4.55, 4.70) | (-5.18, 3.62) | (-4.89, 6.88) |  |
| ∆ ≥15% | 3.9 | 2.0 | 7.4 |  |
| ∇ ≥15% | 7.8 | 0.0 | 3.7 |  |
|  |  |  |  |  |
| **MIP-1α** (median) | 9.03 | 0.00 | 0.00 | 0.026 |
| (P25, P75) | (-1.09, 71.25) | (-17.58, 13.17) | (-7.29, 10.07) |  |
| ∆ ≥15% | 41.2 | 24.5 | 18.5 |  |
| ∇ ≥15% | 15.7 | 24.5 | 18.5 |  |
|  |  |  |  |  |
| **MIP-1β** (median) | 4.06 | 1.29 | 1.87 | 0.182 |
| (P25, P75) | (-2.50, 16.79) | (-4.97, 10.88) | (-3.38, 6.53) |  |
| ∆ ≥15% | 29.4 | 12.2 | 5.6 |  |
| ∇ ≥15% | 2.0 | 12.2 | 5.6 |  |
|  |  |  |  |  |

[ continued ]

**Supplemental Table 5b.** Continued

|  |  | | |  |
| --- | --- | --- | --- | --- |
| **Cytokine^a^** | **<46 years**  (N = 51) | **46 – 62 years**  (N = 49) | **≥63 years**  (N = 54) | **P^b^** |
|  |  |  |  |  |
| **MCP-1** (median) | 2.20 | 0.46 | 1.40 | 0.207 |
| (P25, P75) | (-0.82, 4.27) | (-2.66, 3.13) | (-3.64, 4.42) |  |
| ∆ ≥15% | 0.0 | 2.0 | 0.0 |  |
| ∇ ≥15% | 0.0 | 4.1 | 0.0 |  |
|  |  |  |  |  |
| **MIG** (median) | 0.00 | 0.00 | 0.00 | 0.886 |
| (P25, P75) | (0.00, 0.00) | (-24.64, 0.00) | (0.00, 1.24) |  |
| ∆ ≥15% | 11.8 | 18.4 | 16.7 |  |
| ∇ ≥15% | 11.8 | 26.5 | 14.8 |  |
| **TH1** |  |  |  |  |
| **IL-2** (median) | 25.19 | -3.49 | 6.52 | 0.029 |
| (P25, P75) | (-11.46, 71.93) | (-40.32, 38.51) | (-10.83, 58.64) |  |
| ∆ ≥15% | 56.9 | 36.7 | 44.4 |  |
| ∇ ≥15% | 21.6 | 38.8 | 20.4 |  |
|  |  |  |  |  |
| **IL-12** (median) | 1.78 | 1.23 | 0.17 | 0.369 |
| (P25, P75) | (-3.99, 7.13) | (-5.79, 5.37) | (-7.38, 5.17) |  |
| ∆ ≥15% | 5.9 | 8.2 | 5.6 |  |
| ∇ ≥15% | 3.9 | 12.2 | 7.4 |  |
|  |  |  |  |  |
| **IFN-γ** (median) | 0.00 | 0.00 | 0.00 | 0.631 |
| (P25, P75) | (0.00, 0.00) | (0.00, 0.00) | (0.00, 0.00) |  |
| ∆ ≥15% | 9.8 | 6.1 | 13.0 |  |
| ∇ ≥15% | 9.8 | 8.2 | 7.4 |  |
| **TH2** |  |  |  |  |
| **IL-4** (median) | 0.00 | 0.00 | 0.00 | 0.315 |
| (P25, P75) | (0.00, 33.60) | (0.00, 0.00) | (0.00, 0.00) |  |
| ∆ ≥15% | 25.5 | 12.2 | 14.8 |  |
| ∇ ≥15% | 11.8 | 18.4 | 16.7 |  |
|  |  |  |  |  |
| **IL-5** (median) | 0.00 | 0.00 | 0.00 | 0.347 |
| (P25, P75) | (0.00, 70.06) | (-81.09, 25.40) | (-1.52, 0.00) |  |
| ∆ ≥15% | 31.4 | 28.6 | 20.4 |  |
| ∇ ≥15% | 17.6 | 26.5 | 20.4 |  |
|  |  |  |  |  |
| **IL-13** (median) | 0.00 | 0.00 | 0.00 | 0.617 |
| (P25, P75) | (-11.23, 77.18) | (-1.34, 7.25) | (-23.32, 27.07) |  |
| ∆ ≥15% | 37.3 | 20.4 | 27.8 |  |
| ∇ ≥15% | 21.6 | 20.4 | 29.6 |  |
| **Pro-inflammatory** |  |  |  |  |
| **IL-1β** (median) | 0.00 | 0.00 | 0.00 | 0.217 |
| (P25, P75) | (-8.14, 61.01) | (-8.71, 29.21) | (-32.29, 27.73) |  |
| ∆ ≥15% | 47.1 | 28.6 | 27.8 |  |
| ∇ ≥15% | 23.5 | 24.5 | 31.5 |  |
|  |  |  |  |  |
| **TNF-α** (median) | 25.69 | 6.95 | 0.00 | 0.420 |
| (P25, P75) | (-3.94, 89.82) | (-28.41, 86.99) | (0.00, 56.95) |  |
| ∆ ≥15% | 58.8 | 42.9 | 44.4 |  |
| ∇ ≥15% | 17.6 | 26.5 | 16.7 |  |
|  |  |  |  |  |
| **IL-6** (median) | 14.37 | 0.64 | 5.15 | 0.520 |
| (P25, P75) | (-7.81, 59.89) | (-6.80, 25.82) | (-7.48, 32.87) |  |
| ∆ ≥15% | 49.0 | 28.6 | 38.9 |  |
| ∇ ≥15% | 11.8 | 16.3 | 22.2 |  |
|  |  |  |  |  |
| **IFN-α** (median) | 14.14 | 0.00 | 0.00 | 0.206 |
| (P25, P75) | (-5.32, 36.80) | (-13.51, 20.79) | (-8.66, 15.53) |  |
| ∆ ≥15% | 47.1 | 30.6 | 24.1 |  |
| ∇ ≥15% | 13.7 | 18.4 | 14.8 |  |
|  |  |  |  |  |

[ continued ]

**Supplemental Table 5b.** Continued

|  |  | | |  |
| --- | --- | --- | --- | --- |
| **Cytokine^a^** | **<46 years**  (N = 51) | **46 – 62 years**  (N = 49) | **≥63 years**  (N = 54) | **P^b^** |
|  |  |  |  |  |
| **IL-2R** (median) | 7.92 | 0.00 | -0.55 | 0.081 |
| (P25, P75) | (-7.37, 18.47) | (-12.61, 9.94) | (-10.18, 10.29) |  |
| ∆ ≥15% | 29.4 | 16.3 | 22.2 |  |
| ∇ ≥15% | 9.8 | 22.4 | 16.7 |  |
|  |  |  |  |  |
| **IL-17** (median) | 0.00 | 0.00 | 0.00 | 0.382 |
| (P25, P75) | (0.00, 42.26) | (0.00, 2.83) | (-6.94, 30.14) |  |
| ∆ ≥15% | 29.4 | 20.4 | 29.6 |  |
| ∇ ≥15% | 13.7 | 16.3 | 24.1 |  |
| **Regulatory** |  |  |  |  |
| **IL-7** (median) | 0.00 | 0.00 | 0.00 | 0.151 |
| (P25, P75) | (-2.57, 30.93) | (-13.12, 13.82) | (-9.27, 9.64) |  |
| ∆ ≥15% | 31.4 | 22.4 | 20.4 |  |
| ∇ ≥15% | 9.8 | 24.5 | 20.4 |  |
| **Anti-inflammatory** |  |  |  |  |
| **IL-10** (median) | 0.00 | 0.00 | 0.00 | 0.242 |
| (P25, P75) | (-11.37, 43.89) | (-24.55, 50.80) | (-25.23, 2.77) |  |
| ∆ ≥15% | 37.3 | 32.7 | 20.4 |  |
| ∇ ≥15% | 19.6 | 28.6 | 27.8 |  |
|  |  |  |  |  |
| **IL-15** (median) | 0.00 | 0.00 | 0.00 | 0.035 |
| (P25, P75) | (0.00, 190.4) | (0.00, 0.00) | (0.00, 0.00) |  |
| ∆ ≥15% | 35.3 | 10.2 | 13.0 |  |
| ∇ ≥15% | 11.8 | 18.4 | 9.3 |  |
|  |  |  |  |  |
| **IL-1RA** (median) | 1.06 | -4.39 | -2.92 | 0.046 |
| (P25, P75) | (-8.24, 12.29) | (-12.23, 3.29) | (-9.62, 3.18) |  |
| ∆ ≥15% | 15.7 | 8.2 | 0.0 |  |
| ∇ ≥15% | 11.8 | 12.2 | 11.1 |  |
|  |  |  |  |  |

P25: percentile 25th. P75: percentile 75th.

Age groups are based on each participant's age in 2020-21.

^a^ Units used for computing the relative intraindividual change for cytokine levels were pg/mL, base 10 logtransformed.

^b^ Kruskal-Wallis test (two-tailed).

**Supplemental Table 6a.** Concentrations of 30 cytokines in 2016-17 by body mass index (N=240).

|  |  |  |  |  |
| --- | --- | --- | --- | --- |
| **Cytokine** (pg/mL) | **Underweight**  **& Normal**  (N = 101) | **Overweight**  (N = 91) | **Obese**  (N = 48) | **P^a^** |
| **Growth factors** |  |  |  |  |
| **G-CSF** (median) | 34.95 | 32.39 | 38.74 | 0.625 |
| (P25, P75) | (12.70, 112.5) | (8.09, 125.5) | (5.68, 94.35) |  |
| Geometric mean | 39.78 | 32.16 | 34.75 |  |
|  |  |  |  |  |
| **EGF** (median) | 86.53 | 77.88 | 89.97 | 0.869 |
| (P25, P75) | (41.99, 147.4) | (42.47, 200.8) | (39.94, 105.0) |  |
| Geometric mean | 74.16 | 91.30 | 73.15 |  |
|  |  |  |  |  |
| **FGF** (median) | 13.36 | 12.34 | 14.10 | 0.858 |
| (P25, P75) | (3.70, 31.49) | (6.26, 37.98) | (5.76, 33.42) |  |
| Geometric mean | 14.05 | 15.06 | 14.98 |  |
|  |  |  |  |  |
| **GM-CSF** (median) | 11.57 | 8.79 | 6.74 | 0.373 |
| (P25, P75) | (1.09, 63.06) | (0.74, 38.19) | (0.76, 40.79) |  |
| Geometric mean | 10.32 | 6.78 | 6.56 |  |
|  |  |  |  |  |
| **HGF** (median) | 363.6 | 375.9 | 377.1 | 0.935 |
| (P25, P75) | (273.9, 556.9) | (262.6, 497.2) | (233.4, 547.8) |  |
| Geometric mean | 406.1 | 369.4 | 355.0 |  |
|  |  |  |  |  |
| **VEGF** (median) | 5.33 | 5.46 | 5.35 | 0.627 |
| (P25, P75) | (2.74, 9.32) | (1.87, 9.63) | (2.61, 9.95) |  |
| Geometric mean | 4.66 | 4.06 | 5.25 |  |
| **Chemokines** |  |  |  |  |
| **IL-8** (median) | 14.40 | 16.42 | 17.68 | 0.057 |
| (P25, P75) | (10.52, 22.94) | (12.31, 23.20) | (14.73, 24.59) |  |
| Geometric mean | 14.78 | 16.37 | 18.86 |  |
|  |  |  |  |  |
| **IP-10** (median) | 6.71 | 6.68 | 6.50 | 0.873 |
| (P25, P75) | (4.10, 10.68) | (4.17, 10.18) | (3.83, 9.30) |  |
| Geometric mean | 7.12 | 6.62 | 6.15 |  |
|  |  |  |  |  |
| **RANTES** (median) | 3288 | 3481 | 3513 | 0.441 |
| (P25, P75) | (2842, 4211) | (2697, 4097) | (2995, 5061) |  |
| Geometric mean | 3483 | 3598 | 3878 |  |
|  |  |  |  |  |
| **EOTAXIN** (median) | 73.44 | 78.90 | 82.78 | 0.653 |
| (P25, P75) | (58.50, 100.3) | (60.57, 100.8) | (54.08, 111.8) |  |
| Geometric mean | 74.01 | 78.85 | 79.59 |  |
|  |  |  |  |  |
| **MIP-1α** (median) | 28.91 | 38.78 | 37.16 | 0.842 |
| (P25, P75) | (6.65, 115.8) | (5.90, 109.8) | (6.90, 132.6) |  |
| Geometric mean | 39.75 | 34.29 | 37.22 |  |
|  |  |  |  |  |
| **MIP-1β** (median) | 151.8 | 186.2 | 202.3 | 0.771 |
| (P25, P75) | (85.82, 510.3) | (104.2, 496.9) | (94.44, 338.0) |  |
| Geometric mean | 215.7 | 235.4 | 184.9 |  |
|  |  |  |  |  |
| **MCP-1** (median) | 645.0 | 647.1 | 695.6 | 0.971 |
| (P25, P75) | (488.1, 902.0) | (495.7, 843.5) | (497.9, 943.3) |  |
| Geometric mean | 736.5 | 697.1 | 670.1 |  |
|  |  |  |  |  |
| **MIG** (median) | 4.85 | 4.85 | 6.27 | 0.733 |
| (P25, P75) | (3.67, 20.63) | (3.67, 30.51) | (3.67, 30.51) |  |
| Geometric mean | 9.78 | 10.99 | 11.32 |  |
|  |  |  |  |  |

[ continued ]

**Supplemental Table 6a.** Continued.

|  |  |  |  |  |
| --- | --- | --- | --- | --- |
| **Cytokine** (pg/mL) | **Underweight**  **& Normal**  (N = 101) | **Overweight**  (N = 91) | **Obese**  (N = 48) | **P^a^** |
| **TH1** |  |  |  |  |
| **IL-2** (median) | 11.25 | 13.77 | 13.33 | 0.548 |
| (P25, P75) | (2.57, 70.52) | (3.63, 92.22) | (1.97, 47.75) |  |
| Geometric mean | 13.61 | 17.46 | 10.25 |  |
|  |  |  |  |  |
| **IL-12** (median) | 56.96 | 58.25 | 61.20 | 0.982 |
| (P25, P75) | (41.88, 88.80) | (39.28, 89.00) | (40.14, 95.77) |  |
| Geometric mean | 64.19 | 56.40 | 59.69 |  |
|  |  |  |  |  |
| **IFN-γ** (median) | 0.14 | 0.14 | 0.14 | 0.116 |
| (P25, P75) | (0.14, 0.21) | (0.14, 0.22) | (0.14, 0.22) |  |
| Geometric mean | 0.18 | 0.23 | 0.23 |  |
| **TH2** |  |  |  |  |
| **IL-4** (median) | 1.54 | 1.65 | 1.50 | 0.568 |
| (P25, P75) | (1.42, 4.40) | (1.42, 4.57) | (1.42, 6.84) |  |
| Geometric mean | 3.49 | 3.61 | 3.13 |  |
|  |  |  |  |  |
| **IL-5** (median) | 0.66 | 0.70 | 0.56 | 0.626 |
| (P25, P75) | (0.36, 1.11) | (0.37, 1.15) | (0.35, 0.95) |  |
| Geometric mean | 0.83 | 0.89 | 0.81 |  |
|  |  |  |  |  |
| **IL-13** (median) | 8.35 | 5.09 | 8.69 | 0.389 |
| (P25, P75) | (1.77, 15.97) | (1.21, 19.18) | (1.87, 15.00) |  |
| Geometric mean | 8.03 | 6.03 | 7.56 |  |
| **Pro-inflammatory** |  |  |  |  |
| **IL-1β** (median) | 0.79 | 0.91 | 1.20 | 0.133 |
| (P25, P75) | (0.27, 1.80) | (0.27, 1.80) | (0.57, 3.21) |  |
| Geometric mean | 1.00 | 0.98 | 1.44 |  |
|  |  |  |  |  |
| **TNF-α** (median) | 3.28 | 3.10 | 4.20 | 0.925 |
| (P25, P75) | (0.79, 13.65) | (1.34, 14.20) | (1.39, 17.89) |  |
| Geometric mean | 3.77 | 4.08 | 4.45 |  |
|  |  |  |  |  |
| **IL-6** (median) | 23.86 | 12.09 | 14.10 | 0.272 |
| (P25, P75) | (5.42, 62.13) | (4.36, 45.05) | (4.30, 59.62) |  |
| Geometric mean | 19.56 | 13.78 | 15.26 |  |
|  |  |  |  |  |
| **IFN-α** (median) | 15.11 | 22.46 | 21.44 | 0.771 |
| (P25, P75) | (6.35, 49.84) | (3.78, 68.36) | (8.30, 44.87) |  |
| Geometric mean | 18.71 | 20.50 | 19.11 |  |
|  |  |  |  |  |
| **IL-2R** (median) | 134.5 | 141.6 | 166.6 | 0.892 |
| (P25, P75) | (58.47, 424.3) | (50.92, 397.1) | (43.04, 444.5) |  |
| Geometric mean | 178.2 | 157.7 | 174.4 |  |
|  |  |  |  |  |
| **IL-17** (median) | 0.58 | 0.61 | 0.58 | 0.512 |
| (P25, P75) | (0.46, 1.59) | (0.55, 1.59) | (0.46, 1.51) |  |
| Geometric mean | 1.24 | 1.17 | 1.13 |  |
| **Regulatory** |  |  |  |  |
| **IL-7** (median) | 15.90 | 12.35 | 16.35 | 0.362 |
| (P25, P75) | (3.72, 31.06) | (2.49, 28.55) | (2.84, 40.01) |  |
| Geometric mean | 13.27 | 10.49 | 14.31 |  |
|  |  |  |  |  |

[ continued ]

**Supplemental Table 6a.** Continued.

|  |  |  |  |  |
| --- | --- | --- | --- | --- |
| **Cytokine** (pg/mL) | **Underweight**  **& Normal**  (N = 101) | **Overweight**  (N = 91) | **Obese**  (N = 48) | **P^a^** |
| **Anti-inflammatory** |  |  |  |  |
| **IL-10** (median) | 4.10 | 3.14 | 1.12 | 0.701 |
| (P25, P75) | (0.46, 44.49) | (0.50, 18.10) | (0.46, 34.69) |  |
| Geometric mean | 5.53 | 4.15 | 3.25 |  |
|  |  |  |  |  |
| **IL-15** (median) | 2.89 | 2.73 | 2.89 | 0.722 |
| (P25, P75) | (1.74, 36.91) | (1.74, 28.02) | (1.74, 79.21) |  |
| Geometric mean | 10.23 | 8.30 | 9.33 |  |
|  |  |  |  |  |
| **IL-1RA** (median) | 264.9 | 262.9 | 231.4 | 0.952 |
| (P25, P75) | (172.8, 506.6) | (163.4, 498.0) | (161.6, 500.7) |  |
| Geometric mean | 307.7 | 295.3 | 272.7 |  |
|  |  |  |  |  |

P25: percentile 25th. P75: percentile 75th.

<LOQ: value lower than the minimum concentration of the corresponding range of limits of quantification.

See Supplemental Table 1.

^a^ Kruskal-Wallis test (two-tailed).

**Supplemental Table 6b.** Relative intraindividual change (%) from 2016-17 to 2020-21 of concentrations of 30 cytokines, by body mass index (N=153).

|  |  | | |  |
| --- | --- | --- | --- | --- |
| **Cytokine^a^** | **Underweight & Normal**  (N = 68) | **Overweight**  (N = 49) | **Obese**  (N = 36) | **P^b^** |
| **Growth factors** |  |  |  |  |
| **G-CSF** (median) | 6.63 | 0.00 | 0.00 | 0.312 |
| (P25, P75) | (0.00, 32.79) | (-12.54, 18.37) | (-0.64, 16.38) |  |
| ∆ ≥15% | 38.2 | 26.5 | 33.3 |  |
| ∇ ≥15% | 13.2 | 22.4 | 13.9 |  |
|  |  |  |  |  |
| **EGF** (median) | 8.00 | 5.47 | 6.97 | 0.791 |
| (P25, P75) | (-3.54, 23.26) | (-3.77, 20.70) | (-1.65, 17.64) |  |
| ∆ ≥15% | 33.8 | 30.6 | 30.6 |  |
| ∇ ≥15% | 5.9 | 10.2 | 5.6 |  |
|  |  |  |  |  |
| **FGF** (median) | 0.00 | 0.00 | 0.00 | 0.095 |
| (P25, P75) | (-14.89, 21.51) | (-25.87, 24.05) | (-18.05, 24.74) |  |
| ∆ ≥15% | 30.9 | 28.6 | 30.6 |  |
| ∇ ≥15% | 25.0 | 38.8 | 27.8 |  |
|  |  |  |  |  |
| **GM-CSF** (median) | 0.00 | 0.00 | 0.00 | 0.169 |
| (P25, P75) | (-13.51, 30.87) | (-27.23, 13.33) | (0.00, 47.77) |  |
| ∆ ≥15% | 33.8 | 24.5 | 36.1 |  |
| ∇ ≥15% | 23.5 | 30.6 | 16.7 |  |
|  |  |  |  |  |
| **HGF** (median) | 5.42 | 5.38 | 6.50 | 0.197 |
| (P25, P75) | (0.79, 10.32) | (-0.41, 9.68) | (1.92, 10.43) |  |
| ∆ ≥15% | 7.4 | 12.2 | 8.3 |  |
| ∇ ≥15% | 5.9 | 2.0 | 0.0 |  |
|  |  |  |  |  |
| **VEGF** (median) | 8.96 | 6.94 | 2.29 | 0.948 |
| (P25, P75) | (-7.10, 39.93) | (-11.35, 32.65) | (-8.41, 27.83) |  |
| ∆ ≥15% | 45.6 | 42.9 | 38.9 |  |
| ∇ ≥15% | 16.2 | 22.4 | 13.9 |  |
| **Chemokines** |  |  |  |  |
| **IL-8** (median) | 8.44 | 10.54 | 5.78 | 0.203 |
| (P25, P75) | (0.48, 13.86) | (0.00, 17.68) | (-4.79, 14.61) |  |
| ∆ ≥15% | 23.5 | 38.8 | 22.2 |  |
| ∇ ≥15% | 1.5 | 0.0 | 2.8 |  |
|  |  |  |  |  |
| **IP-10** (median) | -96.08 | -86.50 | -110.6 | 0.582 |
| (P25, P75) | (-133.0, -64.69) | (-149.40, -56.45) | (-181.5, -67.82) |  |
| ∆ ≥15% | 2.9 | 6.1 | 5.6 |  |
| ∇ ≥15% | 91.2 | 91.8 | 88.9 |  |
|  |  |  |  |  |
| **RANTES** (median) | -0.11 | 0.00 | -0.51 | 0.534 |
| (P25, P75) | (-2.06, 0.60) | (-1.94, 1.11) | (-1.68, 0.43) |  |
| ∆ ≥15% | 0.0 | 0.0 | 0.0 |  |
| ∇ ≥15% | 0.0 | 0.0 | 0.0 |  |
|  |  |  |  |  |
| **EOTAXIN** (median) | -0.09 | -0.53 | -1.90 | 0.628 |
| (P25, P75) | (-4.47, 4.50) | (-5.14, 5.96) | (-5.36, 3.89) |  |
| ∆ ≥15% | 5.9 | 2.0 | 5.6 |  |
| ∇ ≥15% | 2.9 | 6.1 | 2.8 |  |
|  |  |  |  |  |
| **MIP-1α** (median) | 2.02 | 0.00 | 0.00 | 0.120 |
| (P25, P75) | (-11.52, 27.11) | (-8.87, 20.02) | (-2.86, 10.87) |  |
| ∆ ≥15% | 30.9 | 28.6 | 22.2 |  |
| ∇ ≥15% | 20.6 | 20.4 | 16.7 |  |
|  |  |  |  |  |
| **MIP-1β** (median) | 3.74 | 2.19 | 1.63 | 0.466 |
| (P25, P75) | (-3.06, 13.50) | (-6.70, 9.19) | (-2.47, 6.98) |  |
| ∆ ≥15% | 22.1 | 12.2 | 8.3 |  |
| ∇ ≥15% | 7.4 | 6.1 | 5.6 |  |
|  |  |  |  |  |

[ continued ]

**Supplemental Table 6b.** Continued

|  |  | | |  |
| --- | --- | --- | --- | --- |
| **Cytokine^a^** | **Underweight & Normal**  (N = 68) | **Overweight**  (N = 49) | **Obese**  (N = 36) | **P^b^** |
|  |  |  |  |  |
| **MCP-1** (median) | 0.11 | 0.87 | 2.19 | 0.749 |
| (P25, P75) | (-2.85, 3.28) | (-2.87, 3.74) | (-1.18, 4.43) |  |
| ∆ ≥15% | 0.0 | 0.0 | 0.0 |  |
| ∇ ≥15% | 1.5 | 2.0 | 0.0 |  |
|  |  |  |  |  |
| **MIG** (median) | 0.00 | 0.00 | 0.00 | 0.695 |
| (P25, P75) | (-4.01, 0.00) | (0.00, 0.00) | (-0.00, 0.00) |  |
| ∆ ≥15% | 16.2 | 18.4 | 11.1 |  |
| ∇ ≥15% | 17.6 | 16.3 | 19.4 |  |
| **TH1** |  |  |  |  |
| **IL-2** (median) | 19.60 | 2.58 | 1.80 | 0.183 |
| (P25, P75) | (-18.66, 55.26) | (-19.20, 63.14) | (-10.75, 42.79) |  |
| ∆ ≥15% | 51.5 | 44.9 | 36.1 |  |
| ∇ ≥15% | 26.5 | 30.6 | 22.2 |  |
|  |  |  |  |  |
| **IL-12** (median) | 1.71 | -1.55 | 1.20 | 0.395 |
| (P25, P75) | (-4.63, 6.61) | (-8.64, 5.73) | (-4.88, 5.10) |  |
| ∆ ≥15% | 5.9 | 6.1 | 8.3 |  |
| ∇ ≥15% | 8.8 | 10.2 | 2.8 |  |
|  |  |  |  |  |
| **IFN-γ** (median) | 0.00 | 0.00 | 0.00 | 0.822 |
| (P25, P75) | (0.00, 0.00) | (0.00, 0.00) | (0.00, 0.00) |  |
| ∆ ≥15% | 13.2 | 6.1 | 8.3 |  |
| ∇ ≥15% | 10.3 | 8.2 | 5.6 |  |
| **TH2** |  |  |  |  |
| **IL-4** (median) | 0.00 | 0.00 | 0.00 | 0.976 |
| (P25, P75) | (0.00, 7.00) | (0.00, 0.00) | (-13.45, 0.00) |  |
| ∆ ≥15% | 20.6 | 16.3 | 13.9 |  |
| ∇ ≥15% | 16.2 | 8.2 | 25.0 |  |
|  |  |  |  |  |
| **IL-5** (median) | 0.00 | 0.00 | 0.00 | 0.394 |
| (P25, P75) | (-4.56, 13.80) | (-14.62, 0.00) | (0.00, 80.95) |  |
| ∆ ≥15% | 23.5 | 22.4 | 38.9 |  |
| ∇ ≥15% | 23.5 | 24.5 | 13.9 |  |
|  |  |  |  |  |
| **IL-13** (median) | 0.00 | 0.00 | 0.00 | 0.322 |
| (P25, P75) | (-3.85, 39.85) | (-20.97, 29.59) | (-31.71, 13.69) |  |
| ∆ ≥15% | 33.8 | 26.5 | 22.2 |  |
| ∇ ≥15% | 17.6 | 26.5 | 33.3 |  |
| **Pro-inflammatory** |  |  |  |  |
| **IL-1β** (median) | 0.00 | 0.00 | 0.00 | 0.576 |
| (P25, P75) | (-15.09, 48.43) | (-23.35, 4.39) | (-43.16, 61.01) |  |
| ∆ ≥15% | 39.7 | 20.4 | 41.7 |  |
| ∇ ≥15% | 25.0 | 26.5 | 30.6 |  |
|  |  |  |  |  |
| **TNF-α** (median) | 25.48 | 0.00 | 0.00 | 0.176 |
| (P25, P75) | (0.00, 91.95) | (-1.97, 49.16) | (-10.19, 86.99) |  |
| ∆ ≥15% | 55.9 | 44.9 | 38.9 |  |
| ∇ ≥15% | 19.1 | 20.4 | 22.2 |  |
|  |  |  |  |  |
| **IL-6** (median) | 2.79 | 3.76 | 5.40 | 0.968 |
| (P25, P75) | (-6.69, 20.89) | (-9.10, 37.04) | (-6.64, 58.05) |  |
| ∆ ≥15% | 35.3 | 38.8 | 44.4 |  |
| ∇ ≥15% | 10.3 | 22.4 | 22.2 |  |
|  |  |  |  |  |
| **IFN-α** (median) | 5.22 | 0.00 | 2.24 | 0.300 |
| (P25, P75) | (-8.32, 26.69) | (-9.21, 20.21) | (-9.69, 19.09) |  |
| ∆ ≥15% | 36.8 | 28.6 | 33.3 |  |
| ∇ ≥15% | 14.7 | 16.3 | 16.7 |  |
|  |  |  |  |  |

[ continued ]

**Supplemental Table 6b.** Continued

|  |  | | |  |
| --- | --- | --- | --- | --- |
| **Cytokine^a^** | **Underweight & Normal**  (N = 68) | **Overweight**  (N = 49) | **Obese**  (N = 36) | **P^b^** |
|  |  |  |  |  |
| **IL-2R** (median) | 1.51 | -2.33 | 2.27 | 0.338 |
| (P25, P75) | (-8.92, 14.73) | (-11.73, 12.89) | (-7.63, 11.88) |  |
| ∆ ≥15% | 23.5 | 20.4 | 22.2 |  |
| ∇ ≥15% | 16.2 | 18.4 | 13.9 |  |
|  |  |  |  |  |
| **IL-17** (median) | 0.00 | 0.00 | 0.00 | 0.954 |
| (P25, P75) | (0.00, 42.26) | (0.00, 0.00) | (-0.29, 0.00) |  |
| ∆ ≥15% | 32.4 | 22.4 | 22.2 |  |
| ∇ ≥15% | 16.2 | 18.4 | 22.2 |  |
| **Regulatory** |  |  |  |  |
| **IL-7** (median) | 0.00 | 0.00 | 0.00 | 0.853 |
| (P25, P75) | (-2.20, 17.88) | (-9.06, 13.06) | (-17.34, 16.60) |  |
| ∆ ≥15% | 26.5 | 20.4 | 25.0 |  |
| ∇ ≥15% | 13.2 | 18.4 | 27.8 |  |
| **Anti-inflammatory** |  |  |  |  |
| **IL-10** (median) | 0.00 | 0.00 | 0.00 | 0.188 |
| (P25, P75) | (-21.37, 55.37) | (-20.71, 2.69) | (-2.72, 34.34) |  |
| ∆ ≥15% | 33.8 | 20.4 | 36.1 |  |
| ∇ ≥15% | 26.5 | 26.5 | 22.2 |  |
|  |  |  |  |  |
| **IL-15** (median) | 0.00 | 0.00 | 0.00 | 0.202 |
| (P25, P75) | (-6.73, 27.57) | (0.00, 0.00) | (0.00, 2.78) |  |
| ∆ ≥15% | 26.5 | 14.3 | 13.9 |  |
| ∇ ≥15% | 23.5 | 8.2 | 0.0 |  |
|  |  |  |  |  |
| **IL-1RA** (median) | -0.16 | -3.44 | -2.99 | 0.845 |
| (P25, P75) | (-8.36, 7.83) | (-12.99, 2.47) | (-9.00, 5.42) |  |
| ∆ ≥15% | 13.2 | 4.1 | 2.8 |  |
| ∇ ≥15% | 8.8 | 20.4 | 5.6 |  |
|  |  |  |  |  |

P25: percentile 25th. P75: percentile 75th.

Body mass index (BMI) is based on each participant's BMI in 2020-21.

^a^ Units used for computing the relative intraindividual change for cytokine levels were pg/mL, base 10 logtransformed.

^b^ Kruskal-Wallis test (two-tailed).

**Supplemental Table 7a.** Concentrations of 30 cytokines in 2016-17 by tobacco smoking (N=240).

|  |  |  |  |  |
| --- | --- | --- | --- | --- |
| **Cytokine** (pg/mL) | **Non-smoker**  (N = 93) | **Former smoker**  (N = 99) | **Current smoker**  (N = 48) | **P^a^** |
| **Growth factors** |  |  |  |  |
| **G-CSF** (median) | 51.85 | 31.84 | 33.67 | 0.625 |
| (P25, P75) | (7.19, 125.5) | (8.09, 125.5) | (12.91, 90.89) |  |
| Geometric mean | 40.88 | 32.50 | 33.42 |  |
|  |  |  |  |  |
| **EGF** (median) | 91.10 | 85.12 | 77.47 | 0.410 |
| (P25, P75) | (44.40, 206.1) | (39.62, 120.1) | (55.86, 149.1) |  |
| Geometric mean | 93.10 | 71.48 | 75.33 |  |
|  |  |  |  |  |
| **FGF** (median) | 13.36 | 12.34 | 12.97 | 0.984 |
| (P25, P75) | (5.06, 31.33) | (4.19, 41.38) | (4.47, 25.18) |  |
| Geometric mean | 15.13 | 14.42 | 14.02 |  |
|  |  |  |  |  |
| **GM-CSF** (median) | 7.55 | 6.40 | 15.69 | 0.056 |
| (P25, P75) | (0.74, 45.18) | (1.09, 32.54) | (2.34, 172.6) |  |
| Geometric mean | 7.59 | 6.03 | 16.29 |  |
|  |  |  |  |  |
| **HGF** (median) | 363.6 | 371.3 | 401.9 | 0.180 |
| (P25, P75) | (241.1, 491.9) | (262.6, 548.1) | (313.5, 612.7) |  |
| Geometric mean | 345.4 | 390.1 | 441.0 |  |
|  |  |  |  |  |
| **VEGF** (median) | 5.46 | 5.28 | 4.87 | 0.842 |
| (P25, P75) | (2.10, 8.60) | (2.31, 10.91) | (2.60, 8.83) |  |
| Geometric mean | 4.37 | 4.71 | 4.49 |  |
| **Chemokines** |  |  |  |  |
| **IL-8** (median) | 15.94 | 17.93 | 14.08 | 0.052 |
| (P25, P75) | (10.90, 22.40) | (13.14, 28.20) | (11.12, 21.64) |  |
| Geometric mean | 15.42 | 17.87 | 14.28 |  |
|  |  |  |  |  |
| **IP-10** (median) | 6.91 | 7.64 | 4.72 | 0.004 |
| (P25, P75) | (4.71, 10.08) | (4.43, 10.98) | (3.08, 8.16) |  |
| Geometric mean | 7.06 | 7.44 | 4.98 |  |
|  |  |  |  |  |
| **RANTES** (median) | 3361 | 3415 | 3489 | 0.642 |
| (P25, P75) | (2727, 4061) | (2829, 4101) | (2829, 5970) |  |
| Geometric mean | 3507 | 3622 | 3754 |  |
|  |  |  |  |  |
| **EOTAXIN** (median) | 71.20 | 78.37 | 86.56 | 0.076 |
| (P25, P75) | (53.62, 96.97) | (62.49, 101.7) | (60.18, 111.4) |  |
| Geometric mean | 72.03 | 78.92 | 82.85 |  |
|  |  |  |  |  |
| **MIP-1α** (median) | 38.78 | 28.91 | 25.35 | 0.406 |
| (P25, P75) | (7.64, 122.9) | (6.65, 113.6) | (5.90, 110.5) |  |
| Geometric mean | 44.53 | 33.29 | 32.54 |  |
|  |  |  |  |  |
| **MIP-1β** (median) | 190.1 | 151.8 | 177.2 | 0.542 |
| (P25, P75) | (103.8, 577.3) | (90.45, 332.5) | (87.12, 472.9) |  |
| Geometric mean | 247.0 | 200.1 | 196.0 |  |
|  |  |  |  |  |
| **MCP-1** (median) | 604.3 | 674.3 | 689.6 | 0.312 |
| (P25, P75) | (494.8, 832.3) | (471.3, 903.2) | (525.2, 1010) |  |
| Geometric mean | 681.5 | 717.0 | 741.5 |  |
|  |  |  |  |  |
| **MIG** (median) | 4.85 | 4.85 | 4.85 | 0.849 |
| (P25, P75) | (3.67, 23.74) | (3.67, 30.51) | (3.84, 18.74) |  |
| Geometric mean | 10.71 | 11.47 | 8.51 |  |
|  |  |  |  |  |

[ continued ]

**Supplemental Table 7a.** Continued.

|  |  |  |  |  |
| --- | --- | --- | --- | --- |
| **Cytokine** (pg/mL) | **Non-smoker**  (N = 93) | **Former smoker**  (N = 99) | **Current smoker**  (N = 48) | **P^a^** |
| **TH1** |  |  |  |  |
| **IL-2** (median) | 12.34 | 11.25 | 14.02 | 0.866 |
| (P25, P75) | (3.61, 78.34) | (2.20, 51.16) | (2.20, 86.42) |  |
| Geometric mean | 15.60 | 12.90 | 14.09 |  |
|  |  |  |  |  |
| **IL-12** (median) | 59.04 | 55.76 | 49.35 | 0.256 |
| (P25, P75) | (43.47, 92.83) | (42.88, 91.06) | (27.97, 75.77) |  |
| Geometric mean | 65.71 | 58.88 | 53.34 |  |
|  |  |  |  |  |
| **IFN-γ** (median) | 0.14 | 0.14 | 0.14 | 0.571 |
| (P25, P75) | (0.14, 0.22) | (0.14, 0.22) | (0.14, 0.22) |  |
| Geometric mean | 0.22 | 0.22 | 0.18 |  |
| **TH2** |  |  |  |  |
| **IL-4** (median) | 1.54 | 1.65 | 1.54 | 0.590 |
| (P25, P75) | (1.42, 3.94) | (1.42, 8.00) | (1.42, 3.88) |  |
| Geometric mean | 3.16 | 4.11 | 2.89 |  |
|  |  |  |  |  |
| **IL-5** (median) | 0.56 | 0.77 | 0.56 | 0.278 |
| (P25, P75) | (0.35, 1.11) | (0.37, 1.19) | (0.35, 1.11) |  |
| Geometric mean | 0.77 | 0.92 | 0.85 |  |
|  |  |  |  |  |
| **IL-13** (median) | 8.35 | 5.48 | 8.41 | 0.287 |
| (P25, P75) | (1.77, 15.34) | (1.21, 19.18) | (2.14, 19.18) |  |
| Geometric mean | 7.52 | 5.94 | 9.29 |  |
| **Pro-inflammatory** |  |  |  |  |
| **IL-1β** (median) | 0.91 | 0.91 | 0.82 | 0.904 |
| (P25, P75) | (0.33, 2.22) | (0.27, 1.94) | (0.27, 1.91) |  |
| Geometric mean | 1.14 | 1.08 | 0.92 |  |
|  |  |  |  |  |
| **TNF-α** (median) | 3.28 | 3.05 | 3.86 | 0.880 |
| (P25, P75) | (1.36, 15.56) | (1.13, 15.51) | (1.39, 12.01) |  |
| Geometric mean | 4.32 | 3.70 | 4.15 |  |
|  |  |  |  |  |
| **IL-6** (median) | 14.90 | 15.22 | 21.78 | 0.621 |
| (P25, P75) | (4.07, 46.71) | (4.63, 54.73) | (7.91, 66.50) |  |
| Geometric mean | 14.92 | 16.10 | 19.81 |  |
|  |  |  |  |  |
| **IFN-α** (median) | 20.76 | 18.22 | 19.73 | 0.681 |
| (P25, P75) | (5.89, 70.24) | (5.78, 47.74) | (9.02, 54.58) |  |
| Geometric mean | 21.07 | 17.27 | 21.32 |  |
|  |  |  |  |  |
| **IL-2R** (median) | 141.6 | 134.5 | 163.1 | 0.983 |
| (P25, P75) | (49.10, 502.2) | (49.91, 421.4) | (64.82, 388.8) |  |
| Geometric mean | 178.8 | 163.1 | 164.9 |  |
|  |  |  |  |  |
| **IL-17** (median) | 0.58 | 0.58 | 0.58 | 0.437 |
| (P25, P75) | (0.46, 1.99) | (0.55, 1.56) | (0.46, 1.45) |  |
| Geometric mean | 1.22 | 1.34 | 0.87 |  |
| **Regulatory** |  |  |  |  |
| **IL-7** (median) | 13.89 | 12.35 | 23.66 | 0.184 |
| (P25, P75) | (2.84, 27.70) | (2.49, 31.74) | (4.23, 32.51) |  |
| Geometric mean | 12.65 | 10.43 | 16.54 |  |
|  |  |  |  |  |

[ continued ]

**Supplemental Table 7a.** Continued.

|  |  |  |  |  |
| --- | --- | --- | --- | --- |
| **Cytokine** (pg/mL) | **Non-smoker**  (N = 93) | **Former smoker**  (N = 99) | **Current smoker**  (N = 48) | **P^a^** |
| **Anti-inflammatory** |  |  |  |  |
| **IL-10** (median) | 5.32 | 1.24 | 5.48 | 0.025 |
| (P25, P75) | (0.50, 38.87) | (0.46, 11.71) | (0.51, 90.25) |  |
| Geometric mean | 5.66 | 2.70 | 7.95 |  |
|  |  |  |  |  |
| **IL-15** (median) | 2.89 | 2.73 | 2.89 | 0.433 |
| (P25, P75) | (2.40, 56.60) | (1.74, 21.67) | (1.90, 48.21) |  |
| Geometric mean | 11.01 | 7.92 | 9.25 |  |
|  |  |  |  |  |
| **IL-1RA** (median) | 255.0 | 250.0 | 312.6 | 0.763 |
| (P25, P75) | (153.8, 500.7) | (166.0, 514.9) | (178.0, 474.7) |  |
| Geometric mean | 289.4 | 295.5 | 308.8 |  |
|  |  |  |  |  |

P25: percentile 25th. P75: percentile 75th.

<LOQ: value lower than the minimum concentration of the corresponding range of limits of quantification.

See Supplemental Table 1.

^a^ Kruskal-Wallis test (two-tailed).

**Supplemental Table 7b.** Relative intraindividual change (%) from 2016-17 to 2020-21 of concentrations of cytokines, by tobacco smoking (N=154).

|  |  | | |  |
| --- | --- | --- | --- | --- |
| **Cytokine^a^** | **Non-smoker**  (N = 59) | **Former smoker**  (N = 56) | **Current smoker**  (N = 39) | **P^b^** |
| **Growth factors** |  |  |  |  |
| **G-CSF** (median) | 0.00 | 3.68 | 2.68 | 0.046 |
| (P25, P75) | (-15.54, 18.29) | (0.00, 36.81) | (0.00, 16.96) |  |
| ∆ ≥15% | 28.8 | 41.1 | 28.2 |  |
| ∇ ≥15% | 25.4 | 10.7 | 10.3 |  |
|  |  |  |  |  |
| **EGF** (median) | 6.95 | 8.29 | 7.02 | 0.833 |
| (P25, P75) | (-3.40, 18.87) | (-3.00, 20.12) | (-3.59, 23.82) |  |
| ∆ ≥15% | 30.5 | 30.4 | 38.5 |  |
| ∇ ≥15% | 10.2 | 5.4 | 5.1 |  |
|  |  |  |  |  |
| **FGF** (median) | 0.00 | 0.00 | 0.00 | 0.310 |
| (P25, P75) | (-21.74, 22.08) | (-14.61, 22.68) | (-17.74, 25.42) |  |
| ∆ ≥15% | 28.8 | 32.1 | 28.2 |  |
| ∇ ≥15% | 39.0 | 25.0 | 25.6 |  |
|  |  |  |  |  |
| **GM-CSF** (median) | 0.00 | 0.00 | 0.00 | 0.081 |
| (P25, P75) | (-20.40, 10.39) | (0.00, 75.43) | (-18.67, 29.30) |  |
| ∆ ≥15% | 22.0 | 41.1 | 30.8 |  |
| ∇ ≥15% | 27.1 | 19.6 | 25.6 |  |
|  |  |  |  |  |
| **HGF** (median) | 4.87 | 5.38 | 6.00 | 0.575 |
| (P25, P75) | (0.00, 10.20) | (0.60, 10.20) | (2.01, 10.28) |  |
| ∆ ≥15% | 8.5 | 7.1 | 12.8 |  |
| ∇ ≥15% | 5.1 | 1.8 | 2.6 |  |
|  |  |  |  |  |
| **VEGF** (median) | 5.28 | 8.30 | 5.47 | 0.754 |
| (P25, P75) | (-7.48, 27.44) | (-8.41, 47.59) | (-7.91, 39.66) |  |
| ∆ ≥15% | 44.1 | 46.4 | 35.9 |  |
| ∇ ≥15% | 18.6 | 16.1 | 17.9 |  |
| **Chemokines** |  |  |  |  |
| **IL-8** (median) | 6.92 | 11.98 | 4.89 | 0.239 |
| (P25, P75) | (0.00, 14.41) | (1.74, 18.57) | (0.00, 17.65) |  |
| ∆ ≥15% | 23.7 | 32.1 | 30.8 |  |
| ∇ ≥15% | 0.0 | 0.0 | 5.1 |  |
|  |  |  |  |  |
| **IP-10** (median) | -97.17 | -98.79 | -81.10 | 0.901 |
| (P25, P75) | (-160.8, -61.36) | (-135.3, -68.52) | (-206.2, -48.71) |  |
| ∆ ≥15% | 5.1 | 3.6 | 5.1 |  |
| ∇ ≥15% | 94.9 | 89.3 | 87.2 |  |
|  |  |  |  |  |
| **RANTES** (median) | 0.00 | -0.24 | 0.00 | 0.320 |
| (P25, P75) | (-1.73, 1.12) | (-2.73, 0.43) | (-1.23, 0.24) |  |
| ∆ ≥15% | 0.0 | 0.0 | 0.0 |  |
| ∇ ≥15% | 0.0 | 0.0 | 0.0 |  |
|  |  |  |  |  |
| **EOTAXIN** (median) | -0.74 | -0.43 | -0.11 | 0.617 |
| (P25, P75) | (-3.95, 5.45) | (-5.06, 4.66) | (-5.12, 2.36) |  |
| ∆ ≥15% | 6.8 | 5.4 | 0.0 |  |
| ∇ ≥15% | 5.1 | 1.8 | 5.1 |  |
|  |  |  |  |  |
| **MIP-1α** (median) | 0.00 | 0.00 | 4.76 | 0.047 |
| (P25, P75) | (-20.67, 14.62) | (-0.58, 25.14) | (0.00, 29.50) |  |
| ∆ ≥15% | 23.7 | 28.6 | 33.3 |  |
| ∇ ≥15% | 32.2 | 12.5 | 10.3 |  |
|  |  |  |  |  |
| **MIP-1β** (median) | 1.12 | 4.01 | 0.47 | 0.452 |
| (P25, P75) | (-4.96, 7.99) | (-1.93, 10.22) | (-5.19, 14.05) |  |
| ∆ ≥15% | 16.9 | 10.7 | 20.5 |  |
| ∇ ≥15% | 10.2 | 1.8 | 7.7 |  |
|  |  |  |  |  |

[ continued ]

**Supplemental Table 7b.** Continued

|  |  | | |  |
| --- | --- | --- | --- | --- |
| **Cytokine^a^** | **Non-smoker**  (N = 59) | **Former smoker**  (N = 56) | **Current smoker**  (N = 39) | **P^b^** |
|  |  |  |  |  |
| **MCP-1** (median) | 1.45 | 0.76 | 0.87 | 0.940 |
| (P25, P75) | (-3.54, 4.35) | (-1.74, 3.58) | (-2.96, 3.82) |  |
| ∆ ≥15% | 1.7 | 0.0 | 0.0 |  |
| ∇ ≥15% | 1.7 | 1.8 | 0.0 |  |
|  |  |  |  |  |
| **MIG** (median) | 0.00 | 0.00 | 0.00 | 0.112 |
| (P25, P75) | (-12.36, 0.00) | (0.00, 3.82) | (0.00, 0.00) |  |
| ∆ ≥15% | 15.3 | 17.0 | 12.8 |  |
| ∇ ≥15% | 23.7 | 14.3 | 12.8 |  |
| **TH1** |  |  |  |  |
| **IL-2** (median) | 5.81 | 17.02 | 0.00 | 0.716 |
| (P25, P75) | (-19.69, 38.68) | (-10.58, 55.26) | (-24.46, 77.32) |  |
| ∆ ≥15% | 44.1 | 51.8 | 41.0 |  |
| ∇ ≥15% | 28.8 | 19.6 | 33.3 |  |
|  |  |  |  |  |
| **IL-12** (median) | -1.28 | 1.15 | 1.78 | 0.577 |
| (P25, P75) | (-7.71, 5.45) | (-4.49, 6.14) | (-5.46, 7.13) |  |
| ∆ ≥15% | 6.8 | 5.4 | 7.7 |  |
| ∇ ≥15% | 8.5 | 7.1 | 7.7 |  |
|  |  |  |  |  |
| **IFN-γ** (median) | 0.00 | 0.00 | 0.00 | 0.814 |
| (P25, P75) | (0.00, 0.00) | (0.00, 0.00) | (0.00, 0.00) |  |
| ∆ ≥15% | 10.2 | 7.1 | 12.8 |  |
| ∇ ≥15% | 11.9 | 3.6 | 10.3 |  |
| **TH2** |  |  |  |  |
| **IL-4** (median) | 0.00 | 0.00 | 0.00 | 0.359 |
| (P25, P75) | (0.00, 0.00) | (0.00, 0.00) | (0.00, 7.93) |  |
| ∆ ≥15% | 16.9 | 14.3 | 23.1 |  |
| ∇ ≥15% | 18.6 | 16.1 | 10.3 |  |
|  |  |  |  |  |
| **IL-5** (median) | 0.00 | 0.00 | 0.00 | 0.334 |
| (P25, P75) | (0.00, 60.54) | (-4.56, 0.00) | (0.00, 60.21) |  |
| ∆ ≥15% | 28.8 | 17.9 | 35.9 |  |
| ∇ ≥15% | 20.3 | 21.4 | 23.1 |  |
|  |  |  |  |  |
| **IL-13** (median) | 0.00 | 0.00 | 0.00 | 0.679 |
| (P25, P75) | (-26.06, 34.41) | (0.00, 20.92) | (-12.03, 10.47) |  |
| ∆ ≥15% | 32.2 | 28.6 | 23.1 |  |
| ∇ ≥15% | 32.2 | 16.1 | 23.1 |  |
| **Pro-inflammatory** |  |  |  |  |
| **IL-1β** (median) | 0.00 | 0.00 | 0.00 | 0.738 |
| (P25, P75) | (-32.05, 56.60) | (-4.41, 27.90) | (-19.07, 47.18) |  |
| ∆ ≥15% | 33.9 | 28.6 | 43.6 |  |
| ∇ ≥15% | 30.5 | 21.4 | 28.2 |  |
|  |  |  |  |  |
| **TNF-α** (median) | 0.00 | 27.56 | 12.93 | 0.189 |
| (P25, P75) | (-16.79, 69.46) | (0.00, 65.16) | (0.00, 109.11) |  |
| ∆ ≥15% | 44.1 | 53.6 | 48.7 |  |
| ∇ ≥15% | 30.5 | 12.5 | 15.4 |  |
|  |  |  |  |  |
| **IL-6** (median) | 5.10 | 6.76 | 3.06 | 0.846 |
| (P25, P75) | (-6.72, 32.02) | (-5.92, 49.58) | (-8.06, 35.44) |  |
| ∆ ≥15% | 37.3 | 41.1 | 38.5 |  |
| ∇ ≥15% | 18.6 | 17.9 | 12.8 |  |
|  |  |  |  |  |
| **IFN-α** (median) | 1.57 | 3.57 | 0.00 | 0.909 |
| (P25, P75) | (-13.88, 36.03) | (-2.90, 20.28) | (-11.54, 62.96) |  |
| ∆ ≥15% | 35.6 | 32.1 | 33.3 |  |
| ∇ ≥15% | 20.3 | 7.1 | 20.5 |  |
|  |  |  |  |  |

[ continued ]

**Supplemental Table 7b.** Continued

|  |  | | |  |
| --- | --- | --- | --- | --- |
| **Cytokine^a^** | **Non-smoker**  (N = 59) | **Former smoker**  (N = 56) | **Current smoker**  (N = 39) | **P^b^** |
|  |  |  |  |  |
| **IL-2R** (median) | -2.33 | 2.21 | 1.46 | 0.280 |
| (P25, P75) | (-13.14, 13.33) | (-7.13, 10.49) | (-7.78, 20.57) |  |
| ∆ ≥15% | 20.3 | 17.9 | 33.3 |  |
| ∇ ≥15% | 20.3 | 12.5 | 15.4 |  |
|  |  |  |  |  |
| **IL-17** (median) | 0.00 | 0.00 | 0.00 | 0.353 |
| (P25, P75) | (-28.56, 27.60) | (0.00, 22.77) | (0.00, 41.87) |  |
| ∆ ≥15% | 25.4 | 26.8 | 28.2 |  |
| ∇ ≥15% | 25.4 | 14.3 | 12.8 |  |
| **Regulatory** |  |  |  |  |
| **IL-7** (median) | 0.00 | 0.00 | 0.56 | 0.674 |
| (P25, P75) | (-6.19, 17.67) | (-6.89, 12.98) | (-6.78, 14.09) |  |
| ∆ ≥15% | 27.1 | 23.2 | 23.1 |  |
| ∇ ≥15% | 18.6 | 21.4 | 12.8 |  |
| **Anti-inflammatory** |  |  |  |  |
| **IL-10** (median) | 0.00 | 0.00 | 0.00 | 0.820 |
| (P25, P75) | (-25.14, 32.41) | (-22.20, 29.35) | (-6.34, 43.89) |  |
| ∆ ≥15% | 28.8 | 30.4 | 30.8 |  |
| ∇ ≥15% | 27.1 | 28.6 | 17.9 |  |
|  |  |  |  |  |
| **IL-15** (median) | 0.00 | 0.00 | 0.00 | 0.699 |
| (P25, P75) | (0.00, 0.58) | (0.00, 0.00) | (0.00, 33.33) |  |
| ∆ ≥15% | 18.6 | 14.3 | 28.2 |  |
| ∇ ≥15% | 13.6 | 10.7 | 15.4 |  |
|  |  |  |  |  |
| **IL-1RA** (median) | -1.41 | -2.98 | -2.32 | 0.758 |
| (P25, P75) | (-12.10, 5.90) | (-9.00, 5.44) | (-7.17, 3.65) |  |
| ∆ ≥15% | 8.5 | 7.1 | 7.7 |  |
| ∇ ≥15% | 16.9 | 7.1 | 10.3 |  |
|  |  |  |  |  |

P25: percentile 25th. P75: percentile 75th.

Tobacco smoking groups are based on participant's data in 2020-21.

^a^ Units used for computing the relative intraindividual change for cytokine levels were pg/mL, base 10 logtransformed.

^b^ Kruskal-Wallis test (two-tailed).

**Supplemental Table 8a.** Concentrations of 30 cytokines in 2016-17 by educational level (N=240).

|  |  |  |  |  |
| --- | --- | --- | --- | --- |
| **Cytokine** (pg/mL) | **Primary or less**  (N = 56) | **Secondary**  (N = 65) | **University**  (N = 119) | **P^a^** |
| **Growth factors** |  |  |  |  |
| **G-CSF** (median) | 33.35 | 27.24 | 34.95 | 0.725 |
| (P25, P75) | (8.89, 121.1) | (5.68, 100.2) | (11.30, 122.9) |  |
| Geometric mean | 36.39 | 30.23 | 38.79 |  |
|  |  |  |  |  |
| **EGF** (median) | 73.13 | 72.05 | 91.55 | 0.167 |
| (P25, P75) | (39.06, 114.5) | (33.73, 119.7) | (48.29, 191.0) |  |
| Geometric mean | 80.21 | 66.53 | 88.42 |  |
|  |  |  |  |  |
| **FGF** (median) | 12.09 | 13.36 | 13.85 | 0.525 |
| (P25, P75) | (3.70, 30.74) | (3.86, 28.81) | (6.66, 44.71) |  |
| Geometric mean | 13.33 | 12.82 | 16.38 |  |
|  |  |  |  |  |
| **GM-CSF** (median) | 11.68 | 4.70 | 10.25 | 0.225 |
| (P25, P75) | (1.09, 57.03) | (0.53, 34.72) | (1.09, 51.60) |  |
| Geometric mean | 10.01 | 5.41 | 9.00 |  |
|  |  |  |  |  |
| **HGF** (median) | 369.7 | 371.7 | 363.6 | 0.368 |
| (P25, P75) | (310.5, 553.6) | (221.5, 584.0) | (246.0, 501.7) |  |
| Geometric mean | 450.4 | 375.2 | 355.8 |  |
|  |  |  |  |  |
| **VEGF** (median) | 5.24 | 4.48 | 5.69 | 0.425 |
| (P25, P75) | (2.55, 10.11) | (2.01, 7.95) | (2.59, 10.32) |  |
| Geometric mean | 4.95 | 4.07 | 4.60 |  |
| **Chemokines** |  |  |  |  |
| **IL-8** (median) | 18.75 | 15.39 | 16.42 | 0.629 |
| (P25, P75) | (12.39, 27.31) | (12.00, 22.78) | (11.17, 22.83) |  |
| Geometric mean | 15.68 | 15.72 | 16.58 |  |
|  |  |  |  |  |
| **IP-10** (median) | 5.95 | 7.64 | 6.68 | 0.470 |
| (P25, P75) | (4.03, 9.57) | (4.30, 10.33) | (3.98, 10.18) |  |
| Geometric mean | 6.06 | 7.17 | 6.82 |  |
|  |  |  |  |  |
| **RANTES** (median) | 3444 | 3361 | 3407 | 0.986 |
| (P25, P75) | (2972, 4127) | (2694, 4706) | (2804, 4101) |  |
| Geometric mean | 3525 | 3686 | 3595 |  |
|  |  |  |  |  |
| **EOTAXIN** (median) | 83.55 | 78.90 | 72.07 | 0.134 |
| (P25, P75) | (62.94, 111.8) | (56.21, 99.45) | (57.21, 104.4) |  |
| Geometric mean | 86.90 | 72.08 | 75.26 |  |
|  |  |  |  |  |
| **MIP-1α** (median) | 25.35 | 28.91 | 38.78 | 0.805 |
| (P25, P75) | (6.65, 95.02) | (6.65, 122.9) | (6.65, 127.7) |  |
| Geometric mean | 35.54 | 35.73 | 38.62 |  |
|  |  |  |  |  |
| **MIP-1β** (median) | 136.0 | 179.4 | 190.2 | 0.279 |
| (P25, P75) | (84.12, 337.1) | (111.2, 439.4) | (91.02, 496.9) |  |
| Geometric mean | 185.6 | 225.1 | 227.3 |  |
|  |  |  |  |  |
| **MCP-1** (median) | 629.2 | 671.4 | 654.1 | 0.951 |
| (P25, P75) | (487.2, 919.0) | (506.6, 896.4) | (492.8, 863.8) |  |
| Geometric mean | 733.7 | 696.7 | 702.0 |  |
|  |  |  |  |  |
| **MIG** (median) | 5.56 | 4.85 | 4.85 | 0.425 |
| (P25, P75) | (3.67, 20.63) | (3.67, 20.63) | (3.67, 30.51) |  |
| Geometric mean | 10.14 | 8.84 | 11.77 |  |
|  |  |  |  |  |

[ continued ]

**Supplemental Table 8a.** Continued.

|  |  |  |  |  |
| --- | --- | --- | --- | --- |
| **Cytokine** (pg/mL) | **Primary or less**  (N = 56) | **Secondary**  (N = 65) | **University**  (N = 119) | **P^a^** |
| **TH1** |  |  |  |  |
| **IL-2** (median) | 7.51 | 13.77 | 14.27 | 0.271 |
| (P25, P75) | (1.13, 42.65) | (2.20, 67.45) | (3.63, 79.15) |  |
| Geometric mean | 10.48 | 13.90 | 16.42 |  |
|  |  |  |  |  |
| **IL-12** (median) | 58.82 | 56.14 | 58.25 | 0.672 |
| (P25, P75) | (44.04, 97.55) | (33.42, 87.45) | (41.86, 86.71) |  |
| Geometric mean | 67.85 | 56.38 | 59.06 |  |
|  |  |  |  |  |
| **IFN-γ** (median) | 0.14 | 0.14 | 0.14 | 0.218 |
| (P25, P75) | (0.14, 0.20) | (0.14, 0.22) | (0.14, 0.22) |  |
| Geometric mean | 0.19 | 0.19 | 0.23 |  |
| **TH2** |  |  |  |  |
| **IL-4** (median) | 1.50 | 1.54 | 1.65 | 0.224 |
| (P25, P75) | (1.42, 3.94) | (1.42, 4.70) | (1.42, 4.81) |  |
| Geometric mean | 2.73 | 3.53 | 3.82 |  |
|  |  |  |  |  |
| **IL-5** (median) | 0.56 | 0.56 | 0.77 | 0.307 |
| (P25, P75) | (0.35, 1.11) | (0.37, 0.96) | (0.37, 1.15) |  |
| Geometric mean | 0.76 | 0.81 | 0.91 |  |
|  |  |  |  |  |
| **IL-13** (median) | 5.09 | 6.81 | 8.48 | 0.649 |
| (P25, P75) | (1.77, 15.84) | (1.21, 17.45) | (1.21, 19.18) |  |
| Geometric mean | 6.23 | 6.45 | 7.99 |  |
| **Pro-inflammatory** |  |  |  |  |
| **IL-1β** (median) | 1.09 | 0.69 | 0.91 | 0.581 |
| (P25, P75) | (0.43, 2.26) | (0.27, 1.78) | (0.27, 1.94) |  |
| Geometric mean | 1.15 | 0.90 | 1.13 |  |
|  |  |  |  |  |
| **TNF-α** (median) | 2.92 | 2.91 | 4.37 | 0.556 |
| (P25, P75) | (1.18, 10.58) | (1.34, 15.56) | (1.34, 19.53) |  |
| Geometric mean | 3.36 | 3.74 | 4.54 |  |
|  |  |  |  |  |
| **IL-6** (median) | 21.44 | 13.87 | 16.37 | 0.345 |
| (P25, P75) | (6.23, 51.99) | (3.34, 45.39) | (4.63, 63.58) |  |
| Geometric mean | 20.73 | 12.41 | 16.88 |  |
|  |  |  |  |  |
| **IFN-α** (median) | 15.53 | 18.22 | 22.44 | 0.646 |
| (P25, P75) | (6.88, 47.25) | (7.00, 50.41) | (5.89, 66.64) |  |
| Geometric mean | 18.43 | 17.91 | 20.88 |  |
|  |  |  |  |  |
| **IL-2R** (median) | 101.7 | 196.2 | 134.5 | 0.624 |
| (P25, P75) | (49.91, 405.1) | (59.98, 416.4) | (56.65, 567.7) |  |
| Geometric mean | 148.2 | 172.2 | 178.8 |  |
|  |  |  |  |  |
| **IL-17** (median) | 0.58 | 0.61 | 0.58 | 0.419 |
| (P25, P75) | (0.46, 1.51) | (0.46, 1.58) | (0.46, 1.83) |  |
| Geometric mean | 1.00 | 1.14 | 1.32 |  |
| **Regulatory** |  |  |  |  |
| **IL-7** (median) | 20.55 | 12.35 | 14.01 | 0.421 |
| (P25, P75) | (3.06, 32.51) | (2.84, 29.85) | (2.84, 30.37) |  |
| Geometric mean | 14.46 | 10.89 | 12.24 |  |
|  |  |  |  |  |

[ continued ]

**Supplemental Table 8a.** Continued.

|  |  |  |  |  |
| --- | --- | --- | --- | --- |
| **Cytokine** (pg/mL) | **Primary or less**  (N = 56) | **Secondary**  (N = 65) | **University**  (N = 119) | **P^a^** |
| **Anti-inflammatory** |  |  |  |  |
| **IL-10** (median) | 4.03 | 0.55 | 4.18 | 0.281 |
| (P25, P75) | (0.50, 39.83) | (0.41, 22.76) | (0.46, 36.85) |  |
| Geometric mean | 5.07 | 3.08 | 5.15 |  |
|  |  |  |  |  |
| **IL-15** (median) | 2.89 | 2.73 | 2.89 | 0.491 |
| (P25, P75) | (1.90, 6.28) | (1.74, 21.32) | (1.74, 63.66) |  |
| Geometric mean | 7.34 | 8.30 | 11.01 |  |
|  |  |  |  |  |
| **IL-1RA** (median) | 211.2 | 255.0 | 291.7 | 0.325 |
| (P25, P75) | (155.1, 388.9) | (157.2, 511.7) | (182.4, 516.7) |  |
| Geometric mean | 271.2 | 292.3 | 310.0 |  |
|  |  |  |  |  |

P25: percentile 25th. P75: percentile 75th.

<LOQ: value lower than the minimum concentration of the corresponding range of limits of quantification.

See Supplemental Table 1.

^a^ Kruskal-Wallis test (two-tailed).

**Supplemental Table 8b.** Relative intraindividual change (%) from 2016-17 to 2020-21 of concentrations of cytokines, by educational level (N=154).

|  |  | | |  |
| --- | --- | --- | --- | --- |
| **Cytokine^a^** | **Primary or less** (N = 35) | **Secondary**  (N = 42) | **University**  (N = 77) | **P^b^** |
| **Growth factors** |  |  |  |  |
| **G-CSF** (median) | 0.00 | 6.69 | 0.00 | 0.160 |
| (P25, P75) | (-7.42, 18.29) | (0.00, 21.51) | (-10.73, 27.27) |  |
| ∆ ≥15% | 28.6 | 40.5 | 31.2 |  |
| ∇ ≥15% | 22.9 | 4.8 | 19.5 |  |
|  |  |  |  |  |
| **EGF** (median) | 6.95 | 8.54 | 7.88 | 0.597 |
| (P25, P75) | (-3.01, 21.89) | (-0.80, 22.28) | (-4.63, 19.88) |  |
| ∆ ≥15% | 31.4 | 35.7 | 31.2 |  |
| ∇ ≥15% | 11.4 | 4.8 | 6.5 |  |
|  |  |  |  |  |
| **FGF** (median) | 0.00 | 0.00 | 0.00 | 0.733 |
| (P25, P75) | (-21.74, 32.23) | (-16.96, 30.97) | (-20.09, 19.33) |  |
| ∆ ≥15% | 34.3 | 31.0 | 27.3 |  |
| ∇ ≥15% | 31.4 | 28.6 | 31.2 |  |
|  |  |  |  |  |
| **GM-CSF** (median) | 0.00 | 5.51 | 0.00 | 0.125 |
| (P25, P75) | (-26.04, 30.06) | (-1.74, 85.06) | (-16.79, 17.31) |  |
| ∆ ≥15% | 28.6 | 42.9 | 26.0 |  |
| ∇ ≥15% | 28.6 | 16.7 | 26.0 |  |
|  |  |  |  |  |
| **HGF** (median) | 5.26 | 4.13 | 5.81 | 0.967 |
| (P25, P75) | (1.88, 9.96) | (0.88, 10.23) | (0.54, 10.10) |  |
| ∆ ≥15% | 5.7 | 7.1 | 11.7 |  |
| ∇ ≥15% | 2.9 | 2.4 | 3.9 |  |
|  |  |  |  |  |
| **VEGF** (median) | 11.45 | 14.85 | 4.84 | 0.392 |
| (P25, P75) | (-1.41, 50.11) | (-9.99, 28.53) | (-10.04, 34.84) |  |
| ∆ ≥15% | 48.6 | 50.0 | 36.4 |  |
| ∇ ≥15% | 8.6 | 16.7 | 22.1 |  |
| **Chemokines** |  |  |  |  |
| **IL-8** (median) | 6.92 | 5.20 | 9.56 | 0.494 |
| (P25, P75) | (0.00, 15.21) | (-2.70, 16.03) | (1.79, 17.62) |  |
| ∆ ≥15% | 25.7 | 28.6 | 29.9 |  |
| ∇ ≥15% | 0.0 | 2.4 | 1.3 |  |
|  |  |  |  |  |
| **IP-10** (median) | -108.8 | -80.59 | -96.06 | 0.426 |
| (P25, P75) | (-189.5, -80.11) | (-147.8, -61.16) | (-151.8, -54.12) |  |
| ∆ ≥15% | 5.7 | 0.0 | 6.5 |  |
| ∇ ≥15% | 91.4 | 92.9 | 89.6 |  |
|  |  |  |  |  |
| **RANTES** (median) | 0.00 | 0.00 | -0.17 | 0.847 |
| (P25, P75) | (-1.84, 1.12) | (-2.60, 0.88) | (-1.74, 0.45) |  |
| ∆ ≥15% | 0.0 | 0.0 | 0.0 |  |
| ∇ ≥15% | 0.0 | 0.0 | 0.0 |  |
|  |  |  |  |  |
| **EOTAXIN** (median) | -3.52 | 0.29 | -0.18 | 0.062 |
| (P25, P75) | (-5.53, 1.75) | (-4.47, 8.07) | (-4.71, 4.63) |  |
| ∆ ≥15% | 0.0 | 9.5 | 3.9 |  |
| ∇ ≥15% | 11.4 | 2.4 | 1.3 |  |
|  |  |  |  |  |
| **MIP-1α** (median) | 0.00 | 2.58 | 0.00 | 0.275 |
| (P25, P75) | (-17.06, 9.93) | (-5.33, 26.39) | (-7.59, 16.93) |  |
| ∆ ≥15% | 22.9 | 33.3 | 27.3 |  |
| ∇ ≥15% | 28.6 | 14.3 | 18.2 |  |
|  |  |  |  |  |
| **MIP-1β** (median) | 3.49 | 2.23 | 1.94 | 0.947 |
| (P25, P75) | (-4.75, 12.40) | (-2.44, 6.96) | (-4.38, 11.22) |  |
| ∆ ≥15% | 11.4 | 14.3 | 18.2 |  |
| ∇ ≥15% | 8.6 | 2.4 | 7.8 |  |
|  |  |  |  |  |

[ continued ]

**Supplemental Table 8b.** Continued

|  |  | | |  |
| --- | --- | --- | --- | --- |
| **Cytokine^a^** | **Primary or less** (N = 35) | **Secondary**  (N = 42) | **University**  (N = 77) | **P^b^** |
|  |  |  |  |  |
| **MCP-1** (median) | 0.31 | 1.06 | 1.03 | 0.890 |
| (P25, P75) | (-3.50, 4.88) | (-2.24, 4.46) | (-1.83, 3.33) |  |
| ∆ ≥15% | 2.9 | 0.0 | 0.0 |  |
| ∇ ≥15% | 2.9 | 0.0 | 1.3 |  |
|  |  |  |  |  |
| **MIG** (median) | 0.00 | 0.00 | 0.00 | 0.301 |
| (P25, P75) | (0.00, 0.00) | (0.00, 0.50) | (-11.45, 0.00) |  |
| ∆ ≥15% | 11.4 | 14.3 | 18.2 |  |
| ∇ ≥15% | 17.1 | 11.9 | 20.8 |  |
| **TH1** |  |  |  |  |
| **IL-2** (median) | 9.53 | 4.71 | 6.66 | 0.956 |
| (P25, P75) | (-19.05, 72.20) | (-13.36, 60.21) | (-16.86, 47.24) |  |
| ∆ ≥15% | 48.6 | 45.2 | 45.5 |  |
| ∇ ≥15% | 28.6 | 23.8 | 27.3 |  |
|  |  |  |  |  |
| **IL-12** (median) | 2.17 | 0.95 | 0.00 | 0.701 |
| (P25, P75) | (-6.25, 10.56) | (-5.14, 4.90) | (-6.22, 5.52) |  |
| ∆ ≥15% | 11.4 | 4.8 | 5.2 |  |
| ∇ ≥15% | 11.4 | 7.1 | 6.5 |  |
|  |  |  |  |  |
| **IFN-γ** (median) | 0.00 | 0.00 | 0.00 | 0.139 |
| (P25, P75) | (0.00, 0.00) | (0.00, 0.00) | (0.00, 0.00) |  |
| ∆ ≥15% | 14.3 | 7.1 | 9.1 |  |
| ∇ ≥15% | 2.9 | 4.8 | 13.0 |  |
| **TH2** |  |  |  |  |
| **IL-4** (median) | 0.00 | 0.00 | 0.00 | 0.434 |
| (P25, P75) | (0.00, 0.00) | (0.00, 1.98) | (0.00, 0.00) |  |
| ∆ ≥15% | 22.9 | 21.4 | 13.0 |  |
| ∇ ≥15% | 5.7 | 16.7 | 19.5 |  |
|  |  |  |  |  |
| **IL-5** (median) | 0.00 | 0.00 | 0.00 | 0.746 |
| (P25, P75) | (-84.04, 36.21) | (0.00, 12.22) | (-26.84, 63.19) |  |
| ∆ ≥15% | 25.7 | 21.4 | 29.9 |  |
| ∇ ≥15% | 25.7 | 9.5 | 26.0 |  |
|  |  |  |  |  |
| **IL-13** (median) | 0.00 | 0.00 | 0.00 | 0.500 |
| (P25, P75) | (-13.24, 43.30) | (-8.67, 35.02) | (-19.99, 20.51) |  |
| ∆ ≥15% | 28.6 | 33.3 | 26.0 |  |
| ∇ ≥15% | 22.9 | 21.4 | 26.0 |  |
| **Pro-inflammatory** |  |  |  |  |
| **IL-1β** (median) | 12.85 | 0.00 | 0.00 | 0.055 |
| (P25, P75) | (0.00, 51.78) | (-3.87, 40.73) | (-46.31, 27.43) |  |
| ∆ ≥15% | 45.7 | 35.7 | 28.6 |  |
| ∇ ≥15% | 11.4 | 23.8 | 35.1 |  |
|  |  |  |  |  |
| **TNF-α** (median) | 14.61 | 22.21 | 5.04 | 0.626 |
| (P25, P75) | (0.00, 71.29) | (0.00, 98.15) | (-12.14, 58.65) |  |
| ∆ ≥15% | 48.6 | 52.4 | 46.8 |  |
| ∇ ≥15% | 20.0 | 14.3 | 23.4 |  |
|  |  |  |  |  |
| **IL-6** (median) | 14.37 | 3.83 | 0.00 | 0.293 |
| (P25, P75) | (0.00, 46.86) | (-6.73, 39.18) | (-8.44, 30.92) |  |
| ∆ ≥15% | 45.7 | 42.9 | 33.8 |  |
| ∇ ≥15% | 11.4 | 19.0 | 18.2 |  |
|  |  |  |  |  |
| **IFN-α** (median) | 4.22 | 0.84 | 0.00 | 0.982 |
| (P25, P75) | (-11.80, 20.34) | (-8.35, 22.49) | (-9.02, 24.91) |  |
| ∆ ≥15% | 34.3 | 35.7 | 32.5 |  |
| ∇ ≥15% | 14.3 | 16.7 | 15.6 |  |
|  |  |  |  |  |

[ continued ]

**Supplemental Table 8b.** Continued

|  |  | | |  |
| --- | --- | --- | --- | --- |
| **Cytokine^a^** | **Primary or less** (N = 35) | **Secondary**  (N = 42) | **University**  (N = 77) | **P^b^** |
|  |  |  |  |  |
| **IL-2R** (median) | 7.23 | 2.43 | -0.71 | 0.186 |
| (P25, P75) | (-10.40, 19.53) | (-5.47, 14.04) | (-11.68, 11.06) |  |
| ∆ ≥15% | 34.2 | 23.8 | 16.9 |  |
| ∇ ≥15% | 17.1 | 9.5 | 19.5 |  |
|  |  |  |  |  |
| **IL-17** (median) | 0.00 | 0.00 | 0.00 | 0.533 |
| (P25, P75) | (0.00, 0.00) | (0.00, 41.97) | (-0.00, 20.38) |  |
| ∆ ≥15% | 22.9 | 31.0 | 26.0 |  |
| ∇ ≥15% | 14.3 | 14.3 | 22.1 |  |
| **Regulatory** |  |  |  |  |
| **IL-7** (median) | 0.00 | 0.00 | 0.00 | 0.881 |
| (P25, P75) | (-4.58, 16.01) | (-9.34, 18.55) | (-6.55, 13.69) |  |
| ∆ ≥15% | 25.7 | 26.2 | 23.4 |  |
| ∇ ≥15% | 17.1 | 21.4 | 16.9 |  |
| **Anti-inflammatory** |  |  |  |  |
| **IL-10** (median) | 0.00 | 0.00 | 0.00 | 0.738 |
| (P25, P75) | (-20.85, 67.45) | (-10.75, 2.70) | (-17.49, 34.28) |  |
| ∆ ≥15% | 34.3 | 21.4 | 32.5 |  |
| ∇ ≥15% | 28.6 | 23.8 | 24.7 |  |
|  |  |  |  |  |
| **IL-15** (median) | 0.00 | 0.00 | 0.00 | 0.861 |
| (P25, P75) | (0.00, 3.84) | (0.00, 0.14) | (0.00, 2.50) |  |
| ∆ ≥15% | 20.0 | 16.7 | 20.8 |  |
| ∇ ≥15% | 8.6 | 9.5 | 16.9 |  |
|  |  |  |  |  |
| **IL-1RA** (median) | -1.76 | -1.12 | -4.13 | 0.779 |
| (P25, P75) | (-5.85, 5.73) | (-11.90, 7.34) | (-9.60, 3.99) |  |
| ∆ ≥15% | 2.9 | 9.5 | 9.1 |  |
| ∇ ≥15% | 14.3 | 9.5 | 11.7 |  |
|  |  |  |  |  |

P25: percentile 25th. P75: percentile 75th.

Educational level groups are based on participant's data in 2020-21.

^a^ Units used for computing the relative intraindividual change for cytokine levels were pg/mL, base 10 logtransformed.

^b^ Kruskal-Wallis test (two-tailed).

**Supplemental Table 9a.** Concentrations of 24 isotype-antigen combinations for cytomegalovirus, Epstein-Barr and common cold infections, and of total Igs in 2016-17 by sex (N=240).

|  |  |  |  | |  |
| --- | --- | --- | --- | --- | --- |
| **Immunoglobulin**  (MFI) | **Total**  (N = 240) |  | **Men**  (N = 113) | **Women**  (N = 127) | **P^a^** |
| **IgA** |  |  |  |  |  |
| **IgA CMV pp150** (median) | 495.8 |  | 541.0 | 464.0 | 0.474 |
| (P25, P75) | (338.2, 830.4) |  | (339.5, 878.8) | (332.0, 758.5) |  |
| Geometric mean | 599.6 |  | 600.3 | 599.0 |  |
|  |  |  |  |  |  |
| **IgA CMV pp65** (median) | 579.5 |  | 589.0 | 570.5 | 0.990 |
| (P25, P75) | (411.4, 930.2) |  | (425.0, 929.5) | (404.0, 965.0) |  |
| Geometric mean | 690.2 |  | 682.7 | 697.0 |  |
|  |  |  |  |  |  |
| **IgA EBV EAD** (median) | 424.0 |  | 426.0 | 422.0 | 0.362 |
| (P25, P75) | (313.0, 640.2) |  | (320.0, 753.5) | (311.0, 611.0) |  |
| Geometric mean | 501.2 |  | 536.5 | 471.8 |  |
|  |  |  |  |  |  |
| **IgA VCAp18** (median) | 488.0 |  | 533.0 | 450.0 | 0.276 |
| (P25, P75) | (337.0, 753.8) |  | (340.5, 769.2) | (334.0, 696.5) |  |
| Geometric mean | 566.2 |  | 568.6 | 564.2 |  |
|  |  |  |  |  |  |
| **IgA N 229E** (median) | 1366 |  | 1330 | 1386 | 0.539 |
| (P25, P75) | (718.9, 3547) |  | (725.0, 4670) | (716.0, 3495) |  |
| Geometric mean | 1788 |  | 1961 | 1648 |  |
|  |  |  |  |  |  |
| **IgA N HKU1** (median) | 690.5 |  | 693.0 | 684.0 | 0.738 |
| (P25, P75) | (470.8, 1160) |  | (453.0, 1046) | (476.0, 1184) |  |
| Geometric mean | 816.7 |  | 799.6 | 832.2 |  |
|  |  |  |  |  |  |
| **IgA NL 63**  (median) | 1532 |  | 2078 | 1409 | 0.115 |
| (P25, P75) | (788.6, 4593) |  | (806.0, 6944) | (756.0, 3280) |  |
| Geometric mean | 2064 |  | 2394 | 1809 |  |
|  |  |  |  |  |  |
| **IgA N OC43** (median) | 1420 |  | 1528 | 1372 | 0.423 |
| (P25, P75) | (887.8, 2806) |  | (880.5, 3120) | (896.0, 2623) |  |
| Geometric mean | 1639 |  | 1746 | 1548 |  |
|  |  |  |  |  |  |

[ continued ]

**Supplemental Table 9a.** Continued.

|  |  |  |  | |  |
| --- | --- | --- | --- | --- | --- |
| **Immunoglobulin**  (MFI) | **Total**  (N = 240) |  | **Men**  (N = 113) | **Women**  (N = 127) | **P^a^** |
| **IgG** |  |  |  |  |  |
| **IgG CMV pp150** (median) | 15201 |  | 12441 | 18278 | 0.111 |
| (P25, P75) | (5334, 30751) |  | (4934, 26052) | (5496, 39185) |  |
| Geometric mean | 13591 |  | 12106 | 15065 |  |
|  |  |  |  |  |  |
| **IgG CMV pp65** (median) | 6819 |  | 6954 | 6658 | 0.918 |
| (P25, P75) | (4441, 10864) |  | (4783, 10402) | (4120, 11066) |  |
| Geometric mean | 6774 |  | 6766 | 6782 |  |
|  |  |  |  |  |  |
| **IgG EBV EAD** (median) | 6926 |  | 6858 | 6962 | 0.910 |
| (P25, P75) | (4760, 9991) |  | (4918, 9561) | (4748, 10196) |  |
| Geometric mean | 6846 |  | 6806 | 6881 |  |
|  |  |  |  |  |  |
| **IgG VCAp18** (median) | 7810 |  | 7400 | 8092 | 0.799 |
| (P25, P75) | (4280, 14308) |  | (4281, 14284) | (4274, 14419) |  |
| Geometric mean | 8528 |  | 8869 | 8236 |  |
|  |  |  |  |  |  |
| **IgG N 229E** (median) | 44429 |  | 52588 | 36615 | 0.009 |
| (P25, P75) | (25747, 73639) |  | (28715, 77569) | (22440, 68493) |  |
| Geometric mean | 41828 |  | 47429 | 37403 |  |
|  |  |  |  |  |  |
| **IgG N HKU1** (median) | 14094 |  | 13620 | 14351 | 0.681 |
| (P25, P75) | (10031, 18800) |  | (9967, 18550) | (10087, 19685) |  |
| Geometric mean | 13710 |  | 13749 | 13675 |  |
|  |  |  |  |  |  |
| **IgG NL 63**  (median) | 36644 |  | 39669 | 32016 | 0.166 |
| (P25, P75) | (20923, 58452) |  | (22704, 58558) | (19408, 58443) |  |
| Geometric mean | 34243 |  | 36698 | 32197 |  |
|  |  |  |  |  |  |
| **IgG N OC43** (median) | 21454 |  | 21540 | 21367 | 0.314 |
| (P25, P75) | (11238, 37532) |  | (11454, 42493) | (10888, 34667) |  |
| Geometric mean | 21072 |  | 22278 | 20054 |  |
|  |  |  |  |  |  |

[ continued ]

**Supplemental Table 9a.** Continued.

|  |  |  |  | |  |
| --- | --- | --- | --- | --- | --- |
| **Immunoglobulin**  (MFI) | **Total**  (N = 240) |  | **Men**  (N = 113) | **Women**  (N = 127) | **P^a^** |
| **IgM** |  |  |  |  |  |
| **IgM CMV pp150** (median) | 1277 |  | 1020 | 1596 | <0.001 |
| (P25, P75) | (724.4, 2420) |  | (564.8, 1604) | (930.5, 3382) |  |
| Geometric mean | 1378 |  | 1060 | 1742 |  |
|  |  |  |  |  |  |
| **IgM CMV pp65** (median) | 655.0 |  | 517.5 | 804.0 | <0.001 |
| (P25, P75) | (427.6, 1036) |  | (398.2, 809.5) | (525.5, 1155) |  |
| Geometric mean | 698.2 |  | 577.3 | 826.8 |  |
|  |  |  |  |  |  |
| **IgM EBV EAD** (median) | 423.8 |  | 363.5 | 488.0 | <0.001 |
| (P25, P75) | (304.2, 590.5) |  | (268.5, 506.0) | (338.0, 680.0) |  |
| Geometric mean | 435.8 |  | 377.8 | 494.7 |  |
|  |  |  |  |  |  |
| **IgM VCAp18** (median) | 861.2 |  | 677.0 | 1020 | <0.001 |
| (P25, P75) | (498.8, 1599) |  | (442.2, 1118) | (592.0, 1872) |  |
| Geometric mean | 911.1 |  | 749.3 | 1084 |  |
|  |  |  |  |  |  |
| **IgM N 229E** (median) | 1431 |  | 1162 | 1680 | <0.001 |
| (P25, P75) | (863.6, 2378) |  | (707.0, 1708) | (1189, 3009) |  |
| Geometric mean | 1444 |  | 1140 | 1783 |  |
|  |  |  |  |  |  |
| **IgM N HKU1** (median) | 1022 |  | 850.5 | 1147 | <0.001 |
| (P25, P75) | (705.6, 1652) |  | (567.5, 1360) | (866.5, 1757) |  |
| Geometric mean | 1079 |  | 922.6 | 1239 |  |
|  |  |  |  |  |  |
| **IgM NL 63**  (median) | 948.2 |  | 697.5 | 1249 | <0.001 |
| (P25, P75) | (591.5, 1568) |  | (448.2, 1104) | (783.0, 1918) |  |
| Geometric mean | 1012 |  | 764.0 | 1298 |  |
|  |  |  |  |  |  |
| **IgM N OC43** (median) | 2060 |  | 1510 | 2664 | <0.001 |
| (P25, P75) | (1121, 3496) |  | (991.2, 2569) | (1288, 3948) |  |
| Geometric mean | 2065 |  | 1630 | 2547 |  |
|  |  |  |  |  |  |

[ continued ]

**Supplemental Table 9a.** Continued.

|  |  |  |  | |  |
| --- | --- | --- | --- | --- | --- |
| **Immunoglobulin**  (µg/mL) | **Total**  (N = 240) |  | **Men**  (N = 113) | **Women**  (N = 127) | **P^a^** |
| **Total immunoglobulin** |  |  |  |  |  |
| **IgG1** (median) | 6111 |  | 5777 | 6648 | 0.029 |
| (P25, P75) | (4744, 8220) |  | (4410, 7388) | (5108, 8403) |  |
| Geometric mean | 6250 |  | 5917 | 6561 |  |
|  |  |  |  |  |  |
| **IgG2** (median) | 3119 |  | 2857 | 3206 | 0.005 |
| (P25, P75) | (2486, 3690) |  | (2380, 3544) | (2595, 3783) |  |
| Geometric mean | 3040 |  | 2867 | 3202 |  |
|  |  |  |  |  |  |
| **IgG3** (median) | 1237 |  | 1158 | 1294 | 0.015 |
| (P25, P75) | (1004, 1472) |  | (960.4, 1413) | (1073, 1531) |  |
| Geometric mean | 1198 |  | 1134 | 1258 |  |
|  |  |  |  |  |  |
| **IgG4** (median) | 228.7 |  | 225.1 | 231.6 | 0.628 |
| (P25, P75) | (122.7, 400.8) |  | (129.1, 407.6) | (107.6, 396.8) |  |
| Geometric mean | 226.7 |  | 234.7 | 219.9 |  |
|  |  |  |  |  |  |
| **Sum of IgGs** (median) | 10900 |  | 10447 | 11426 | 0.010 |
| (P25, P75) | (8865, 13719) |  | (8474, 12684) | (9425, 14123) |  |
| Geometric mean | 11035 |  | 10479 | 11555 |  |
|  |  |  |  |  |  |
| **IgE** (median) | 0.23 |  | 0.24 | 0.21 | 0.242 |
| (P25, P75) | (0.15, 0.33) |  | (0.15, 0.35) | (0.14, 0.32) |  |
| Geometric mean | 0.23 |  | 0.24 | 0.22 |  |
|  |  |  |  |  |  |
| **IgA** (median) | 246.1 |  | 244.9 | 247.1 | 0.418 |
| (P25, P75) | (193.7, 301.7) |  | (185.2, 293.5) | (196.2, 313.2) |  |
| Geometric mean | 239.9 |  | 233.9 | 245.5 |  |
|  |  |  |  |  |  |
| **IgM** (median) | 918.7 |  | 809.7 | 1158 | <0.001 |
| (P25, P75) | (690.0, 1325) |  | (654.1, 986.9) | (773.3, 1480) |  |
| Geometric mean | 946.7 |  | 806.7 | 1091 |  |
|  |  |  |  |  |  |

P25: percentile 25th. P75: percentile 75th.

<LOQ: value lower than the minimum concentration of the corresponding range of limits of quantification.

See Supplemental Table 1.

^a^ Mann-Whitney’s *U* test (two-tail).

**Supplemental Table 9b.** Relative intraindividual change (%) from 2016-17 to 2020-21 of concentrations of 24 isotype-antigen combinations for cytomegalovirus, Epstein-Barr and common cold infections, and of total Igs, by sex (N=154).

|  |  | |  |
| --- | --- | --- | --- |
| **Immunoglobulin^a^** | **Men**  (N = 72) | **Women**  (N = 82) | **P^b^** |
| **IgA** |  |  |  |
| **IgA CMV pp150** (median) | 0.36 | 0.48 | 0.735 |
| (P25, P75) | (-2.17, 4.47) | (-2.81, 3.15) |  |
| ∆ ≥15% | 4.2 | 4.9 |  |
| ∇ ≥15% | 0.0 | 1.2 |  |
|  |  |  |  |
| **IgA CMV pp65** (median) | 0.66 | 1.06 | 0.648 |
| (P25, P75) | (-2.29, 4.61) | (-2.33, 3.27) |  |
| ∆ ≥15% | 2.8 | 1.2 |  |
| ∇ ≥15% | 0.0 | 1.2 |  |
|  |  |  |  |
| **IgA EBV EAD** (median) | -0.16 | 0.74 | 0.957 |
| (P25, P75) | (-2.64, 4.10) | (-1.99, 2.82) |  |
| ∆ ≥15% | 2.8 | 0.0 |  |
| ∇ ≥15% | 1.4 | 1.2 |  |
|  |  |  |  |
| **IgA VCAp18** (median) | 0.45 | 1.05 | 0.991 |
| (P25, P75) | (-2.08, 3.62) | (-2.16, 3.48) |  |
| ∆ ≥15% | 4.2 | 0.0 |  |
| ∇ ≥15% | 0.0 | 1.2 |  |
|  |  |  |  |
| **IgA N 229E** (median) | 0.28 | -0.93 | 0.250 |
| (P25, P75) | (-4.05, 5.01) | (-3.74, 2.84) |  |
| ∆ ≥15% | 5.6 | 0.0 |  |
| ∇ ≥15% | 5.6 | 4.9 |  |
|  |  |  |  |
| **IgA N HKU1** (median) | 1.51 | 0.70 | 0.393 |
| (P25, P75) | (-2.11, 3.41) | (-2.68, 2.72) |  |
| ∆ ≥15% | 4.2 | 1.2 |  |
| ∇ ≥15% | 0.0 | 0.0 |  |
|  |  |  |  |
| **IgA NL 63** (median) | -0.55 | -0.19 | 0.761 |
| (P25, P75) | (-3.79, 6.79) | (-3.55, 3.65) |  |
| ∆ ≥15% | 9.7 | 3.7 |  |
| ∇ ≥15% | 2.8 | 6.1 |  |
|  |  |  |  |
| **IgA N OC43** (median) | 0.03 | 0.39 | 0.797 |
| (P25, P75) | (-2.84, 3.37) | (-2.60, 2.91) |  |
| ∆ ≥15% | 1.4 | 3.7 |  |
| ∇ ≥15% | 0.0 | 1.2 |  |
|  |  |  |  |

[ continued ]

**Supplemental Table 9b.** Continued

|  |  | |  |
| --- | --- | --- | --- |
| **Immunoglobulin^a^** | **Men**  (N = 72) | **Women**  (N = 82) | **P^b^** |
| **IgG** |  |  |  |
| **IgG CMV pp150** (median) | 1.57 | 1.31 | 0.744 |
| (P25, P75) | (-1.69, 3.99) | (-1.04, 4.04) |  |
| ∆ ≥15% | 2.8 | 1.2 |  |
| ∇ ≥15% | 0.0 | 0.0 |  |
|  |  |  |  |
| **IgG CMV pp65** (median) | 1.51 | 0.31 | 0.222 |
| (P25, P75) | (-1.33, 3.91) | (-2.88, 2.79) |  |
| ∆ ≥15% | 0.0 | 1.2 |  |
| ∇ ≥15% | 0.0 | 0.0 |  |
|  |  |  |  |
| **IgG EBV EAD** (median) | 0.85 | -0.22 | 0.659 |
| (P25, P75) | (-2.27, 2.56) | (-2.20, 3.24) |  |
| ∆ ≥15% | 0.0 | 0.0 |  |
| ∇ ≥15% | 0.0 | 0.0 |  |
|  |  |  |  |
| **IgG VCAp18** (median) | 0.87 | 0.33 | 0.232 |
| (P25, P75) | (-1.79, 4.17) | (-2.17, 3.13) |  |
| ∆ ≥15% | 2.8 | 1.2 |  |
| ∇ ≥15% | 0.0 | 1.2 |  |
|  |  |  |  |
| **IgG N 229E** (median) | 0.15 | 0.15 | 0.888 |
| (P25, P75) | (-2.56, 2.77) | (-2.56, 2.70) |  |
| ∆ ≥15% | 1.4 | 0.0 |  |
| ∇ ≥15% | 0.0 | 0.0 |  |
|  |  |  |  |
| **IgG N HKU1** (median) | 1.15 | 0.23 | 0.244 |
| (P25, P75) | (-1.37, 2.48) | (-1.63, 2.39) |  |
| ∆ ≥15% | 0.0 | 0.0 |  |
| ∇ ≥15% | 0.0 | 0.0 |  |
|  |  |  |  |
| **IgG NL 63** (median) | 0.36 | -0.35 | 0.199 |
| (P25, P75) | (-2.57, 3.58) | (-2.48, 2.26) |  |
| ∆ ≥15% | 0.0 | 0.0 |  |
| ∇ ≥15% | 0.0 | 0.0 |  |
|  |  |  |  |
| **IgG N OC43** (median) | 0.36 | -0.24 | 0.187 |
| (P25, P75) | (-2.21, 3.97) | (-4.18, 2.65) |  |
| ∆ ≥15% | 0.0 | 1.2 |  |
| ∇ ≥15% | 1.4 | 3.7 |  |
|  |  |  |  |

[ continued ]

**Supplemental Table 9b.** Continued

|  |  | |  |  |
| --- | --- | --- | --- | --- |
| **Immunoglobulin^a^** | **Men**  (N = 72) | **Women**  (N = 82) | **P^b^** |  |
| **IgM** |  |  |  |  |
| **IgM CMV pp150** (median) | 1.08 | 1.01 | 0.415 |  |
| (P25, P75) | (-1.58, 5.13) | (-2.32, 3.36) |  |  |
| ∆ ≥15% | 2.8 | 0.0 |  |  |
| ∇ ≥15% | 1.4 | 0.0 |  |  |
|  |  |  |  |  |
| **IgM CMV pp65** (median) | 0.78 | 0.44 | 0.853 |  |
| (P25, P75) | (-2.02, 2.82) | (-1.59, 4.04) |  |  |
| ∆ ≥15% | 1.4 | 0.0 |  |  |
| ∇ ≥15% | 0.0 | 0.0 |  |  |
|  |  |  |  |  |
| **IgM EBV EAD** (median) | 0.73 | 0.14 | 0.245 |  |
| (P25, P75) | (-1.68, 3.76) | (-2.99, 3.31) |  |  |
| ∆ ≥15% | 2.8 | 0.0 |  |  |
| ∇ ≥15% | 0.0 | 0.0 |  |  |
|  |  |  |  |  |
| **IgM VCAp18** (median) | 1.72 | 0.43 | 0.107 |  |
| (P25, P75) | (-1.72, 5.21) | (-2.57, 3.79) |  |  |
| ∆ ≥15% | 1.4 | 2.4 |  |  |
| ∇ ≥15% | 0.0 | 0.0 |  | |
|  |  |  |  | |
| **IgM N 229E** (median) | 1.85 | -0.16 | 0.046 | |
| (P25, P75) | (-1.24, 5.42) | (-3.28, 3.97) |  | |
| ∆ ≥15% | 4.2 | 1.2 |  | |
| ∇ ≥15% | 0.0 | 1.2 |  | |
|  |  |  |  | |
| **IgM N HKU1** (median) | 1.34 | 0.71 | 0.407 | |
| (P25, P75) | (-1.59, 4.38) | (-1.42, 2.82) |  | |
| ∆ ≥15% | 2.8 | 1.2 |  | |
| ∇ ≥15% | 0.0 | 0.0 |  | |
|  |  |  |  | |
| **IgM NL 63** (median) | 1.56 | 0.63 | 0.221 | |
| (P25, P75) | (-2.20, 4.23) | (-3.92, 5.56) |  | |
| ∆ ≥15% | 2.8 | 0.0 |  | |
| ∇ ≥15% | 1.4 | 0.0 |  | |
|  |  |  |  | |
| **IgM N OC43** (median) | 0.27 | 1.26 | 0.567 | |
| (P25, P75) | (-2.68, 4.23) | (-2.53, 4.35) |  | |
| ∆ ≥15% | 1.4 | 2.4 |  | |
| ∇ ≥15% | 0.0 | 0.0 |  | |
|  |  |  |  |  |

[ continued ]

**Supplemental Table 9b.** Continued

|  |  | |  |  |
| --- | --- | --- | --- | --- |
| **Immunoglobulin^a^** | **Men**  (N = 72) | **Women**  (N = 82) | **P^b^** |  |
| **Total immunoglobulin** |  |  |  |  |
| **IgG1**  (median) | 1.91 | 0.62 | 0.025 |  |
| (P25, P75) | (-0.83, 3.58) | (-2.22, 2.32) |  |  |
| ∆ ≥15% | 0.0 | 0.0 |  |  |
| ∇ ≥15% | 0.0 | 0.0 |  |  |
|  |  |  |  |  |
| **IgG2** (median) | 0.88 | 0.46 | 0.087 |  |
| (P25, P75) | (-0.26, 2.38) | (-0.86, 1.88) |  |  |
| ∆ ≥15% | 0.0 | 0.0 |  |  |
| ∇ ≥15% | 0.0 | 0.0 |  |  |
|  |  |  |  |  |
| **IgG3** (median) | -0.01 | -0.80 | 0.297 |  |
| (P25, P75) | (-2.65, 1.77) | (-2.65, 1.09) |  |  |
| ∆ ≥15% | 0.0 | 0.0 |  |  |
| ∇ ≥15% | 0.0 | 0.0 |  |  |
|  |  |  |  |  |
| **IgG4** (median) | 0.96 | 1.17 | 0.640 |  |
| (P25, P75) | (-2.27, 4.59) | (-1.42, 4.27) |  |  |
| ∆ ≥15% | 2.8 | 0.0 |  |  |
| ∇ ≥15% | 1.4 | 0.0 |  | |
|  |  |  |  | |
| **Sum of IgGs** (median) | 1.20 | 0.26 | 0.014 | |
| (P25, P75) | (-0.46, 2.79) | (-1.63, 1.98) |  | |
| ∆ ≥15% | 0.0 | 0.0 |  | |
| ∇ ≥15% | 0.0 | 0.0 |  | |
|  |  |  |  | |
| **IgE** (median) | 4.11 | 1.39 | 0.262 | |
| (P25, P75) | (-4.47, 13.02) | (-3.65, 9.36) |  | |
| ∆ ≥15% | 20.8 | 13.4 |  | |
| ∇ ≥15% | 8.3 | 9.8 |  | |
|  |  |  |  | |
| **IgA** (median) | 1.17 | 0.29 | 0.074 | |
| (P25, P75) | (-1.32, 4.60) | (-2.81, 3.61) |  | |
| ∆ ≥15% | 1.4 | 0.0 |  | |
| ∇ ≥15% | 0.0 | 0.0 |  | |
|  |  |  |  | |
| **IgM** (median) | 1.05 | -0.25 | 0.069 | |
| (P25, P75) | (-1.05, 3.53) | (-1.89, 2.82) |  | |
| ∆ ≥15% | 0.0 | 0.0 |  | |
| ∇ ≥15% | 0.0 | 0.0 |  | |
|  |  |  |  |  |

P25: percentile 25th. P75: percentile 75th.

^a^ Units used for computing the relative intraindividual change were base 10 logtransformed MFI for levels of isotype-antigen combinations, and base 10 logtransformed µg/mL for levels of total Igs.

^b^ Mann-Whitney’s *U* test (two-tail).

**Supplemental Table 10a.** Concentrations of 24 isotype-antigen combinations for cytomegalovirus, Epstein-Barr and common cold infections, and of total Igs in 2016-17 by age group (N=240).

|  |  |  |  |  |
| --- | --- | --- | --- | --- |
| **Immunoglobulin**  (MFI) | **<45 years**  (N = 88) | **45 – 64 years**  (N = 105) | **≥65 years**  (N = 47) | **P^a^** |
| **IgA** |  |  |  |  |
| **IgA CMV pp150** (median) | 488.5 | 439.0 | 677.5 | 0.029 |
| (P25, P75) | (330.1, 826.8) | (329.8, 696.2) | (427.0, 1063) |  |
| Geometric mean | 586.5 | 558.4 | 732.5 |  |
|  |  |  |  |  |
| **IgA CMV pp65** (median) | 523.0 | 569.0 | 802.5 | 0.005 |
| (P25, P75) | (397.2, 851.0) | (397.2, 792.0) | (466.0, 1702) |  |
| Geometric mean | 592.9 | 664.4 | 999.0 |  |
|  |  |  |  |  |
| **IgA EBV EAD** (median) | 372.8 | 407.0 | 580.0 | 0.029 |
| (P25, P75) | (305.4, 579.8) | (308.5, 631.8) | (330.0, 1244) |  |
| Geometric mean | 439.2 | 499.9 | 645.8 |  |
|  |  |  |  |  |
| **IgA VCAp18** (median) | 447.0 | 488.5 | 590.0 | 0.096 |
| (P25, P75) | (328.1, 680.5) | (351.0, 734.2) | (332.0, 973.0) |  |
| Geometric mean | 490.4 | 591.5 | 672.3 |  |
|  |  |  |  |  |
| **IgA N 229E** (median) | 1290 | 1558 | 1546 | 0.651 |
| (P25, P75) | (727.8, 3471) | (716.5, 3986) | (717.5, 3504) |  |
| Geometric mean | 1607 | 1819 | 2105 |  |
|  |  |  |  |  |
| **IgA N HKU1** (median) | 629.0 | 684.0 | 932.0 | 0.031 |
| (P25, P75) | (441.0, 1031) | (478.2, 1094) | (476.0, 1706) |  |
| Geometric mean | 707.2 | 824.9 | 1046 |  |
|  |  |  |  |  |
| **IgA NL 63**  (median) | 1635 | 1345 | 2285 | 0.329 |
| (P25, P75) | (785.4, 4157) | (772.2, 4336) | (793.0, 8840) |  |
| Geometric mean | 1949 | 1905 | 2750 |  |
|  |  |  |  |  |
| **IgA N OC43** (median) | 1382 | 1377 | 1544 | 0.830 |
| (P25, P75) | (899.2, 2679) | (835.2, 2725) | (913.0, 3345) |  |
| Geometric mean | 1562 | 1600 | 1891 |  |
|  |  |  |  |  |

[ continued ]

**Supplemental Table 10a.** Continued.

|  |  |  |  |  |
| --- | --- | --- | --- | --- |
| **Immunoglobulin**  (MFI) | **<45 years**  (N = 88) | **45 – 64 years**  (N = 105) | **≥65 years**  (N = 47) | **P^a^** |
| **IgG** |  |  |  |  |
| **IgG CMV pp150** (median) | 13886 | 14982 | 19973 | 0.226 |
| (P25, P75) | (5481, 27945) | (5107, 29360) | (5937, 46896) |  |
| Geometric mean | 12606 | 12813 | 17854 |  |
|  |  |  |  |  |
| **IgG CMV pp65** (median) | 7754 | 6572 | 6756 | 0.099 |
| (P25, P75) | (5060, 12612) | (4068, 9177) | (3615, 11066) |  |
| Geometric mean | 7559 | 6321 | 6440 |  |
|  |  |  |  |  |
| **IgG EBV EAD** (median) | 7287 | 7007 | 5914 | 0.388 |
| (P25, P75) | (4976, 10318) | (4714, 9310) | (4630, 9082) |  |
| Geometric mean | 7145 | 6825 | 6363 |  |
|  |  |  |  |  |
| **IgG VCAp18** (median) | 8100 | 7815 | 7066 | 0.620 |
| (P25, P75) | (4338, 13503) | (4300, 14532) | (3334, 14772) |  |
| Geometric mean | 8139 | 9105 | 8041 |  |
|  |  |  |  |  |
| **IgG N 229E** (median) | 46607 | 43472 | 42804 | 0.717 |
| (P25, P75) | (24041, 76984) | (28790, 70706) | (23096, 72216) |  |
| Geometric mean | 41710 | 43992 | 37568 |  |
|  |  |  |  |  |
| **IgG N HKU1** (median) | 13666 | 14345 | 12973 | 0.671 |
| (P25, P75) | (9492, 19883) | (10446, 19091) | (10408, 16628) |  |
| Geometric mean | 13290 | 14347 | 13129 |  |
|  |  |  |  |  |
| **IgG NL 63**  (median) | 41605 | 31820 | 33123 | 0.124 |
| (P25, P75) | (24773, 63705) | (22243, 57049) | (17438, 50410) |  |
| Geometric mean | 38088 | 33284 | 29893 |  |
|  |  |  |  |  |
| **IgG N OC43** (median) | 24018 | 19178 | 21886 | 0.451 |
| (P25, P75) | (11440, 38243) | (10664, 36361) | (13010, 40084) |  |
| Geometric mean | 21870 | 19811 | 22560 |  |
|  |  |  |  |  |

[ continued ]

**Supplemental Table 10a.** Continued.

|  |  |  |  |  |
| --- | --- | --- | --- | --- |
| **Immunoglobulin**  (MFI) | **<45 years**  (N = 88) | **45 – 64 years**  (N = 105) | **≥65 years**  (N = 47) | **P^a^** |
| **IgM** |  |  |  |  |
| **IgM CMV pp150** (median) | 1512 | 1130 | 997.0 | 0.005 |
| (P25, P75) | (923.8, 3278) | (702.2, 2064) | (527.0, 1754) |  |
| Geometric mean | 1692 | 1275 | 1117 |  |
|  |  |  |  |  |
| **IgM CMV pp65** (median) | 818.8 | 605.0 | 446.5 | <0.001 |
| (P25, P75) | (574.5, 1356) | (404.2, 919.2) | (351.0, 718.0) |  |
| Geometric mean | 905.4 | 628.6 | 542.7 |  |
|  |  |  |  |  |
| **IgM EBV EAD** (median) | 475.8 | 424.0 | 338.0 | 0.011 |
| (P25, P75) | (328.0, 657.8) | (300.5, 546.0) | (250.0, 496.0) |  |
| Geometric mean | 482.6 | 431.5 | 367.8 |  |
|  |  |  |  |  |
| **IgM VCAp18** (median) | 1176 | 805.0 | 510.0 | <0.001 |
| (P25, P75) | (747.6, 1934) | (495.0, 1450) | (359.5, 870.5) |  |
| Geometric mean | 1226 | 868.4 | 582.1 |  |
|  |  |  |  |  |
| **IgM N 229E** (median) | 1608 | 1402 | 1094 | 0.002 |
| (P25, P75) | (1234, 2688) | (730.0, 2185) | (682.0, 2311) |  |
| Geometric mean | 1762 | 1336 | 1185 |  |
|  |  |  |  |  |
| **IgM N HKU1** (median) | 1242 | 932.0 | 919.0 | 0.002 |
| (P25, P75) | (846.6, 1831) | (638.5, 1474) | (601.0, 1522) |  |
| Geometric mean | 1261 | 1021 | 910.8 |  |
|  |  |  |  |  |
| **IgM NL 63**  (median) | 1157 | 783.0 | 778.5 | <0.001 |
| (P25, P75) | (785.0, 1980) | (502.0, 1381) | (509.0, 1203) |  |
| Geometric mean | 1292 | 895.2 | 841.0 |  |
|  |  |  |  |  |
| **IgM N OC43** (median) | 2313 | 1980 | 1796 | 0.062 |
| (P25, P75) | (1439, 3708) | (988.8, 3545) | (992.0, 3074) |  |
| Geometric mean | 2363 | 1986 | 1748 |  |
|  |  |  |  |  |

[ continued ]

**Supplemental Table 10a.** Continued.

|  |  |  |  |  |
| --- | --- | --- | --- | --- |
| **Immunoglobulin**  (µg/mL) | **<45 years**  (N = 88) | **45 – 64 years**  (N = 105) | **≥65 years**  (N = 47) | **P^a^** |
| **Total immunoglobulin** |  |  |  |  |
| **IgG1** (median) | 6671 | 5777 | 5993 | 0.113 |
| (P25, P75) | (5063, 8957) | (4536, 7943) | (4624, 8412) |  |
| Geometric mean | 6699 | 5902 | 6236 |  |
|  |  |  |  |  |
| **IgG2** (median) | 3200 | 3055 | 3098 | 0.168 |
| (P25, P75) | (2659, 3741) | (2413, 3579) | (2376, 3732) |  |
| Geometric mean | 3201 | 2905 | 3053 |  |
|  |  |  |  |  |
| **IgG3** (median) | 1236 | 1251 | 1198 | 0.767 |
| (P25, P75) | (1012, 1519) | (989.8, 1488) | (1055, 1406) |  |
| Geometric mean | 1232 | 1186 | 1164 |  |
|  |  |  |  |  |
| **IgG4** (median) | 270.3 | 199.7 | 253.2 | 0.047 |
| (P25, P75) | (138.8, 479.4) | (103.4, 351.8) | (134.6, 398.6) |  |
| Geometric mean | 264.0 | 196.8 | 234.1 |  |
|  |  |  |  |  |
| **Sum of IgGs** (median) | 11466 | 10344 | 10891 | 0.033 |
| (P25, P75) | (9775, 14612) | (8433, 12781) | (9094, 13705) |  |
| Geometric mean | 11730 | 10482 | 11041 |  |
|  |  |  |  |  |
| **IgE** (median) | 0.26 | 0.20 | 0.24 | 0.041 |
| (P25, P75) | (0.18, 0.35) | (0.14, 0.31) | (0.17, 0.35) |  |
| Geometric mean | 0.25 | 0.21 | 0.24 |  |
|  |  |  |  |  |
| **IgA** (median) | 238.2 | 249.6 | 246.8 | 0.736 |
| (P25, P75) | (194.2, 300.2) | (192.8, 304.1) | (188.5, 307.2) |  |
| Geometric mean | 234.2 | 240.5 | 249.7 |  |
|  |  |  |  |  |
| **IgM** (median) | 1056 | 885.1 | 816.5 | 0.005 |
| (P25, P75) | (777.9, 1347) | (635.1, 1407) | (612.5, 1151) |  |
| Geometric mean | 1054 | 926.2 | 812.9 |  |
|  |  |  |  |  |

P25: percentile 25th. P75: percentile 75th.

<LOQ: value lower than the minimum concentration of the corresponding range of limits of quantification.

See Supplemental Table 1.

^a^ Kruskal-Wallis test (two-tailed).

**Supplemental Table 10b.** Relative intraindividual change (%) from 2016-17 to 2020-21 of concentrations of 24 isotype-antigen combinations for cytomegalovirus, Epstein-Barr and common cold infections, and of total Igs, by age group (N=154).

|  |  | | |  |
| --- | --- | --- | --- | --- |
| **Immunoglobulin^a^** | **<46 years**  (N = 51) | **46 – 62 years**  (N = 49) | **≥63 years**  (N = 54) | **P^b^** |
| **IgA** |  |  |  |  |
| **IgA CMV pp150** (median) | 0.62 | 0.55 | 0.25 | 0.326 |
| (P25, P75) | (-1.99, 5.97) | (-2.77, 3.16) | (-2.85, 2.87) |  |
| ∆ ≥15% | 11.8 | 0.0 | 1.9 |  |
| ∇ ≥15% | 0.0 | 0.0 | 1.9 |  |
|  |  |  |  |  |
| **IgA CMV pp65** (median) | 0.47 | 1.10 | 0.88 | 0.793 |
| (P25, P75) | (-2.86, 3.39) | (-2.06, 2.57) | (-2.18, 5.33) |  |
| ∆ ≥15% | 3.9 | 0.0 | 1.9 |  |
| ∇ ≥15% | 0.0 | 0.0 | 1.9 |  |
|  |  |  |  |  |
| **IgA EBV EAD** (median) | -0.09 | 0.63 | 0.74 | 0.998 |
| (P25, P75) | (-2.37, 3.84) | (-1.96, 3.44) | (-2.84, 3.98) |  |
| ∆ ≥15% | 3.9 | 0.0 | 0.0 |  |
| ∇ ≥15% | 0.0 | 2.0 | 1.9 |  |
|  |  |  |  |  |
| **IgA VCAp18** (median) | 0.54 | 1.12 | 0.45 | 0.808 |
| (P25, P75) | (-2.07, 2.96) | (-2.02, 4.62) | (-2.55, 3.46) |  |
| ∆ ≥15% | 3.9 | 2.0 | 0.0 |  |
| ∇ ≥15% | 0.0 | 0.0 | 1.9 |  |
|  |  |  |  |  |
| **IgA N 229E** (median) | -0.02 | -0.23 | -0.76 | 0.929 |
| (P25, P75) | (-3.32, 3.50) | (-4.98, 3.14) | (-3.90, 5.12) |  |
| ∆ ≥15% | 2.0 | 6.1 | 0.0 |  |
| ∇ ≥15% | 7.8 | 2.0 | 5.6 |  |
|  |  |  |  |  |
| **IgA N HKU1** (median) | 0.80 | 0.94 | 1.02 | 0.550 |
| (P25, P75) | (-1.69, 2.31) | (-2.67, 2.76) | (-1.86, 3.73) |  |
| ∆ ≥15% | 5.9 | 0.0 | 1.9 |  |
| ∇ ≥15% | 0.0 | 0.0 | 0.0 |  |
|  |  |  |  |  |
| **IgA NL 63** (median) | -0.32 | -0.41 | -0.53 | 0.780 |
| (P25, P75) | (-3.98, 6.09) | (-2.18, 3.65) | (-5.44, 4.88) |  |
| ∆ ≥15% | 7.8 | 8.2 | 3.7 |  |
| ∇ ≥15% | 3.9 | 4.1 | 5.6 |  |
|  |  |  |  |  |
| **IgA N OC43** (median) | -0.36 | 0.37 | 0.92 | 0.958 |
| (P25, P75) | (-2.86, 3.62) | (-2.46, 3.69) | (-2.73, 2.58) |  |
| ∆ ≥15% | 5.9 | 0.0 | 1.9 |  |
| ∇ ≥15% | 0.0 | 0.0 | 1.9 |  |
|  |  |  |  |  |

[ continued ]

**Supplemental Table 10b.** Continued

|  |  | | |  |
| --- | --- | --- | --- | --- |
| **Immunoglobulin^a^** | **<46 years**  (N = 51) | **46 – 62 years**  (N = 49) | **≥63 years**  (N = 54) | **P^b^** |
| **IgG** |  |  |  |  |
| **IgG CMV pp150** (median) | 2.25 | 0.83 | 1.12 | 0.717 |
| (P25, P75) | (-1.50, 3.99) | (-2.06, 3.98) | (-0.72, 4.13) |  |
| ∆ ≥15% | 5.9 | 0.0 | 0.0 |  |
| ∇ ≥15% | 0.0 | 0.0 | 0.0 |  |
|  |  |  |  |  |
| **IgG CMV pp65** (median) | 0.46 | 0.82 | 0.95 | 0.565 |
| (P25, P75) | (-3.18, 2.51) | (-1.82, 2.92) | (-1.92, 3.95) |  |
| ∆ ≥15% | 0.0 | 0.0 | 1.9 |  |
| ∇ ≥15% | 0.0 | 0.0 | 0.0 |  |
|  |  |  |  |  |
| **IgG EBV EAD** (median) | 0.97 | 0.80 | -0.21 | 0.278 |
| (P25, P75) | (-2.97, 3.17) | (-1.12, 3.37) | (-2.89, 2.17) |  |
| ∆ ≥15% | 0.0 | 0.0 | 0.0 |  |
| ∇ ≥15% | 0.0 | 0.0 | 0.0 |  |
|  |  |  |  |  |
| **IgG VCAp18** (median) | 0.62 | 0.67 | 0.73 | 0.836 |
| (P25, P75) | (-3.19, 3.18) | (-1.25, 2.88) | (-2.35, 3.47) |  |
| ∆ ≥15% | 2.0 | 2.0 | 1.9 |  |
| ∇ ≥15% | 2.0 | 0.0 | 0.0 |  |
|  |  |  |  |  |
| **IgG N 229E** (median) | 0.15 | -0.18 | 0.35 | 0.886 |
| (P25, P75) | (-2.91, 2.71) | (-2.35, 2.35) | (-2.53, 3.03) |  |
| ∆ ≥15% | 2.0 | 0.0 | 0.0 |  |
| ∇ ≥15% | 0.0 | 0.0 | 0.0 |  |
|  |  |  |  |  |
| **IgG N HKU1** (median) | 0.69 | 0.43 | 0.75 | 0.979 |
| (P25, P75) | (-1.60, 2.40) | (-1.55, 2.59) | (-1.49, 2.15) |  |
| ∆ ≥15% | 0.0 | 0.0 | 0.0 |  |
| ∇ ≥15% | 0.0 | 0.0 | 0.0 |  |
|  |  |  |  |  |
| **IgG NL 63** (median) | -0.35 | 0.10 | -0.33 | 0.923 |
| (P25, P75) | (-2.89, 3.44) | (-1.41, 2.35) | (-2.41, 3.06) |  |
| ∆ ≥15% | 0.0 | 0.0 | 0.0 |  |
| ∇ ≥15% | 0.0 | 0.0 | 0.0 |  |
|  |  |  |  |  |
| **IgG N OC43** (median) | -0.12 | 0.09 | 0.42 | 0.992 |
| (P25, P75) | (-3.75, 3.25) | (-2.03, 2.39) | (-4.40, 3.62) |  |
| ∆ ≥15% | 2.0 | 0.0 | 0.0 |  |
| ∇ ≥15% | 2.0 | 0.0 | 5.6 |  |
|  |  |  |  |  |

[ continued ]

**Supplemental Table 10b.** Continued

|  |  | | |  |
| --- | --- | --- | --- | --- |
| **Immunoglobulin^a^** | **< 46 years**  (N = 51) | **46 – 62 years**  (N = 49) | **≥ 63 years**  (N = 54) | **P^b^** |
| **IgM** |  |  |  |  |
| **IgM CMV pp150** (median) | 0.80 | 1.29 | 0.96 | 0.850 |
| (P25, P75) | (-2.61, 3.99) | (-2.96, 3.74) | (-0.89, 3.43) |  |
| ∆ ≥15% | 2.0 | 0.0 | 1.9 |  |
| ∇ ≥15% | 0.0 | 0.0 | 1.9 |  |
|  |  |  |  |  |
| **IgM CMV pp65** (median) | 0.19 | 0.95 | 0.59 | 0.620 |
| (P25, P75) | (-2.39, 2.94) | (-1.46, 3.48) | (-1.62, 4.39) |  |
| ∆ ≥15% | 0.0 | 0.0 | 1.9 |  |
| ∇ ≥15% | 0.0 | 0.0 | 0.0 |  |
|  |  |  |  |  |
| **IgM EBV EAD** (median) | 0.57 | 0.38 | 0.51 | 0.796 |
| (P25, P75) | (-2.45, 3.07) | (-2.16, 3.81) | (-2.47, 4.10) |  |
| ∆ ≥15% | 2.0 | 0.0 | 1.9 |  |
| ∇ ≥15% | 0.0 | 0.0 | 0.0 |  |
|  |  |  |  |  |
| **IgM VCAp18** (median) | 1.34 | 0.89 | 1.30 | 0.695 |
| (P25, P75) | (-3.38, 4.11) | (-2.32, 3.89) | (-1.70, 5.04) |  |
| ∆ ≥15% | 2.0 | 2.0 | 1.9 |  |
| ∇ ≥15% | 0.0 | 0.0 | 0.0 |  |
|  |  |  |  |  |
| **IgM N 229E** (median) | -0.14 | 0.14 | 1.81 | 0.105 |
| (P25, P75) | (-3.47, 4.20) | (-3.39, 3.29) | (-1.26, 6.55) |  |
| ∆ ≥15% | 3.9 | 0.0 | 3.7 |  |
| ∇ ≥15% | 0.0 | 0.0 | 1.9 |  |
|  |  |  |  |  |
| **IgM N HKU1** (median) | -0.11 | 0.61 | 1.78 | 0.047 |
| (P25, P75) | (-2.28, 3.65) | (-1.66, 3.30) | (-0.23, 4.41) |  |
| ∆ ≥15% | 2.0 | 2.0 | 1.9 |  |
| ∇ ≥15% | 0.0 | 0.0 | 0.0 |  |
|  |  |  |  |  |
| **IgM NL 63** (median) | 0.86 | 0.79 | 2.17 | 0.237 |
| (P25, P75) | (-2.61, 4.92) | (-3.81, 3.63) | (-2.20, 4.93) |  |
| ∆ ≥15% | 2.0 | 0.0 | 1.9 |  |
| ∇ ≥15% | 0.0 | 2.0 | 0.0 |  |
|  |  |  |  |  |
| **IgM N OC43** (median) | -0.99 | 0.26 | 1.52 | 0.013 |
| (P25, P75) | (-4.03, 2.30) | (-2.35, 6.55) | (-0.93, 4.59) |  |
| ∆ ≥15% | 0.0 | 4.1 | 1.9 |  |
| ∇ ≥15% | 0.0 | 0.0 | 0.0 |  |
|  |  |  |  |  |

[ continued ]

**Supplemental Table 10b.** Continued

|  |  | | |  |
| --- | --- | --- | --- | --- |
| **Immunoglobulin^a^** | **< 46 years**  (N = 51) | **46 – 62 years**  (N = 49) | **≥ 63 years**  (N = 54) | **P^b^** |
| **Total immunoglobulin** |  |  |  |  |
| **IgG1**  (median) | 0.46 | 1.06 | 1.48 | 0.072 |
| (P25, P75) | (-3.66, 2.30) | (-0.91, 3.45) | (-1.03, 4.36) |  |
| ∆ ≥15% | 0.0 | 0.0 | 0.0 |  |
| ∇ ≥15% | 0.0 | 0.0 | 0.0 |  |
|  |  |  |  |  |
| **IgG2** (median) | 0.51 | 0.80 | 0.73 | 0.496 |
| (P25, P75) | (-0.82, 1.79) | (-1.31, 2.50) | (-0.09, 2.52) |  |
| ∆ ≥15% | 0.0 | 0.0 | 0.0 |  |
| ∇ ≥15% | 0.0 | 0.0 | 0.0 |  |
|  |  |  |  |  |
| **IgG3** (median) | -0.73 | -1.18 | 0.54 | 0.004 |
| (P25, P75) | (-3.05, 0.80) | (-3.26, 0.38) | (-0.94, 2.39) |  |
| ∆ ≥15% | 0.0 | 0.0 | 0.0 |  |
| ∇ ≥15% | 0.0 | 0.0 | 0.0 |  |
|  |  |  |  |  |
| **IgG4** (median) | -0.02 | 2.22 | 2.38 | 0.052 |
| (P25, P75) | (-3.14, 3.26) | (-1.71, 5.47) | (-0.39, 4.48) |  |
| ∆ ≥15% | 2.0 | 2.0 | 0.0 |  |
| ∇ ≥15% | 0.0 | 2.0 | 0.0 |  |
|  |  |  |  |  |
| **Sum of IgGs** (median) | 0.31 | 0.59 | 1.24 | 0.034 |
| (P25, P75) | (-2.34, 1.98) | (-0.90, 2.81) | (0.05, 2.51) |  |
| ∆ ≥15% | 0.0 | 0.0 | 0.0 |  |
| ∇ ≥15% | 0.0 | 0.0 | 0.0 |  |
|  |  |  |  |  |
| **IgE** (median) | 1.90 | 0.57 | 4.36 | 0.198 |
| (P25, P75) | (-6.25, 8.94) | (-4.87, 11.12) | (-0.34, 13.39) |  |
| ∆ ≥15% | 15.7 | 14.3 | 20.4 |  |
| ∇ ≥15% | 13.7 | 10.2 | 3.7 |  |
|  |  |  |  |  |
| **IgA** (median) | 0.08 | 0.74 | 1.60 | 0.197 |
| (P25, P75) | (-2.58, 2.83) | (-2.52, 4.35) | (-0.25, 3.95) |  |
| ∆ ≥15% | 0.0 | 0.0 | 1.9 |  |
| ∇ ≥15% | 0.0 | 0.0 | 0.0 |  |
|  |  |  |  |  |
| **IgM** (median) | -0.43 | -0.15 | 1.15 | 0.100 |
| (P25, P75) | (-2.03, 1.92) | (-1.49, 3.01) | (-0.69, 4.03) |  |
| ∆ ≥15% | 0.0 | 0.0 | 0.0 |  |
| ∇ ≥15% | 0.0 | 0.0 | 0.0 |  |
|  |  |  |  |  |

P25: percentile 25th. P75: percentile 75th.

Age groups are based on each participants’ age in 2020-21.

^a^ Units used for computing the relative intraindividual change were base 10 logtransformed MFI for levels of isotype-antigen combinations, and base 10 logtransformed µg/mL for levels of total Igs.

^b^ Kruskal-Wallis test (two-tailed).

**Supplemental Table 11a.** Concentrations of 24 isotype-antigen combinations for cytomegalovirus, Epstein-Barr and common cold infections, and of total Igs in 2016-17 by body mass index (N=240).

|  |  |  |  |  |
| --- | --- | --- | --- | --- |
| **Immunoglobulin**  (MFI) | **Underweight or normal weight**  (N = 101) | **Overweight**  (N = 91) | **Obese**  (N = 48) | **P^a^** |
| **IgA** |  |  |  |  |
| **IgA CMV pp150** (median) | 490.0 | 521.0 | 486.5 | 0.780 |
| (P25, P75) | (325.0, 790.0) | (357.5, 874.5) | (334.5, 985.2) |  |
| Geometric mean | 593.0 | 611.2 | 591.7 |  |
|  |  |  |  |  |
| **IgA CMV pp65** (median) | 501.0 | 611.0 | 704.2 | 0.007 |
| (P25, P75) | (370.5, 814.8) | (430.5, 878.0) | (510.6, 1267) |  |
| Geometric mean | 613.1 | 726.2 | 804.4 |  |
|  |  |  |  |  |
| **IgA EBV EAD** (median) | 372.0 | 443.5 | 544.5 | 0.013 |
| (P25, P75) | (286.5, 567.8) | (328.0, 666.5) | (362.4, 877.6) |  |
| Geometric mean | 441.8 | 530.0 | 587.8 |  |
|  |  |  |  |  |
| **IgA VCAp18** (median) | 448.0 | 509.0 | 526.2 | 0.464 |
| (P25, P75) | (322.0, 716.0) | (337.5, 758.0) | (355.5, 806.1) |  |
| Geometric mean | 554.7 | 567.8 | 588.3 |  |
|  |  |  |  |  |
| **IgA N 229E** (median) | 1420 | 1224 | 1855 | 0.384 |
| (P25, P75) | (737.5, 3728) | (701.5, 3495) | (790.9, 4700) |  |
| Geometric mean | 1816 | 1618 | 2093 |  |
|  |  |  |  |  |
| **IgA N HKU1** (median) | 611.0 | 693.0 | 907.0 | 0.034 |
| (P25, P75) | (438.5, 1061) | (476.0, 1159) | (537.5, 1563) |  |
| Geometric mean | 727.6 | 843.5 | 979.5 |  |
|  |  |  |  |  |
| **IgA NL 63**  (median) | 1185 | 1952 | 2206 | 0.125 |
| (P25, P75) | (702.0, 3939) | (793.0, 5048) | (954.6, 7442) |  |
| Geometric mean | 1757 | 2125 | 2743 |  |
|  |  |  |  |  |
| **IgA N OC43** (median) | 1342 | 1732 | 1384 | 0.143 |
| (P25, P75) | (826.2, 2394) | (914.0, 3784) | (946.1, 2927) |  |
| Geometric mean | 1407 | 1843 | 1805 |  |
|  |  |  |  |  |

[ continued ]

**Supplemental Table 11a.** Continued.

|  |  |  |  |  |
| --- | --- | --- | --- | --- |
| **Immunoglobulin**  (MFI) | **Underweight or normal weight**  (N = 101) | **Overweight**  (N = 91) | **Obese**  (N = 48) | **P^a^** |
| **IgG** |  |  |  |  |
| **IgG CMV pp150** (median) | 13592 | 18463 | 16981 | 0.406 |
| (P25, P75) | (4770, 30230) | (5960, 31786) | (8276, 38419) |  |
| Geometric mean | 11939 | 14745 | 15298 |  |
|  |  |  |  |  |
| **IgG CMV pp65** (median) | 6850 | 6756 | 7013 | 0.936 |
| (P25, P75) | (4046, 11684) | (4492, 10900) | (4569, 10398) |  |
| Geometric mean | 6873 | 6871 | 6397 |  |
|  |  |  |  |  |
| **IgG EBV EAD** (median) | 7146 | 6602 | 7211 | 0.915 |
| (P25, P75) | (4450, 9854) | (4937, 9529) | (4749, 10385) |  |
| Geometric mean | 6785 | 6853 | 6962 |  |
|  |  |  |  |  |
| **IgG VCAp18** (median) | 8092 | 7400 | 7313 | 0.828 |
| (P25, P75) | (4150, 13639) | (4274, 15657) | (4612, 14398) |  |
| Geometric mean | 8145 | 8864 | 8733 |  |
|  |  |  |  |  |
| **IgG N 229E** (median) | 44878 | 45478 | 43887 | 0.986 |
| (P25, P75) | (24226, 81574) | (27383, 68320) | (25828, 77814) |  |
| Geometric mean | 40858 | 42476 | 42682 |  |
|  |  |  |  |  |
| **IgG N HKU1** (median) | 13882 | 14011 | 15078 | 0.910 |
| (P25, P75) | (9585, 19788) | (10910, 18268) | (9378, 19036) |  |
| Geometric mean | 13539 | 13876 | 13757 |  |
|  |  |  |  |  |
| **IgG NL 63**  (median) | 33353 | 39508 | 34875 | 0.810 |
| (P25, P75) | (20757, 58449) | (22601, 57348) | (20336, 60506) |  |
| Geometric mean | 33179 | 35673 | 33864 |  |
|  |  |  |  |  |
| **IgG N OC43** (median) | 19362 | 23511 | 21454 | 0.333 |
| (P25, P75) | (9866, 34673) | (11831, 40084) | (10896, 43680) |  |
| Geometric mean | 19254 | 22709 | 22108 |  |
|  |  |  |  |  |

[ continued ]

**Supplemental Table 11a.** Continued.

|  |  |  |  |  |
| --- | --- | --- | --- | --- |
| **Immunoglobulin**  (MFI) | **Underweight or normal weight**  (N = 101) | **Overweight**  (N = 91) | **Obese**  (N = 48) | **P^a^** |
| **IgM** |  |  |  |  |
| **IgM CMV pp150** (median) | 1476 | 1113 | 1277 | 0.003 |
| (P25, P75) | (962.2, 2991) | (676.0, 2192) | (724.4, 2420) |  |
| Geometric mean | 1666 | 1280 | 1378 |  |
|  |  |  |  |  |
| **IgM CMV pp65** (median) | 825.0 | 604.5 | 655.0 | <0.001 |
| (P25, P75) | (545.5, 1186) | (422.0, 920.0) | (427.6, 1036) |  |
| Geometric mean | 858.0 | 655.5 | 698.2 |  |
|  |  |  |  |  |
| **IgM EBV EAD** (median) | 456.0 | 427.0 | 423.8 | 0.013 |
| (P25, P75) | (352.5, 607.0) | (278.0, 623.0) | (304.2, 590.5) |  |
| Geometric mean | 482.8 | 426.9 | 435.8 |  |
|  |  |  |  |  |
| **IgM VCAp18** (median) | 1089 | 831.0 | 861.2 | <0.001 |
| (P25, P75) | (648.8, 1902) | (471.5, 1365) | (498.8, 1599) |  |
| Geometric mean | 1147 | 849.3 | 911.1 |  |
|  |  |  |  |  |
| **IgM N 229E** (median) | 1680 | 1305 | 1431 | <0.001 |
| (P25, P75) | (1176, 3103) | (800.0, 2040) | (863.6, 2378) |  |
| Geometric mean | 1769 | 1307 | 1444 |  |
|  |  |  |  |  |
| **IgM N HKU1** (median) | 1147 | 953.5 | 1022 | 0.003 |
| (P25, P75) | (827.5, 1805) | (627.0, 1524) | (705.6, 1652) |  |
| Geometric mean | 1245 | 993.4 | 1079 |  |
|  |  |  |  |  |
| **IgM NL 63**  (median) | 1132 | 779.0 | 948.2 | 0.001 |
| (P25, P75) | (758.8, 1799) | (513.0, 1328) | (591.5, 1568) |  |
| Geometric mean | 1234 | 891.7 | 1012 |  |
|  |  |  |  |  |
| **IgM N OC43** (median) | 2550 | 1860 | 2060 | 0.003 |
| (P25, P75) | (1319, 4096) | (1076, 3074) | (1121, 3496) |  |
| Geometric mean | 2526 | 1812 | 2065 |  |
|  |  |  |  |  |

[ continued ]

**Supplemental Table 11a.** Continued.

|  |  |  |  |  |
| --- | --- | --- | --- | --- |
| **Immunoglobulin**  (µg/mL) | **Underweight or normal weight**  (N = 101) | **Overweight**  (N = 91) | **Obese**  (N = 48) | **P^a^** |
| **Total immunoglobulin** |  |  |  |  |
| **IgG1** (median) | 6149 | 6080 | 6079 | 0.950 |
| (P25, P75) | (4727, 8308) | (4624, 8189) | (4910, 8088) |  |
| Geometric mean | 6292 | 6184 | 6285 |  |
|  |  |  |  |  |
| **IgG2** (median) | 3077 | 3059 | 3259 | 0.936 |
| (P25, P75) | (2439, 3640) | (2544, 3692) | (2509, 3731) |  |
| Geometric mean | 3037 | 3056 | 3015 |  |
|  |  |  |  |  |
| **IgG3** (median) | 1261 | 1198 | 1209 | 0.448 |
| (P25, P75) | (1051, 1479) | (964.8, 1480) | (1004, 1460) |  |
| Geometric mean | 1248 | 1135 | 1221 |  |
|  |  |  |  |  |
| **IgG4** (median) | 226.9 | 216.9 | 269.8 | 0.318 |
| (P25, P75) | (126.6, 336.7) | (113.0, 401.6) | (132.0, 544.8) |  |
| Geometric mean | 219.8 | 214.4 | 269.1 |  |
|  |  |  |  |  |
| **Sum of IgGs** (median) | 10929 | 10834 | 10963 | 0.902 |
| (P25, P75) | (8573, 13802) | (8851, 13721) | (9544, 13649) |  |
| Geometric mean | 11068 | 10933 | 11161 |  |
|  |  |  |  |  |
| **IgE** (median) | 0.23 | 0.21 | 0.25 | 0.759 |
| (P25, P75) | (0.15, 0.33) | (0.15, 0.35) | (0.15, 0.34) |  |
| Geometric mean | 0.23 | 0.23 | 0.24 |  |
|  |  |  |  |  |
| **IgA** (median) | 231.2 | 266.3 | 249.7 | 0.039 |
| (P25, P75) | (183.3, 282.0) | (200.5, 319.3) | (199.9, 288.7) |  |
| Geometric mean | 223.5 | 257.7 | 243.3 |  |
|  |  |  |  |  |
| **IgM** (median) | 1139 | 885.8 | 787.0 | <0.001 |
| (P25, P75) | (737.2, 1473) | (700.7, 1194) | (578.3, 1042) |  |
| Geometric mean | 1072 | 909.7 | 786.3 |  |
|  |  |  |  |  |

P25: percentile 25th. P75: percentile 75th.

<LOQ: value lower than the minimum concentration of the corresponding range of limits of quantification.

See Supplemental Table 1.

^a^ Kruskal-Wallis test (two-tailed).

**Supplemental Table 11b.** Relative intraindividual change (%) from 2016-17 to 2020-21 of concentrations of 24 isotype-antigen combinations for cytomegalovirus, Epstein-Barr and common cold infections, and of total Igs, by body mass index (N=154).

|  |  | | |  |
| --- | --- | --- | --- | --- |
| **Immunoglobulin^a^** | **Normal weight** (N = 68) | **Overweight**  (N = 49) | **Obese**  (N = 36) | **P^b^** |
| **IgA** |  |  |  |  |
| **IgA CMV pp150** (median) | 0.69 | 0.37 | 0.25 | 0.793 |
| (P25, P75) | (-2.30, 3.75) | (-3.74, 3.95) | (-2.03, 2.96) |  |
| ∆ ≥15% | 5.9 | 4.1 | 2.8 |  |
| ∇ ≥15% | 0.0 | 2.0 | 0.0 |  |
|  |  |  |  |  |
| **IgA CMV pp65** (median) | 1.06 | 0.52 | 1.87 | 0.245 |
| (P25, P75) | (-2.45, 2.58) | (-2.35, 4.79) | (-1.99, 6.63) |  |
| ∆ ≥15% | 1.5 | 2.0 | 2.8 |  |
| ∇ ≥15% | 1.5 | 0.0 | 0.0 |  |
|  |  |  |  |  |
| **IgA EBV EAD** (median) | 0.57 | 0.09 | 0.30 | 0.893 |
| (P25, P75) | (-1.91, 3.46) | (-2.67, 4.60) | (-2.61, 2.89) |  |
| ∆ ≥15% | 1.5 | 2.0 | 0.0 |  |
| ∇ ≥15% | 1.5 | 2.0 | 0.0 |  |
|  |  |  |  |  |
| **IgA VCAp18** (median) | 0.95 | 0.45 | 0.59 | 0.977 |
| (P25, P75) | (-2.06, 3.37) | (-2.91, 5.20) | (-2.00, 2.95) |  |
| ∆ ≥15% | 1.5 | 2.0 | 0.0 |  |
| ∇ ≥15% | 1.5 | 0.0 | 0.0 |  |
|  |  |  |  |  |
| **IgA N 229E** (median) | -0.71 | -2.14 | 1.61 | 0.336 |
| (P25, P75) | (-4.29, 3.21) | (-4.67, 4.57) | (-1.58, 3.23) |  |
| ∆ ≥15% | 2.9 | 4.1 | 0.0 |  |
| ∇ ≥15% | 4.4 | 10.2 | 0.0 |  |
|  |  |  |  |  |
| **IgA N HKU1** (median) | 0.70 | 0.80 | 1.19 | 0.659 |
| (P25, P75) | (-2.53, 2.61) | (-2.55, 3.18) | (-2.38, 3.60) |  |
| ∆ ≥15% | 2.9 | 4.1 | 0.0 |  |
| ∇ ≥15% | 0.0 | 0.0 | 0.0 |  |
|  |  |  |  |  |
| **IgA NL 63** (median) | -0.19 | -0.76 | -0.47 | 0.932 |
| (P25, P75) | (-3.18, 4.57) | (-6.03, 7.74) | (-3.63, 3.55) |  |
| ∆ ≥15% | 4.4 | 10.2 | 5.6 |  |
| ∇ ≥15% | 4.4 | 6.1 | 2.8 |  |
|  |  |  |  |  |
| **IgA N OC43** (median) | 0.45 | 0.31 | -0.31 | 0.698 |
| (P25, P75) | (-2.19, 3.46) | (-3.67, 3.03) | (-2.51, 2.46) |  |
| ∆ ≥15% | 4.4 | 2.0 | 0.0 |  |
| ∇ ≥15% | 0.0 | 0.0 | 2.8 |  |
|  |  |  |  |  |

[ continued ]

**Supplemental Table 11b.** Continued

|  |  | | |  |
| --- | --- | --- | --- | --- |
| **Immunoglobulin^a^** | **Normal weight** (N = 68) | **Overweight**  (N = 49) | **Obese**  (N = 36) | **P^b^** |
| **IgG** |  |  |  |  |
| **IgG CMV pp150** (median) | 2.30 | 0.92 | 0.79 | 0.047 |
| (P25, P75) | (-0.34, 5.26) | (-2.79, 2.48) | (-1.30, 3.80) |  |
| ∆ ≥15% | 2.9 | 2.0 | 0.0 |  |
| ∇ ≥15% | 0.0 | 0.0 | 0.0 |  |
|  |  |  |  |  |
| **IgG CMV pp65** (median) | 1.45 | 0.92 | 0.31 | 0.769 |
| (P25, P75) | (-2.18, 4.12) | (-1.89, 2.50) | (-2.98, 2.76) |  |
| ∆ ≥15% | 0.0 | 0.0 | 2.8 |  |
| ∇ ≥15% | 0.0 | 0.0 | 0.0 |  |
|  |  |  |  |  |
| **IgG EBV EAD** (median) | 1.54 | -0.51 | -0.21 | 0.075 |
| (P25, P75) | (-2.14, 3.94) | (-2.17, 1.91) | (-3.07, 1.29) |  |
| ∆ ≥15% | 0.0 | 0.0 | 0.0 |  |
| ∇ ≥15% | 0.0 | 0.0 | 0.0 |  |
|  |  |  |  |  |
| **IgG VCAp18** (median) | 1.09 | 0.23 | 0.13 | 0.363 |
| (P25, P75) | (-1.31, 4.57) | (-1.32, 2.68) | (-2.66, 2.41) |  |
| ∆ ≥15% | 2.9 | 0.0 | 2.8 |  |
| ∇ ≥15% | 1.5 | 0.0 | 0.0 |  |
|  |  |  |  |  |
| **IgG N 229E** (median) | 0.90 | -0.38 | -0.46 | 0.329 |
| (P25, P75) | (-2.30, 4.34) | (-2.52, 1.99) | (-3.00, 2.23) |  |
| ∆ ≥15% | 1.5 | 0.0 | 0.0 |  |
| ∇ ≥15% | 0.0 | 0.0 | 0.0 |  |
|  |  |  |  |  |
| **IgG N HKU1** (median) | 1.10 | 0.74 | 0.15 | 0.294 |
| (P25, P75) | (-1.45, 2.94) | (-1.74, 1.97) | (-2.14, 1.80) |  |
| ∆ ≥15% | 0.0 | 0.0 | 0.0 |  |
| ∇ ≥15% | 0.0 | 0.0 | 0.0 |  |
|  |  |  |  |  |
| **IgG NL 63** (median) | 0.26 | -0.21 | -0.50 | 0.740 |
| (P25, P75) | (-2.67, 2.99) | (-2.94, 2.46) | (-2.11, 2.05) |  |
| ∆ ≥15% | 0.0 | 0.0 | 0.0 |  |
| ∇ ≥15% | 0.0 | 0.0 | 0.0 |  |
|  |  |  |  |  |
| **IgG N OC43** (median) | 0.87 | 0.09 | -0.84 | 0.176 |
| (P25, P75) | (-1.91, 3.48) | (-3.85, 2.71) | (-4.17, 2.04) |  |
| ∆ ≥15% | 1.5 | 0.0 | 0.0 |  |
| ∇ ≥15% | 1.5 | 4.1 | 2.8 |  |
|  |  |  |  |  |

[ continued ]

**Supplemental Table 11b.** Continued

|  |  | | |  |
| --- | --- | --- | --- | --- |
| **Immunoglobulin^a^** | **Normal weight** (N = 68) | **Overweight**  (N = 49) | **Obese**  (N = 36) | **P^b^** |
| **IgM** |  |  |  |  |
| **IgM CMV pp150** (median) | 1.56 | 1.43 | -0.79 | 0.140 |
| (P25, P75) | (-2.05, 5.73) | (-0.78, 3.85) | (-2.86, 1.71) |  |
| ∆ ≥15% | 0.0 | 2.0 | 2.8 |  |
| ∇ ≥15% | 0.0 | 2.0 | 0.0 |  |
|  |  |  |  |  |
| **IgM CMV pp65** (median) | 0.93 | -0.10 | 0.39 | 0.491 |
| (P25, P75) | (-1.37, 3.70) | (-2.77, 2.24) | (-2.20, 4.35) |  |
| ∆ ≥15% | 0.0 | 2.0 | 0.0 |  |
| ∇ ≥15% | 0.0 | 0.0 | 0.0 |  |
|  |  |  |  |  |
| **IgM EBV EAD** (median) | 1.31 | -0.22 | 0.05 | 0.539 |
| (P25, P75) | (-1.88, 3.69) | (-2.86, 3.15) | (-2.80, 3.65) |  |
| ∆ ≥15% | 0.0 | 4.1 | 0.0 |  |
| ∇ ≥15% | 0.0 | 0.0 | 0.0 |  |
|  |  |  |  |  |
| **IgM VCAp18** (median) | 1.54 | 0.33 | 0.42 | 0.532 |
| (P25, P75) | (-2.08, 4.10) | (-2.53, 4.24) | (-2.34, 3.71) |  |
| ∆ ≥15% | 4.4 | 0.0 | 0.0 |  |
| ∇ ≥15% | 0.0 | 0.0 | 0.0 |  |
|  |  |  |  |  |
| **IgM N 229E** (median) | 0.55 | 0.81 | 1.60 | 0.628 |
| (P25, P75) | (-3.24, 4.55) | (-3.35, 4.06) | (-2.15, 5.99) |  |
| ∆ ≥15% | 1.5 | 4.1 | 2.8 |  |
| ∇ ≥15% | 0.0 | 0.0 | 2.8 |  |
|  |  |  |  |  |
| **IgM N HKU1** (median) | 0.53 | 1.19 | 1.16 | 0.870 |
| (P25, P75) | (-1.55, 3.48) | (-1.46, 2.75) | (-1.56, 4.85) |  |
| ∆ ≥15% | 1.5 | 0.0 | 2.8 |  |
| ∇ ≥15% | 0.0 | 0.0 | 0.0 |  |
|  |  |  |  |  |
| **IgM NL 63** (median) | 0.73 | 0.66 | 3.34 | 0.130 |
| (P25, P75) | (-3.85, 3.70) | (-2.89, 4.57) | (-0.71, 5.33) |  |
| ∆ ≥15% | 0.0 | 4.1 | 0.0 |  |
| ∇ ≥15% | 1.5 | 0.0 | 0.0 |  |
|  |  |  |  |  |
| **IgM N OC43** (median) | 0.12 | 0.70 | 0.92 | 0.765 |
| (P25, P75) | (-3.28, 4.69) | (-2.92, 3.34) | (-1.75, 4.51) |  |
| ∆ ≥15% | 2.9 | 2.0 | 0.0 |  |
| ∇ ≥15% | 0.0 | 0.0 | 0.0 |  |
|  |  |  |  |  |

[ continued ]

**Supplemental Table 11b.** Continued

|  |  | | |  |
| --- | --- | --- | --- | --- |
| **Immunoglobulin^a^** | **Normal weight** (N = 68) | **Overweight**  (N = 49) | **Obese**  (N = 36) | **P^b^** |
| **Total immunoglobulin** |  |  |  |  |
| **IgG1**  (median) | 0.84 | 1.89 | 0.54 | 0.514 |
| (P25, P75) | (-1.92, 2.98) | (-1.18, 4.20) | (-1.30, 2.78) |  |
| ∆ ≥15% | 0.0 | 0.0 | 0.0 |  |
| ∇ ≥15% | 0.0 | 0.0 | 0.0 |  |
|  |  |  |  |  |
| **IgG2** (median) | 0.66 | 0.81 | 0.60 | 0.920 |
| (P25, P75) | (-0.61, 1.84) | (-0.69, 2.58) | (-0.91, 2.49) |  |
| ∆ ≥15% | 0.0 | 0.0 | 0.0 |  |
| ∇ ≥15% | 0.0 | 0.0 | 0.0 |  |
|  |  |  |  |  |
| **IgG3** (median) | -0.87 | -0.01 | 0.09 | 0.140 |
| (P25, P75) | (-3.21, 0.71) | (-1.62, 2.14) | (-2.66, 1.99) |  |
| ∆ ≥15% | 0.0 | 0.0 | 0.0 |  |
| ∇ ≥15% | 0.0 | 0.0 | 0.0 |  |
|  |  |  |  |  |
| **IgG4** (median) | 0.71 | 1.21 | 1.97 | 0.979 |
| (P25, P75) | (-1.82, 4.55) | (-1.55, 4.49) | (-2.14, 4.08) |  |
| ∆ ≥15% | 0.0 | 2 | 0.0 |  |
| ∇ ≥15% | 1.5 | 0.0 | 0.0 |  |
|  |  |  |  |  |
| **Sum of IgGs** (median) | 0.43 | 1.21 | 0.51 | 0.452 |
| (P25, P75) | (-1.10, 2.02) | (-0.74, 2.77) | (-1.05, 2.31) |  |
| ∆ ≥15% | 0.0 | 0.0 | 0.0 |  |
| ∇ ≥15% | 0.0 | 0.0 | 0.0 |  |
|  |  |  |  |  |
| **IgE** (median) | 0.75 | 3.15 | 3.61 | 0.287 |
| (P25, P75) | (-11.14, 10.24) | (-2.16, 10.67) | (-0.50, 13.53) |  |
| ∆ ≥15% | 14.7 | 16.3 | 22.2 |  |
| ∇ ≥15% | 13.2 | 6.1 | 5.6 |  |
|  |  |  |  |  |
| **IgA** (median) | 0.77 | 0.74 | 1.75 | 0.420 |
| (P25, P75) | (-2.31, 3.91) | (-2.18, 2.24) | (-1.67, 4.55) |  |
| ∆ ≥15% | 0.0 | 2 | 0.0 |  |
| ∇ ≥15% | 0.0 | 0.0 | 0.0 |  |
|  |  |  |  |  |
| **IgM** (median) | -0.03 | 0.50 | 0.90 | 0.960 |
| (P25, P75) | (-1.48, 3.94) | (-1.52, 2.38) | (-2.05, 3.34) |  |
| ∆ ≥15% | 0.0 | 0.0 | 0.0 |  |
| ∇ ≥15% | 0.0 | 0.0 | 0.0 |  |
|  |  |  |  |  |

P25: percentile 25th. P75: percentile 75th.

Body mass index (BMI) is based on each participant's BMI in 2020-21.

^a^ Units used for computing the relative intraindividual change were base 10 logtransformed MFI for levels of isotype-antigen combinations, and base 10 logtransformed µg/mL for levels of total Igs.

^b^ Kruskal-Wallis test (two-tailed).

**Supplemental Table 12a.** Concentrations of 24 isotype-antigen combinations for cytomegalovirus, Epstein-Barr and common cold infections, and of total Igs in 2016-17 by tobacco smoking (N=240).

|  |  |  |  |  |
| --- | --- | --- | --- | --- |
| **Immunoglobulin**  (MFI) | **Non-smoker**  (N = 93) | **Former smoker**  (N = 99) | **Current smoker**  (N = 48) | **P^a^** |
| **IgA** |  |  |  |  |
| **IgA CMV pp150** (median) | 507.0 | 498.5 | 463.2 | 0.657 |
| (P25, P75) | (331.8, 838.5) | (358.0, 828.5) | (318.2, 822.2) |  |
| Geometric mean | 574.2 | 614.7 | 619.3 |  |
|  |  |  |  |  |
| **IgA CMV pp65** (median) | 563.0 | 685.0 | 497.5 | 0.001 |
| (P25, P75) | (402.2, 847.5) | (438.0, 1440) | (366.2, 681.6) |  |
| Geometric mean | 624.1 | 865.1 | 526.6 |  |
|  |  |  |  |  |
| **IgA EBV EAD** (median) | 388.0 | 477.5 | 390.0 | 0.082 |
| (P25, P75) | (293.8, 585.2) | (345.0, 741.5) | (311.9, 621.0) |  |
| Geometric mean | 463.6 | 564.9 | 455.6 |  |
|  |  |  |  |  |
| **IgA VCAp18** (median) | 418.0 | 521.5 | 503.2 | 0.086 |
| (P25, P75) | (311.0, 749.0) | (359.0, 834.5) | (340.4, 653.1) |  |
| Geometric mean | 527.9 | 633.1 | 515.3 |  |
|  |  |  |  |  |
| **IgA N 229E** (median) | 1069 | 1838 | 1320 | 0.084 |
| (P25, P75) | (645.5, 3705) | (830.5, 3753) | (739.0, 3547) |  |
| Geometric mean | 1581 | 2141 | 1567 |  |
|  |  |  |  |  |
| **IgA N HKU1** (median) | 597.0 | 767.0 | 630.2 | 0.033 |
| (P25, P75) | (431.0, 1163) | (534.0, 1184) | (436.5, 1092) |  |
| Geometric mean | 706.6 | 916.3 | 852.7 |  |
|  |  |  |  |  |
| **IgA NL 63**  (median) | 1538 | 1461 | 1837 | 0.854 |
| (P25, P75) | (735.2, 4995) | (809.0, 4285) | (765.5, 6754) |  |
| Geometric mean | 2015 | 2049 | 2195 |  |
|  |  |  |  |  |
| **IgA N OC43** (median) | 1290 | 1647 | 1440 | 0.199 |
| (P25, P75) | (824.8, 2558) | (944.5, 2950) | (832.5, 3304) |  |
| Geometric mean | 1410 | 1899 | 1616 |  |
|  |  |  |  |  |

[ continued ]

**Supplemental Table 12a.** Continued.

|  |  |  |  |  |
| --- | --- | --- | --- | --- |
| **Immunoglobulin**  (MFI) | **Non-smoker**  (N = 93) | **Former smoker**  (N = 99) | **Current smoker**  (N = 48) | **P^a^** |
| **IgG** |  |  |  |  |
| **IgG CMV pp150** (median) | 15073 | 14680 | 17841 | 0.731 |
| (P25, P75) | (5167, 28634) | (5303, 36396) | (5456, 37335) |  |
| Geometric mean | 12628 | 14086 | 14558 |  |
|  |  |  |  |  |
| **IgG CMV pp65** (median) | 7057 | 6697 | 6767 | 0.334 |
| (P25, P75) | (4968, 11166) | (3661, 11865) | (4133, 8664) |  |
| Geometric mean | 7339 | 6473 | 6372 |  |
|  |  |  |  |  |
| **IgG EBV EAD** (median) | 7177 | 6720 | 7540 | 0.542 |
| (P25, P75) | (4890, 10081) | (4748, 9164) | (4657, 11198) |  |
| Geometric mean | 7019 | 6524 | 7203 |  |
|  |  |  |  |  |
| **IgG VCAp18** (median) | 7815 | 7400 | 8888 | 0.477 |
| (P25, P75) | (4230, 13933) | (4146, 13412) | (4856, 15721) |  |
| Geometric mean | 8175 | 8355 | 9658 |  |
|  |  |  |  |  |
| **IgG N 229E** (median) | 42135 | 43472 | 48624 | 0.410 |
| (P25, P75) | (22762, 77039) | (26623, 68493) | (29682, 80750) |  |
| Geometric mean | 40586 | 40555 | 47261 |  |
|  |  |  |  |  |
| **IgG N HKU1** (median) | 13536 | 14752 | 13974 | 0.459 |
| (P25, P75) | (9878, 17230) | (10606, 19962) | (8573, 21359) |  |
| Geometric mean | 13313 | 14037 | 13824 |  |
|  |  |  |  |  |
| **IgG NL 63**  (median) | 39623 | 33123 | 42683 | 0.111 |
| (P25, P75) | (20941, 60736) | (19039, 49903) | (23546, 65837) |  |
| Geometric mean | 35345 | 30877 | 39864 |  |
|  |  |  |  |  |
| **IgG N OC43** (median) | 21022 | 19339 | 26634 | 0.081 |
| (P25, P75) | (11287, 36880) | (10624, 35728) | (16411, 46987) |  |
| Geometric mean | 20059 | 19680 | 26692 |  |
|  |  |  |  |  |

[ continued ]

**Supplemental Table 12a.** Continued.

|  |  |  |  |  |
| --- | --- | --- | --- | --- |
| **Immunoglobulin**  (MFI) | **Non-smoker**  (N = 93) | **Former smoker**  (N = 99) | **Current smoker**  (N = 48) | **P^a^** |
| **IgM** |  |  |  |  |
| **IgM CMV pp150** (median) | 1310 | 1115 | 1328 | 0.218 |
| (P25, P75) | (877.0, 2702) | (616.0, 2020) | (741.5, 2225) |  |
| Geometric mean | 1561 | 1235 | 1358 |  |
|  |  |  |  |  |
| **IgM CMV pp65** (median) | 726.0 | 584.0 | 654.5 | 0.051 |
| (P25, P75) | (454.2, 1175) | (401.0, 852.0) | (418.4, 1076) |  |
| Geometric mean | 800.3 | 611.8 | 703.6 |  |
|  |  |  |  |  |
| **IgM EBV EAD** (median) | 449.5 | 387.0 | 484.2 | 0.007 |
| (P25, P75) | (323.8, 730.2) | (276.5, 513.0) | (328.6, 675.0) |  |
| Geometric mean | 476.2 | 383.4 | 477.9 |  |
|  |  |  |  |  |
| **IgM VCAp18** (median) | 899.0 | 685.5 | 1030 | 0.041 |
| (P25, P75) | (525.8, 1848) | (466.0, 1348) | (547.0, 1578) |  |
| Geometric mean | 996.5 | 802.7 | 994.6 |  |
|  |  |  |  |  |
| **IgM N 229E** (median) | 1469 | 1305 | 1681 | 0.107 |
| (P25, P75) | (942.8, 2448) | (752.0, 2264) | (1129, 2688) |  |
| Geometric mean | 1548 | 1292 | 1588 |  |
|  |  |  |  |  |
| **IgM N HKU1** (median) | 1076 | 975.0 | 1027 | 0.070 |
| (P25, P75) | (771.5, 1746) | (627.0, 1522) | (754.6, 1705) |  |
| Geometric mean | 1159 | 952.4 | 1214 |  |
|  |  |  |  |  |
| **IgM NL 63**  (median) | 1118 | 769.0 | 1032 | 0.005 |
| (P25, P75) | (696.5, 1763) | (496.0, 1321) | (675.4, 1866) |  |
| Geometric mean | 1161 | 842.2 | 1130 |  |
|  |  |  |  |  |
| **IgM N OC43** (median) | 2122 | 1606 | 2390 | 0.089 |
| (P25, P75) | (1159, 3525) | (1013, 3370) | (1279, 4194) |  |
| Geometric mean | 2134 | 1848 | 2433 |  |
|  |  |  |  |  |

[ continued ]

**Supplemental Table 12a.** Continued.

|  |  |  |  |  |
| --- | --- | --- | --- | --- |
| **Immunoglobulin**  (µg/mL) | **Non-smoker**  (N = 93) | **Former smoker**  (N = 99) | **Current smoker**  (N = 48) | **P^a^** |
| **Total immunoglobulin** |  |  |  |  |
| **IgG1** (median) | 6120 | 6166 | 5870 | 0.724 |
| (P25, P75) | (4649, 8451) | (4793, 8376) | (4630, 7742) |  |
| Geometric mean | 6429 | 6176 | 6063 |  |
|  |  |  |  |  |
| **IgG2** (median) | 3077 | 3212 | 2738 | 0.060 |
| (P25, P75) | (2479, 3701) | (2485, 3800) | (2465, 3399) |  |
| Geometric mean | 3110 | 3092 | 2808 |  |
|  |  |  |  |  |
| **IgG3** (median) | 1203 | 1284 | 1187 | 0.858 |
| (P25, P75) | (989.4, 1501) | (990.8, 1473) | (1063, 1415) |  |
| Geometric mean | 1225 | 1185 | 1176 |  |
|  |  |  |  |  |
| **IgG4** (median) | 238.3 | 221.1 | 224.7 | 0.878 |
| (P25, P75) | (130.7, 388.1) | (111.0, 464.6) | (127.5, 346.9) |  |
| Geometric mean | 233.3 | 227.1 | 213.8 |  |
|  |  |  |  |  |
| **Sum of IgGs** (median) | 11256 | 10903 | 10450 | 0.240 |
| (P25, P75) | (8766, 13993) | (9403, 13721) | (8393, 12520) |  |
| Geometric mean | 11368 | 10996 | 10494 |  |
|  |  |  |  |  |
| **IgE** (median) | 0.22 | 0.24 | 0.21 | 0.878 |
| (P25, P75) | (0.16, 0.34) | (0.14, 0.34) | (0.14, 0.33) |  |
| Geometric mean | 0.23 | 0.23 | 0.23 |  |
|  |  |  |  |  |
| **IgA** (median) | 243.9 | 250.1 | 239.0 | 0.360 |
| (P25, P75) | (188.7, 291.3) | (200.5, 304.2) | (162.3, 320.8) |  |
| Geometric mean | 233.7 | 251.5 | 229.1 |  |
|  |  |  |  |  |
| **IgM** (median) | 1124 | 846.1 | 1024 | 0.025 |
| (P25, P75) | (747.6, 1459) | (686.0, 1052) | (582.4, 1347) |  |
| Geometric mean | 1028 | 880.1 | 937.3 |  |
|  |  |  |  |  |

P25: percentile 25th. P75: percentile 75th.

<LOQ: value lower than the minimum concentration of the corresponding range of limits of quantification.

See Supplemental Table 1.

^a^ Kruskal-Wallis test (two-tailed).

**Supplemental Table 12b.** Relative intraindividual change (%) from 2016-17 to 2020-21 of concentrations of 24 isotype-antigen combinations for cytomegalovirus, Epstein-Barr and common cold infections, and of total Igs, by tobacco smoking (N=154).

|  |  | | |  |
| --- | --- | --- | --- | --- |
| **Immunoglobulin^a^** | **Non-smoker**  (N = 59) | **Former smoker**  (N = 56) | **Current smoker**  (N = 39) | **P^b^** |
| **IgA** |  |  |  |  |
| **IgA CMV pp150** (median) | -0.02 | -0.16 | 1.61 | 0.173 |
| (P25, P75) | (-2.75, 3.12) | (-2.21, 3.24) | (-0.91, 4.61) |  |
| ∆ ≥15% | 1.7 | 3.6 | 10.3 |  |
| ∇ ≥15% | 1.7 | 0.0 | 0.0 |  |
|  |  |  |  |  |
| **IgA CMV pp65** (median) | 0.74 | 0.32 | 1.74 | 0.386 |
| (P25, P75) | (-2.50, 2.76) | (-2.37, 4.56) | (-1.17, 4.80) |  |
| ∆ ≥15% | 0.0 | 1.8 | 5.1 |  |
| ∇ ≥15% | 0.0 | 0.0 | 2.6 |  |
|  |  |  |  |  |
| **IgA EBV EAD** (median) | 0.09 | -0.40 | 0.75 | 0.680 |
| (P25, P75) | (-1.83, 3.04) | (-2.38, 3.87) | (-1.79, 4.07) |  |
| ∆ ≥15% | 0.0 | 0.0 | 5.1 |  |
| ∇ ≥15% | 0.0 | 0.0 | 5.1 |  |
|  |  |  |  |  |
| **IgA VCAp18** (median) | 0.45 | 0.82 | 1.12 | 0.289 |
| (P25, P75) | (-2.73, 2.25) | (-2.38, 4.20) | (-0.49, 3.85) |  |
| ∆ ≥15% | 1.7 | 0.0 | 5.1 |  |
| ∇ ≥15% | 0.0 | 0.0 | 2.6 |  |
|  |  |  |  |  |
| **IgA N 229E** (median) | 0.58 | -1.35 | 1.37 | 0.332 |
| (P25, P75) | (-3.22, 5.33) | (-3.87, 2.20) | (-4.15, 3.44) |  |
| ∆ ≥15% | 1.7 | 0.0 | 7.7 |  |
| ∇ ≥15% | 6.8 | 3.6 | 5.1 |  |
|  |  |  |  |  |
| **IgA N HKU1** (median) | 0.90 | -0.55 | 1.86 | 0.134 |
| (P25, P75) | (-2.49, 2.86) | (-2.78, 2.91) | (-0.43, 3.59) |  |
| ∆ ≥15% | 0.0 | 3.6 | 5.1 |  |
| ∇ ≥15% | 0.0 | 0.0 | 0.0 |  |
|  |  |  |  |  |
| **IgA NL 63** (median) | -0.76 | -0.65 | 0.27 | 0.454 |
| (P25, P75) | (-5.25, 7.02) | (-3.63, 2.78) | (-1.98, 6.09) |  |
| ∆ ≥15% | 6.8 | 5.4 | 7.7 |  |
| ∇ ≥15% | 6.8 | 0.0 | 7.7 |  |
|  |  |  |  |  |
| **IgA N OC43** (median) | 0.48 | 0.18 | 0.78 | 0.819 |
| (P25, P75) | (-2.96, 3.83) | (-2.69, 2.48) | (-2.86, 3.62) |  |
| ∆ ≥15% | 1.7 | 1.8 | 5.1 |  |
| ∇ ≥15% | 1.7 | 0.0 | 0.0 |  |
|  |  |  |  |  |

[ continued ]

**Supplemental Table 12b.** Continued

|  |  | | |  |
| --- | --- | --- | --- | --- |
| **Immunoglobulin^a^** | **Non-smoker**  (N = 59) | **Former smoker**  (N = 56) | **Current smoker**  (N = 39) | **P^b^** |
| **IgG** |  |  |  |  |
| **IgG CMV pp150** (median) | 1.33 | 1.25 | 1.76 | 0.571 |
| (P25, P75) | (-1.70, 3.99) | (-0.35, 3.80) | (-2.45, 4.55) |  |
| ∆ ≥15% | 0.0 | 1.8 | 5.1 |  |
| ∇ ≥15% | 0.0 | 0.0 | 0.0 |  |
|  |  |  |  |  |
| **IgG CMV pp65** (median) | 0.67 | 1.00 | 1.68 | 0.682 |
| (P25, P75) | (-1.88, 2.51) | (-1.74, 2.59) | (-4.12, 5.69) |  |
| ∆ ≥15% | 1.7 | 0.0 | 0.0 |  |
| ∇ ≥15% | 0.0 | 0.0 | 0.0 |  |
|  |  |  |  |  |
| **IgG EBV EAD** (median) | 0.37 | 0.71 | 0.51 | 0.840 |
| (P25, P75) | (-2.09, 2.61) | (-2.25, 2.34) | (-2.82, 3.95) |  |
| ∆ ≥15% | 0.0 | 0.0 | 0.0 |  |
| ∇ ≥15% | 0.0 | 0.0 | 0.0 |  |
|  |  |  |  |  |
| **IgG VCAp18** (median) | 0.23 | 0.73 | 1.05 | 0.652 |
| (P25, P75) | (-1.47, 3.13) | (-1.88, 2.23) | (-2.28, 4.82) |  |
| ∆ ≥15% | 5.1 | 0.0 | 0.0 |  |
| ∇ ≥15% | 1.7 | 0.0 | 0.0 |  |
|  |  |  |  |  |
| **IgG N 229E** (median) | 0.30 | -0.50 | 0.48 | 0.702 |
| (P25, P75) | (-2.50, 2.88) | (-2.56, 2.39) | (-2.75, 3.35) |  |
| ∆ ≥15% | 0.0 | 0.0 | 2.6 |  |
| ∇ ≥15% | 0.0 | 0.0 | 0.0 |  |
|  |  |  |  |  |
| **IgG N HKU1** (median) | 0.48 | 0.44 | 1.11 | 0.850 |
| (P25, P75) | (-1.60, 2.40) | (-1.48, 2.10) | (-2.34, 2.95) |  |
| ∆ ≥15% | 0.0 | 0.0 | 0.0 |  |
| ∇ ≥15% | 0.0 | 0.0 | 0.0 |  |
|  |  |  |  |  |
| **IgG NL 63** (median) | -0.28 | 0.12 | 0.40 | 0.620 |
| (P25, P75) | (-2.92, 2.48) | (-2.24, 2.32) | (-2.89, 3.64) |  |
| ∆ ≥15% | 0.0 | 0.0 | 0.0 |  |
| ∇ ≥15% | 0.0 | 0.0 | 0.0 |  |
|  |  |  |  |  |
| **IgG N OC43** (median) | -0.23 | 0.46 | -0.13 | 0.837 |
| (P25, P75) | (-2.97, 3.38) | (-3.41, 2.73) | (-3.75, 2.68) |  |
| ∆ ≥15% | 0.0 | 0.0 | 2.6 |  |
| ∇ ≥15% | 5.1 | 0.0 | 2.6 |  |
|  |  |  |  |  |

[ continued ]

**Supplemental Table 12b.** Continued

|  |  | | |  |
| --- | --- | --- | --- | --- |
| **Immunoglobulin^a^** | **Non-smoker**  (N = 59) | **Former smoker**  (N = 56) | **Current smoker**  (N = 39) | **P^b^** |
| **IgM** |  |  |  |  |
| **IgM CMV pp150** (median) | -0.01 | 1.08 | 2.32 | 0.039 |
| (P25, P75) | (-2.25, 2.80) | (-2.25, 3.45) | (-1.59, 6.59) |  |
| ∆ ≥15% | 0.0 | 1.8 | 2.6 |  |
| ∇ ≥15% | 1.7 | 0.0 | 0.0 |  |
|  |  |  |  |  |
| **IgM CMV pp65** (median) | 0.66 | 0.10 | 0.57 | 0.768 |
| (P25, P75) | (-1.64, 4.24) | (-1.93, 2.16) | (-1.95, 4.09) |  |
| ∆ ≥15% | 1.7 | 0.0 | 0.0 |  |
| ∇ ≥15% | 0.0 | 0.0 | 0.0 |  |
|  |  |  |  |  |
| **IgM EBV EAD** (median) | 1.34 | 0.38 | 0.35 | 0.979 |
| (P25, P75) | (-2.92, 3.85) | (-2.18, 3.57) | (-1.76, 3.58) |  |
| ∆ ≥15% | 1.7 | 1.8 | 0.0 |  |
| ∇ ≥15% | 0.0 | 0.0 | 0.0 |  |
|  |  |  |  |  |
| **IgM VCAp18** (median) | 0.64 | 1.35 | 1.23 | 0.886 |
| (P25, P75) | (-2.77, 4.96) | (-2.08, 2.55) | (-2.33, 5.42) |  |
| ∆ ≥15% | 1.7 | 1.8 | 2.6 |  |
| ∇ ≥15% | 0.0 | 0.0 | 0.0 |  |
|  |  |  |  |  |
| **IgM N 229E** (median) | 0.21 | 1.28 | 1.31 | 0.386 |
| (P25, P75) | (-3.37, 3.37) | (-1.73, 4.44) | (-2.62, 6.58) |  |
| ∆ ≥15% | 0.0 | 3.6 | 5.1 |  |
| ∇ ≥15% | 0.0 | 1.8 | 0.0 |  |
|  |  |  |  |  |
| **IgM N HKU1** (median) | 1.32 | 0.95 | 0.57 | 0.897 |
| (P25, P75) | (-2.20, 3.11) | (-1.26, 4.37) | (-1.15, 3.65) |  |
| ∆ ≥15% | 1.7 | 1.8 | 2.6 |  |
| ∇ ≥15% | 0.0 | 0.0 | 0.0 |  |
|  |  |  |  |  |
| **IgM NL 63** (median) | 1.28 | 0.93 | 2.42 | 0.240 |
| (P25, P75) | (-2.39, 4.32) | (-3.49, 3.72) | (-0.62, 6.88) |  |
| ∆ ≥15% | 0.0 | 3.6 | 0.0 |  |
| ∇ ≥15% | 1.7 | 0.0 | 0.0 |  |
|  |  |  |  |  |
| **IgM N OC43** (median) | 1.36 | -0.34 | 1.26 | 0.538 |
| (P25, P75) | (-2.70, 3.60) | (-3.01, 4.50) | (-1.70, 5.80) |  |
| ∆ ≥15% | 0.0 | 5.4 | 0.0 |  |
| ∇ ≥15% | 0.0 | 0.0 | 0.0 |  |
|  |  |  |  |  |

[ continued ]

**Supplemental Table 12b.** Continued

|  |  | | |  |
| --- | --- | --- | --- | --- |
| **Immunoglobulin^a^** | **Non-smoker**  (N = 59) | **Former smoker**  (N = 56) | **Current smoker**  (N = 39) | **P^b^** |
| **Total immunoglobulin** |  |  |  |  |
| **IgG1**  (median) | 0.06 | 1.13 | 1.66 | 0.065 |
| (P25, P75) | (-2.55, 2.36) | (-1.32, 3.57) | (0.14, 3.42) |  |
| ∆ ≥15% | 0.0 | 0.0 | 0.0 |  |
| ∇ ≥15% | 0.0 | 0.0 | 0.0 |  |
|  |  |  |  |  |
| **IgG2** (median) | 0.79 | 0.41 | 0.87 | 0.608 |
| (P25, P75) | (-0.47, 2.03) | (-0.75, 2.50) | (-0.63, 2.04) |  |
| ∆ ≥15% | 0.0 | 0.0 | 0.0 |  |
| ∇ ≥15% | 0.0 | 0.0 | 0.0 |  |
|  |  |  |  |  |
| **IgG3** (median) | -0.28 | 0.11 | -0.91 | 0.569 |
| (P25, P75) | (-2.41, 1.65) | (-3.14, 2.24) | (-3.05, 1.45) |  |
| ∆ ≥15% | 0.0 | 0.0 | 0.0 |  |
| ∇ ≥15% | 0.0 | 0.0 | 0.0 |  |
|  |  |  |  |  |
| **IgG4** (median) | 1.39 | 2.48 | 0.17 | 0.130 |
| (P25, P75) | (-1.41, 3.94) | (-1.30, 5.51) | (-2.35, 2.94) |  |
| ∆ ≥15% | 1.7 | 1.8 | 0.0 |  |
| ∇ ≥15% | 1.7 | 0.0 | 0.0 |  |
|  |  |  |  |  |
| **Sum of IgGs** (median) | 0.34 | 0.75 | 1.19 | 0.085 |
| (P25, P75) | (-1.97, 1.99) | (-0.73, 3.07) | (0.11, 2.39) |  |
| ∆ ≥15% | 0.0 | 0.0 | 0.0 |  |
| ∇ ≥15% | 0.0 | 0.0 | 0.0 |  |
|  |  |  |  |  |
| **IgE** (median) | 1.90 | 4.30 | 2.57 | 0.434 |
| (P25, P75) | (-4.62, 9.61) | (-2.57, 12.90) | (-3.86, 8.94) |  |
| ∆ ≥15% | 13.6 | 21.4 | 15.4 |  |
| ∇ ≥15% | 10.2 | 10.7 | 5.1 |  |
|  |  |  |  |  |
| **IgA** (median) | 0.53 | 1.50 | 1.36 | 0.115 |
| (P25, P75) | (-2.42, 2.18) | (-1.87, 5.26) | (-0.84, 4.94) |  |
| ∆ ≥15% | 0.0 | 1.8 | 0.0 |  |
| ∇ ≥15% | 0.0 | 0.0 | 0.0 |  |
|  |  |  |  |  |
| **IgM** (median) | -0.23 | -0.23 | 1.20 | 0.329 |
| (P25, P75) | (-2.03, 3.40) | (-1.52, 3.15) | (-0.90, 3.53) |  |
| ∆ ≥15% | 0.0 | 0.0 | 0.0 |  |
| ∇ ≥15% | 0.0 | 0.0 | 0.0 |  |
|  |  |  |  |  |

P25: percentile 25th. P75: percentile 75th.

Tobacco smoking groups are based on participants’ data in 2020-21.

^a^ Units used for computing the relative intraindividual change were base 10 logtransformed MFI for levels of isotype-antigen combinations, and base 10 logtransformed µg/mL for levels of total Igs.

^b^ Kruskal-Wallis test (two-tailed).

**Supplemental Table 13.** Relative intraindividual change (%) from 2016-17 to 2020-21 of concentrations of 24 isotype-antigen combinations for cytomegalovirus, Epstein-Barr and common cold infections, and of total Igs, by presence or absence of dyslipidemia (N=154).

|  | **Dyslipidemia** | |  |  |
| --- | --- | --- | --- | --- |
| **Immunoglobulin^a^** | **No**  (N = 117) | **Yes**  (N = 37) | **P^b^** |  |
| **IgA** |  |  |  |  |
| **IgA CMV pp150** (median) | 0.26 | 1.16 | 0.511 |  |
| (P25, P75) | (-2.69, 3.38) | (-1.83, 3.86) |  |  |
| ∆ ≥15% | 6 | 0.0 |  |  |
| ∇ ≥15% | 0.9 | 0.0 |  |  |
|  |  |  |  |  |
| **IgA CMV pp65** (median) | 1.01 | 0.85 | 0.948 |  |
| (P25, P75) | (-2.30, 3.50) | (-2.16, 4.98) |  |  |
| ∆ ≥15% | 2.6 | 0.0 |  |  |
| ∇ ≥15% | 0.0 | 2.7 |  |  |
|  |  |  |  |  |
| **IgA EBV EAD** (median) | 0.23 | 0.63 | 0.743 |  |
| (P25, P75) | (-2.21, 3.54) | (-2.50, 5.08) |  |  |
| ∆ ≥15% | 1.7 | 0.0 |  |  |
| ∇ ≥15% | 0.9 | 2.7 |  |  |
|  |  |  |  |  |
| **IgA VCAp18** (median) | 0.57 | 1.24 | 0.533 |  |
| (P25, P75) | (-2.06, 3.11) | (-2.64, 4.18) |  |  |
| ∆ ≥15% | 1.7 | 2.7 |  |  |
| ∇ ≥15% | 0.0 | 2.7 |  |  |
|  |  |  |  |  |
| **IgA N 229E** (median) | 0.14 | -0.98 | 0.538 |  |
| (P25, P75) | (-3.28, 3.39) | (-5.61, 5.33) |  |  |
| ∆ ≥15% | 3.4 | 0.0 |  |  |
| ∇ ≥15% | 4.3 | 8.1 |  |  |
|  |  |  |  |  |
| **IgA N HKU1** (median) | 0.90 | 1.65 | 0.495 |  |
| (P25, P75) | (-1.99, 2.68) | (-2.71, 3.61) |  |  |
| ∆ ≥15% | 2.6 | 2.7 |  |  |
| ∇ ≥15% | 0.0 | 0.0 |  |  |
|  |  |  |  |  |
| **IgA NL 63** (median) | -0.41 | -0.53 | 0.864 |  |
| (P25, P75) | (-3.71, 4.25) | (-3.78, 6.27) |  |  |
| ∆ ≥15% | 7.7 | 2.7 |  |  |
| ∇ ≥15% | 4.3 | 5.4 |  |  |
|  |  |  |  |  |
| **IgA N OC43** (median) | 0.40 | 0.37 | 0.655 |  |
| (P25, P75) | (-2.68, 3.35) | (-3.96, 2.53) |  |  |
| ∆ ≥15% | 3.4 | 0.0 |  |  |
| ∇ ≥15% | 0.0 | 2.7 |  |  |
|  |  |  |  |  |

[ continued ]

**Supplemental Table 13.** Continued

|  | **Dyslipidemia** | |  |  |
| --- | --- | --- | --- | --- |
| **Immunoglobulin^a^** | **No**  (N = 117) | **Yes**  (N = 37) |  |  |
| **IgG** |  |  |  |  |
| **IgG CMV pp150** (median) | 1.66 | 0.80 | 0.552 |  |
| (P25, P75) | (-1.01, 3.93) | (-2.48, 4.90) |  |  |
| ∆ ≥15% | 2.6 | 0.0 |  |  |
| ∇ ≥15% | 0.0 | 0.0 |  |  |
|  |  |  |  |  |
| **IgG CMV pp65** (median) | 0.92 | 0.31 | 0.497 |  |
| (P25, P75) | (-1.65, 2.92) | (-4.08, 5.46) |  |  |
| ∆ ≥15% | 0.9 | 0.0 |  |  |
| ∇ ≥15% | 0.0 | 0.0 |  |  |
|  |  |  |  |  |
| **IgG EBV EAD** (median) | 0.75 | -0.61 | 0.151 |  |
| (P25, P75) | (-1.84, 3.13) | (-3.39, 2.40) |  |  |
| ∆ ≥15% | 0.0 | 0.0 |  |  |
| ∇ ≥15% | 0.0 | 0.0 |  |  |
|  |  |  |  |  |
| **IgG VCAp18** (median) | 0.77 | 0.41 | 0.677 |  |
| (P25, P75) | (-1.69, 3.40) | (-2.34, 3.12) |  |  |
| ∆ ≥15% | 2.6 | 0.0 |  |  |
| ∇ ≥15% | 0.9 | 0.0 |  |  |
|  |  |  |  |  |
| **IgG N 229E** (median) | 0.30 | -0.45 | 0.671 |  |
| (P25, P75) | (-2.42, 2.71) | (-3.17, 2.67) |  |  |
| ∆ ≥15% | 0.9 | 0.0 |  |  |
| ∇ ≥15% | 0.0 | 0.0 |  |  |
|  |  |  |  |  |
| **IgG N HKU1** (median) | 0.69 | 0.18 | 0.155 |  |
| (P25, P75) | (-1.56, 2.51) | (-2.08, 1.70) |  |  |
| ∆ ≥15% | 0.0 | 0.0 |  |  |
| ∇ ≥15% | 0.0 | 0.0 |  |  |
|  |  |  |  |  |
| **IgG NL 63** (median) | 0.10 | -0.39 | 0.740 |  |
| (P25, P75) | (-2.23, 2.59) | (-2.95, 2.72) |  |  |
| ∆ ≥15% | 0.0 | 0.0 |  |  |
| ∇ ≥15% | 0.0 | 0.0 |  |  |
|  |  |  |  |  |
| **IgG N OC43** (median) | 0.24 | -0.63 | 0.455 |  |
| (P25, P75) | (-2.97, 3.00) | (-3.85, 3.38) |  |  |
| ∆ ≥15% | 0.9 | 0.0 |  |  |
| ∇ ≥15% | 1.7 | 5.4 |  |  |
|  |  |  |  |  |

[ continued ]

**Supplemental Table 13.** Continued.

|  | **Dyslipidemia** | |  |  |
| --- | --- | --- | --- | --- |
| **Immunoglobulin^a^** | **No**  (N = 117) | **Yes**  (N = 37) | **P^b^** |  |
| **IgM** |  |  |  |  |
| **IgM CMV pp150** (median) | 0.95 | 1.58 | 0.538 |  |
| (P25, P75) | (-2.10, 3.53) | (-1.70, 5.91) |  |  |
| ∆ ≥15% | 0.9 | 2.7 |  |  |
| ∇ ≥15% | 0.0 | 2.7 |  |  |
|  |  |  |  |  |
| **IgM CMV pp65** (median) | 0.62 | 0.21 | 0.721 |  |
| (P25, P75) | (-1.66, 3.03) | (-2.33, 4.50) |  |  |
| ∆ ≥15% | 0.0 | 2.7 |  |  |
| ∇ ≥15% | 0.0 | 0.0 |  |  |
|  |  |  |  |  |
| **IgM EBV EAD** (median) | 0.45 | 0.55 | 0.340 |  |
| (P25, P75) | (-2.33, 3.11) | (-2.07, 4.38) |  |  |
| ∆ ≥15% | 0.9 | 2.7 |  |  |
| ∇ ≥15% | 0.0 | 0.0 |  |  |
|  |  |  |  |  |
| **IgM VCAp18** (median) | 0.89 | 1.68 | 0.097 |  |
| (P25, P75) | (-2.53, 3.89) | (0.03, 5.94) |  |  |
| ∆ ≥15% | 1.7 | 2.7 |  |  |
| ∇ ≥15% | 0.0 | 0.0 |  |  |
|  |  |  |  |  |
| **IgM N 229E** (median) | 0.40 | 2.97 | 0.050 |  |
| (P25, P75) | (-3.29, 4.08) | (-1.63, 7.92) |  |  |
| ∆ ≥15% | 2.6 | 2.7 |  |  |
| ∇ ≥15% | 0.9 | 0.0 |  |  |
|  |  |  |  |  |
| **IgM N HKU1** (median) | 0.61 | 1.77 | 0.054 |  |
| (P25, P75) | (-1.92, 3.12) | (-0.64, 6.39) |  |  |
| ∆ ≥15% | 0.9 | 5.4 |  |  |
| ∇ ≥15% | 0.0 | 0.0 |  |  |
|  |  |  |  |  |
| **IgM NL 63** (median) | 0.90 | 2.24 | 0.204 |  |
| (P25, P75) | (-2.89, 4.21) | (-2.37, 6.52) |  |  |
| ∆ ≥15% | 0.9 | 2.7 |  |  |
| ∇ ≥15% | 0.9 | 0.0 |  |  |
|  |  |  |  |  |
| **IgM N OC43** (median) | -0.19 | 3.26 | 0.004 |  |
| (P25, P75) | (-3.27, 3.29) | (-1.06, 6.91) |  |  |
| ∆ ≥15% | 1.7 | 2.7 |  |  |
| ∇ ≥15% | 0.0 | 0.0 |  |  |
|  |  |  |  |  |

[ continued ]

**Supplemental Table 13.** Continued.

|  | **Dyslipidemia** | |  |  |
| --- | --- | --- | --- | --- |
| **Immunoglobulin^a^** | **No**  (N = 117) | **Yes**  (N = 37) | **P^b^** |  |
| **Total Immunoglobulin** |  |  |  |  |
| **IgG1** (median) | 0.84 | 1.96 | 0.050 |  |
| (P25, P75) | (-1.78, 2.96) | (-0.07, 4.20) |  |  |
| ∆ ≥15% | 0.0 | 0.0 |  |  |
| ∇ ≥15% | 0.0 | 0.0 |  |  |
|  |  |  |  |  |
| **IgG2** (median) | 0.51 | 0.79 | 0.071 |  |
| (P25, P75) | (-0.97, 1.99) | (0.29, 2.76) |  |  |
| ∆ ≥15% | 0.0 | 0.0 |  |  |
| ∇ ≥15% | 0.0 | 0.0 |  |  |
|  |  |  |  |  |
| **IgG3** (median) | -0.43 | 0.18 | 0.046 |  |
| (P25, P75) | (-3.09, 1.21) | (-1.18, 2.21) |  |  |
| ∆ ≥15% | 0.0 | 0.0 |  |  |
| ∇ ≥15% | 0.0 | 0.0 |  |  |
|  |  |  |  |  |
| **IgG4** (median) | 0.36 | 2.92 | 0.016 |  |
| (P25, P75) | (-2.07, 3.83) | (-0.07, 5.97) |  |  |
| ∆ ≥15% | 0.9 | 2.7 |  |  |
| ∇ ≥15% | 0.9 | 0.0 |  |  |
|  |  |  |  |  |
| **Sum of IgGs** (median) | 0.55 | 1.65 | 0.032 |  |
| (P25, P75) | (-1.49, 2.23) | (0.11, 2.98) |  |  |
| ∆ ≥15% | 0.0 | 0.0 |  |  |
| ∇ ≥15% | 0.0 | 0.0 |  |  |
|  |  |  |  |  |
| **IgE** (median) | 2.57 | 3.98 | 0.338 |  |
| (P25, P75) | (-5.08, 10.67) | (-1.34, 13.31) |  |  |
| ∆ ≥15% | 16.2 | 18.9 |  |  |
| ∇ ≥15% | 11.1 | 2.7 |  |  |
|  |  |  |  |  |
| **IgA** (median) | 0.16 | 3.09 | 0.002 |  |
| (P25, P75) | (-2.61, 3.23) | (0.68, 5.20) |  |  |
| ∆ ≥15% | 0.9 | 0.0 |  |  |
| ∇ ≥15% | 0.0 | 0.0 |  |  |
|  |  |  |  |  |
| **IgM** (median) | -0.18 | 1.73 | 0.046 |  |
| (P25, P75) | (-1.67, 2.87) | (-0.85, 4.35) |  |  |
| ∆ ≥15% | 0.0 | 0.0 |  |  |
| ∇ ≥15% | 0.0 | 0.0 |  |  |
|  |  |  |  |  |

P25: percentile 25th. P75: percentile 75th.

The presence or absence of dyslipidemia was determined from data provided by the Data Analytics Program for Health Research and Innovation (PADRIS) and the Barcelona Health Survey (BHS) of 2016 (section 2.7). The following ICD-10 codes were used: E78.00 (pure hypercholesterolemia, unspecified, N=19), E78.1 (pure hyperglyceridemia, N=3), E78.2 (mixed hyperlipidemia, N=1), E78.5 (hyperlipidemia, unspecified, N=3), and E78.9 (disorder of lipoprotein metabolism, unspecified, N=11).

^a^ Units used for computing the relative intraindividual change were base 10 logtransformed MFI for levels of isotype-antigen combinations, and base 10 logtransformed µg/mL for levels of total Igs.

^b^ Mann-Whitney’s *U* test (two-tail).

**Supplemental Table 14a.** Concentrations of 24 isotype-antigen combinations for cytomegalovirus, Epstein-Barr and common cold infections, and of total Igs in 2016-17 by educational level (N=240).

|  |  |  |  |  |
| --- | --- | --- | --- | --- |
| **Immunoglobulin**  (MFI) | **Primary or less**  (N = 56) | **Secondary**  (N = 65) | **University**  (N = 119) | **P^a^** |
| **IgA** |  |  |  |  |
| **IgA CMV pp150** (median) | 487.5 | 502.5 | 490.0 | 0.966 |
| (P25, P75) | (337.5, 872.6) | (342.5, 884.5) | (332.0, 821.5) |  |
| Geometric mean | 609.6 | 616.6 | 585.9 |  |
|  |  |  |  |  |
| **IgA CMV pp65** (median) | 592.0 | 535.5 | 599.0 | 0.994 |
| (P25, P75) | (405.6, 1098) | (417.2, 1060) | (408.5, 836.0) |  |
| Geometric mean | 769.9 | 672.6 | 665.0 |  |
|  |  |  |  |  |
| **IgA EBV EAD** (median) | 414.5 | 426.0 | 426.5 | 0.868 |
| (P25, P75) | (288.2, 744.5) | (291.5, 622.2) | (328.0, 636.5) |  |
| Geometric mean | 516.9 | 485.4 | 502.7 |  |
|  |  |  |  |  |
| **IgA VCAp18** (median) | 459.5 | 499.0 | 481.5 | 0.866 |
| (P25, P75) | (333.4, 746.2) | (320.5, 808.2) | (340.0, 730.0) |  |
| Geometric mean | 578.7 | 573.5 | 556.6 |  |
|  |  |  |  |  |
| **IgA N 229E** (median) | 1476 | 1139 | 1420 | 0.438 |
| (P25, P75) | (711.4, 3668) | (679.5, 3412) | (747.5, 4162) |  |
| Geometric mean | 1823 | 1551 | 1916 |  |
|  |  |  |  |  |
| **IgA N HKU1** (median) | 737.8 | 634.0 | 684.0 | 0.731 |
| (P25, P75) | (478.0, 1429) | (436.5, 1143) | (476.0, 1094) |  |
| Geometric mean | 883.7 | 790.6 | 801.0 |  |
|  |  |  |  |  |
| **IgA NL 63**  (median) | 1532 | 1523 | 1680 | 0.835 |
| (P25, P75) | (661.5, 7030) | (790.2, 4409) | (801.0, 4550) |  |
| Geometric mean | 1991 | 2003 | 2135 |  |
|  |  |  |  |  |
| **IgA N OC43** (median) | 1328 | 1544 | 1377 | 0.610 |
| (P25, P75) | (784.5, 2617) | (945.5, 2699) | (900.5, 3005) |  |
| Geometric mean | 1441 | 1684 | 1715 |  |
|  |  |  |  |  |

[ continued ]

**Supplemental Table 14a.** Continued.

|  |  |  |  |  |
| --- | --- | --- | --- | --- |
| **Immunoglobulin**  (MFI) | **Primary or less**  (N = 56) | **Secondary**  (N = 65) | **University**  (N = 119) | **P^a^** |
| **IgG** |  |  |  |  |
| **IgG CMV pp150** (median) | 17454 | 18278 | 13403 | 0.045 |
| (P25, P75) | (6094, 32650) | (8736, 44660) | (4962, 24583) |  |
| Geometric mean | 15082 | 17448 | 11291 |  |
|  |  |  |  |  |
| **IgG CMV pp65** (median) | 6114 | 7390 | 6747 | 0.051 |
| (P25, P75) | (3150, 10210) | (5167, 12529) | (4465, 10435) |  |
| Geometric mean | 5995 | 7964 | 6568 |  |
|  |  |  |  |  |
| **IgG EBV EAD** (median) | 5878 | 6983 | 7070 | 0.678 |
| (P25, P75) | (4659, 10964) | (5272, 9630) | (4712, 9901) |  |
| Geometric mean | 6740 | 7209 | 6704 |  |
|  |  |  |  |  |
| **IgG VCAp18** (median) | 7949 | 7699 | 7815 | 0.901 |
| (P25, P75) | (3777, 14112) | (4283, 14120) | (4381, 14461) |  |
| Geometric mean | 7885 | 8545 | 8839 |  |
|  |  |  |  |  |
| **IgG N 229E** (median) | 56887 | 46238 | 43104 | 0.519 |
| (P25, P75) | (23160, 87043) | (24571, 73910) | (26070, 69012) |  |
| Geometric mean | 45839 | 42941 | 39493 |  |
|  |  |  |  |  |
| **IgG N HKU1** (median) | 14313 | 14732 | 13611 | 0.258 |
| (P25, P75) | (9857, 19927) | (11426, 19512) | (9517, 18774) |  |
| Geometric mean | 14479 | 14703 | 12861 |  |
|  |  |  |  |  |
| **IgG NL 63**  (median) | 31365 | 41283 | 36258 | 0.317 |
| (P25, P75) | (18233, 58213) | (25159, 57890) | (20630, 58662) |  |
| Geometric mean | 31003 | 38633 | 33594 |  |
|  |  |  |  |  |
| **IgG N OC43** (median) | 19823 | 25113 | 21367 | 0.385 |
| (P25, P75) | (11482, 40084) | (12352, 39554) | (10710, 36189) |  |
| Geometric mean | 22025 | 23238 | 19564 |  |
|  |  |  |  |  |

[ continued ]

**Supplemental Table 14a.** Continued.

|  |  |  |  |  |
| --- | --- | --- | --- | --- |
| **Immunoglobulin**  (MFI) | **Primary or less**  (N = 56) | **Secondary**  (N = 65) | **University**  (N = 119) | **P^a^** |
| **IgM** |  |  |  |  |
| **IgM CMV pp150** (median) | 1308 | 1129 | 1300 | 0.770 |
| (P25, P75) | (578.5, 2498) | (719.5, 2058) | (798.5, 2539) |  |
| Geometric mean | 1397 | 1315 | 1405 |  |
|  |  |  |  |  |
| **IgM CMV pp65** (median) | 548.8 | 676.0 | 718.0 | 0.323 |
| (P25, P75) | (394.6, 1097) | (514.2, 942.0) | (425.5, 1102) |  |
| Geometric mean | 649.6 | 704.3 | 718.9 |  |
|  |  |  |  |  |
| **IgM EBV EAD** (median) | 412.2 | 432.0 | 415.0 | 0.529 |
| (P25, P75) | (277.6, 583.5) | (334.5, 586.0) | (286.0, 594.0) |  |
| Geometric mean | 414.8 | 458.9 | 433.6 |  |
|  |  |  |  |  |
| **IgM VCAp18** (median) | 641.0 | 884.0 | 895.0 | 0.103 |
| (P25, P75) | (425.5, 1284) | (592.5, 1534) | (517.0, 1780) |  |
| Geometric mean | 756.7 | 985.3 | 952.8 |  |
|  |  |  |  |  |
| **IgM N 229E** (median) | 1380 | 1347 | 1532 | 0.397 |
| (P25, P75) | (832.1, 2425) | (863.8, 1888) | (928.0, 2638) |  |
| Geometric mean | 1390 | 1363 | 1518 |  |
|  |  |  |  |  |
| **IgM N HKU1** (median) | 1160 | 1022 | 987.5 | 0.902 |
| (P25, P75) | (682.8, 1708) | (747.8, 1482) | (713.0, 1654) |  |
| Geometric mean | 1126 | 1051 | 1072 |  |
|  |  |  |  |  |
| **IgM NL 63**  (median) | 1036 | 1029 | 930.5 | 0.748 |
| (P25, P75) | (523.2, 1509) | (579.8, 1862) | (597.5, 1541) |  |
| Geometric mean | 966.2 | 1094 | 990.3 |  |
|  |  |  |  |  |
| **IgM N OC43** (median) | 2110 | 2378 | 1980 | 0.648 |
| (P25, P75) | (997.2, 3411) | (1132, 3600) | (1126, 3562) |  |
| Geometric mean | 1964 | 2220 | 2031 |  |
|  |  |  |  |  |

[ continued ]

**Supplemental Table 14a.** Continued.

|  |  |  |  |  |
| --- | --- | --- | --- | --- |
| **Immunoglobulin**  (µg/mL) | **Primary or less**  (N = 56) | **Secondary**  (N = 65) | **University**  (N = 119) | **P^a^** |
| **Total immunoglobulin** |  |  |  |  |
| **IgG1** (median) | 5970 | 6707 | 5848 | 0.059 |
| (P25, P75) | (4380, 7728) | (5109, 8955) | (4592, 7940) |  |
| Geometric mean | 6110 | 6881 | 5993 |  |
|  |  |  |  |  |
| **IgG2** (median) | 2918 | 3033 | 3192 | 0.595 |
| (P25, P75) | (2506, 3601) | (2507, 3530) | (2436, 3752) |  |
| Geometric mean | 2978 | 3043 | 3067 |  |
|  |  |  |  |  |
| **IgG3** (median) | 1272 | 1235 | 1236 | 0.783 |
| (P25, P75) | (1022, 1557) | (990.0, 1438) | (1011, 1472) |  |
| Geometric mean | 1199 | 1187 | 1204 |  |
|  |  |  |  |  |
| **IgG4** (median) | 201.4 | 222.7 | 258.0 | 0.659 |
| (P25, P75) | (112.9, 387.1) | (132.2, 474.1) | (118.4, 377.1) |  |
| Geometric mean | 214.5 | 232.0 | 229.8 |  |
|  |  |  |  |  |
| **Sum of IgGs** (median) | 10888 | 11833 | 10760 | 0.124 |
| (P25, P75) | (8659, 13751) | (9414, 14743) | (8545, 13370) |  |
| Geometric mean | 10840 | 11698 | 10779 |  |
|  |  |  |  |  |
| **IgE** (median) | 0.21 | 0.26 | 0.23 | 0.685 |
| (P25, P75) | (0.14, 0.36) | (0.15, 0.36) | (0.15, 0.33) |  |
| Geometric mean | 0.23 | 0.24 | 0.22 |  |
|  |  |  |  |  |
| **IgA** (median) | 241.1 | 254.9 | 245.4 | 0.738 |
| (P25, P75) | (175.8, 298.5) | (194.9, 310.1) | (194.3, 297.1) |  |
| Geometric mean | 239.2 | 244.9 | 237.6 |  |
|  |  |  |  |  |
| **IgM** (median) | 885.4 | 916.8 | 946.5 | 0.340 |
| (P25, P75) | (613.3, 1198) | (755.8, 1220) | (688.7, 1382) |  |
| Geometric mean | 875.8 | 972.2 | 967.8 |  |
|  |  |  |  |  |

P25: percentile 25th. P75: percentile 75th.

<LOQ: value lower than the minimum concentration of the corresponding range of limits of quantification.

See Supplemental Table 1.

^a^ Kruskal-Wallis test (two-tailed).

**Supplemental Table 14b.** Relative intraindividual change (%) from 2016-17 to 2020-21 of concentrations of 24 isotype-antigen combinations for cytomegalovirus, Epstein-Barr and common cold infections, and of total Igs, by educational level (N=154).

|  |  | | |  |
| --- | --- | --- | --- | --- |
| **Immunoglobulin^a^** | **Primary or less** (N = 35) | **Secondary**  (N = 42) | **University**  (N = 77) | **P^b^** |
| **IgA** |  |  |  |  |
| **IgA CMV pp150** (median) | 1.12 | 0.67 | 0.00 | 0.513 |
| (P25, P75) | (-2.23, 4.79) | (-2.06, 3.43) | (-2.82, 2.82) |  |
| ∆ ≥15% | 2.9 | 4.8 | 5.2 |  |
| ∇ ≥15% | 2.9 | 0.0 | 0.0 |  |
|  |  |  |  |  |
| **IgA CMV pp65** (median) | 1.74 | 0.38 | 0.74 | 0.157 |
| (P25, P75) | (-0.83, 6.87) | (-3.25, 2.73) | (-2.29, 3.50) |  |
| ∆ ≥15% | 0.0 | 2.4 | 2.6 |  |
| ∇ ≥15% | 0.0 | 2.4 | 0.0 |  |
|  |  |  |  |  |
| **IgA EBV EAD** (median) | 0.75 | -0.35 | 0.45 | 0.437 |
| (P25, P75) | (-0.96, 4.45) | (-2.98, 3.79) | (-2.31, 3.19) |  |
| ∆ ≥15% | 0.0 | 0.0 | 2.6 |  |
| ∇ ≥15% | 0.0 | 2.4 | 1.3 |  |
|  |  |  |  |  |
| **IgA VCAp18** (median) | 1.47 | -0.61 | 1.04 | 0.143 |
| (P25, P75) | (-1.43, 3.85) | (-3.33, 3.17) | (-2.00, 3.58) |  |
| ∆ ≥15% | 2.9 | 0.0 | 2.6 |  |
| ∇ ≥15% | 0.0 | 2.4 | 0.0 |  |
|  |  |  |  |  |
| **IgA N 229E** (median) | 1.36 | -0.21 | -0.74 | 0.963 |
| (P25, P75) | (-3.77, 3.07) | (-4.92, 5.53) | (-3.53, 3.46) |  |
| ∆ ≥15% | 0.0 | 4.8 | 2.6 |  |
| ∇ ≥15% | 8.6 | 4.8 | 3.9 |  |
|  |  |  |  |  |
| **IgA N HKU1** (median) | 1.97 | 0.29 | 0.76 | 0.385 |
| (P25, P75) | (-1.47, 3.96) | (-2.94, 3.46) | (-2.52, 2.68) |  |
| ∆ ≥15% | 2.9 | 2.4 | 2.6 |  |
| ∇ ≥15% | 0.0 | 0.0 | 0.0 |  |
|  |  |  |  |  |
| **IgA NL 63** (median) | 0.11 | -1.02 | -0.52 | 0.454 |
| (P25, P75) | (-1.92, 6.60) | (-4.97, 6.78) | (-3.70, 3.74) |  |
| ∆ ≥15% | 8.6 | 9.5 | 3.9 |  |
| ∇ ≥15% | 0.0 | 7.1 | 5.2 |  |
|  |  |  |  |  |
| **IgA N OC43** (median) | -0.22 | -0.16 | 0.85 | 0.641 |
| (P25, P75) | (-3.29, 2.38) | (-2.71, 3.94) | (-2.58, 3.77) |  |
| ∆ ≥15% | 0.0 | 4.8 | 2.6 |  |
| ∇ ≥15% | 2.9 | 0.0 | 0.0 |  |
|  |  |  |  |  |

[ continued ]

**Supplemental Table 14b.** Continued

|  |  | | |  |
| --- | --- | --- | --- | --- |
| **Immunoglobulin^a^** | **Primary or less** (N = 35) | **Secondary**  (N = 42) | **University**  (N = 77) | **P^b^** |
| **IgG** |  |  |  |  |
| **IgG CMV pp150** (median) | 1.22 | 0.90 | 1.55 | 0.939 |
| (P25, P75) | (-3.19, 4.66) | (-0.52, 3.99) | (-1.60, 3.83) |  |
| ∆ ≥15% | 2.9 | 2.4 | 1.3 |  |
| ∇ ≥15% | 0.0 | 0.0 | 0.0 |  |
|  |  |  |  |  |
| **IgG CMV pp65** (median) | 0.90 | 1.08 | 0.79 | 0.683 |
| (P25, P75) | (-2.56, 4.31) | (-1.79, 2.55) | (-2.88, 2.47) |  |
| ∆ ≥15% | 0.0 | 2.4 | 0.0 |  |
| ∇ ≥15% | 0.0 | 0.0 | 0.0 |  |
|  |  |  |  |  |
| **IgG EBV EAD** (median) | 0.24 | 1.02 | -0.18 | 0.591 |
| (P25, P75) | (-2.27, 3.17) | (-1.98, 3.17) | (-2.26, 2.40) |  |
| ∆ ≥15% | 0.0 | 0.0 | 0.0 |  |
| ∇ ≥15% | 0.0 | 0.0 | 0.0 |  |
|  |  |  |  |  |
| **IgG VCAp18** (median) | 0.42 | 1.38 | 0.41 | 0.180 |
| (P25, P75) | (-2.29, 4.28) | (-0.26, 4.97) | (-2.35, 2.61) |  |
| ∆ ≥15% | 0.0 | 4.8 | 1.3 |  |
| ∇ ≥15% | 0.0 | 0.0 | 1.3 |  |
|  |  |  |  |  |
| **IgG N 229E** (median) | -0.78 | 0.54 | 0.15 | 0.565 |
| (P25, P75) | (-3.58, 2.96) | (-1.79, 2.67) | (-2.64, 2.59) |  |
| ∆ ≥15% | 0.0 | 0.0 | 0.0 |  |
| ∇ ≥15% | 0.0 | 0.0 | 0.0 |  |
|  |  |  |  |  |
| **IgG N HKU1** (median) | -0.05 | 0.94 | 0.60 | 0.624 |
| (P25, P75) | (-1.59, 2.46) | (-1.49, 2.65) | (-1.72, 1.90) |  |
| ∆ ≥15% | 0.0 | 0.0 | 0.0 |  |
| ∇ ≥15% | 0.0 | 0.0 | 0.0 |  |
|  |  |  |  |  |
| **IgG NL 63** (median) | -0.47 | 0.14 | 0.35 | 0.830 |
| (P25, P75) | (-1.89, 2.87) | (-2.25, 3.11) | (-2.90, 2.32) |  |
| ∆ ≥15% | 0.0 | 0.0 | 0.0 |  |
| ∇ ≥15% | 0.0 | 0.0 | 0.0 |  |
|  |  |  |  |  |
| **IgG N OC43** (median) | 0.52 | 0.15 | -0.13 | 0.669 |
| (P25, P75) | (-4.17, 3.16) | (-2.18, 3.45) | (-3.89, 2.96) |  |
| ∆ ≥15% | 0.0 | 0.0 | 1.3 |  |
| ∇ ≥15% | 5.7 | 2.4 | 1.3 |  |
|  |  |  |  |  |

[ continued ]

**Supplemental Table 14b.** Continued

|  |  | | |  |
| --- | --- | --- | --- | --- |
| **Immunoglobulin^a^** | **Primary or less** (N = 35) | **Secondary**  (N = 42) | **University**  (N = 77) | **P^b^** |
| **IgM** |  |  |  |  |
| **IgM CMV pp150** (median) | -0.18 | 1.99 | 1.22 | 0.444 |
| (P25, P75) | (-1.48, 3.71) | (-1.69, 4.53) | (-2.50, 3.45) |  |
| ∆ ≥15% | 2.9 | 2.4 | 0.0 |  |
| ∇ ≥15% | 0.0 | 2.4 | 0.0 |  |
|  |  |  |  |  |
| **IgM CMV pp65** (median) | 0.82 | -0.03 | 0.66 | 0.674 |
| (P25, P75) | (-2.63, 4.22) | (-2.68, 3.62) | (-1.39, 3.66) |  |
| ∆ ≥15% | 0.0 | 0.0 | 1.3 |  |
| ∇ ≥15% | 0.0 | 0.0 | 0.0 |  |
|  |  |  |  |  |
| **IgM EBV EAD** (median) | 1.23 | 0.52 | 0.38 | 0.776 |
| (P25, P75) | (-1.68, 3.58) | (-1.96, 4.12) | (-2.92, 3.67) |  |
| ∆ ≥15% | 0.0 | 2.4 | 1.3 |  |
| ∇ ≥15% | 0.0 | 0.0 | 0.0 |  |
|  |  |  |  |  |
| **IgM VCAp18** (median) | 0.89 | 1.57 | 0.98 | 0.692 |
| (P25, P75) | (-2.37, 6.56) | (-0.70, 3.97) | (-2.56, 3.51) |  |
| ∆ ≥15% | 2.9 | 0.0 | 2.6 |  |
| ∇ ≥15% | 0.0 | 0.0 | 0.0 |  |
|  |  |  |  |  |
| **IgM N 229E** (median) | 1.90 | 1.27 | -0.02 | 0.540 |
| (P25, P75) | (-2.46, 4.79) | (-1.86, 4.03) | (-3.41, 4.25) |  |
| ∆ ≥15% | 2.9 | 2.4 | 2.6 |  |
| ∇ ≥15% | 0.0 | 0.0 | 1.3 |  |
|  |  |  |  |  |
| **IgM N HKU1** (median) | 1.32 | 0.95 | 0.61 | 0.722 |
| (P25, P75) | (-0.76, 4.32) | (-1.01, 4.18) | (-1.89, 3.57) |  |
| ∆ ≥15% | 2.9 | 2.4 | 1.3 |  |
| ∇ ≥15% | 0.0 | 0.0 | 0.0 |  |
|  |  |  |  |  |
| **IgM NL 63** (median) | 0.99 | 1.12 | 1.26 | 0.543 |
| (P25, P75) | (-3.17, 3.90) | (-2.21, 6.08) | (-3.39, 4.06) |  |
| ∆ ≥15% | 0.0 | 4.8 | 0.0 |  |
| ∇ ≥15% | 2.9 | 0.0 | 0.0 |  |
|  |  |  |  |  |
| **IgM N OC43** (median) | 1.34 | 0.67 | 0.43 | 0.830 |
| (P25, P75) | (-3.21, 5.42) | (-2.50, 4.37) | (-2.65, 3.77) |  |
| ∆ ≥15% | 2.9 | 2.4 | 1.3 |  |
| ∇ ≥15% | 0.0 | 0.0 | 0.0 |  |
|  |  |  |  |  |

[ continued ]

**Supplemental Table 14b.** Continued

|  |  | | |  |
| --- | --- | --- | --- | --- |
| **Immunoglobulin^a^** | **Primary or less** (N = 35) | **Secondary**  (N = 42) | **University**  (N = 77) | **P^b^** |
| **Total immunoglobulin** |  |  |  |  |
| **IgG1**  (median) | 1.43 | 0.54 | 1.26 | 0.835 |
| (P25, P75) | (-2.40, 3.73) | (-1.58, 3.06) | (-1.47, 3.14) |  |
| ∆ ≥15% | 0.0 | 0.0 | 0.0 |  |
| ∇ ≥15% | 0.0 | 0.0 | 0.0 |  |
|  |  |  |  |  |
| **IgG2** (median) | 0.83 | 0.41 | 0.68 | 0.967 |
| (P25, P75) | (-1.91, 2.04) | (-0.71, 2.79) | (-0.43, 1.98) |  |
| ∆ ≥15% | 0.0 | 0.0 | 0.0 |  |
| ∇ ≥15% | 0.0 | 0.0 | 0.0 |  |
|  |  |  |  |  |
| **IgG3** (median) | -0.74 | -0.09 | -0.28 | 0.630 |
| (P25, P75) | (-2.67, 1.34) | (-3.36, 2.51) | (-1.89, 0.86) |  |
| ∆ ≥15% | 0.0 | 0.0 | 0.0 |  |
| ∇ ≥15% | 0.0 | 0.0 | 0.0 |  |
|  |  |  |  |  |
| **IgG4** (median) | 2.03 | 1.76 | 0.75 | 0.984 |
| (P25, P75) | (-3.17, 4.06) | (-1.39, 4.48) | (-1.72, 4.76) |  |
| ∆ ≥15% | 2.9 | 2.4 | 0.0 |  |
| ∇ ≥15% | 2.9 | 0.0 | 0.0 |  |
|  |  |  |  |  |
| **Sum of IgGs** (median) | 0.73 | 0.58 | 0.85 | 0.975 |
| (P25, P75) | (-1.61, 2.46) | (-0.91, 2.45) | (-1.01, 2.20) |  |
| ∆ ≥15% | 0.0 | 0.0 | 0.0 |  |
| ∇ ≥15% | 0.0 | 0.0 | 0.0 |  |
|  |  |  |  |  |
| **IgE** (median) | 2.91 | 3.35 | 1.90 | 0.682 |
| (P25, P75) | (-2.35, 12.49) | (-0.68, 12.02) | (-5.70, 10.56) |  |
| ∆ ≥15% | 17.1 | 19 | 15.6 |  |
| ∇ ≥15% | 2.9 | 11.9 | 10.4 |  |
|  |  |  |  |  |
| **IgA** (median) | 2.02 | 0.80 | 0.74 | 0.486 |
| (P25, P75) | (-1.43, 4.94) | (-3.12, 4.05) | (-1.68, 3.61) |  |
| ∆ ≥15% | 0.0 | 0.0 | 1.3 |  |
| ∇ ≥15% | 0.0 | 0.0 | 0.0 |  |
|  |  |  |  |  |
| **IgM** (median) | -0.15 | 0.18 | 0.35 | 0.819 |
| (P25, P75) | (-1.56, 3.04) | (-1.63, 4.10) | (-1.33, 3.00) |  |
| ∆ ≥15% | 0.0 | 0.0 | 0.0 |  |
| ∇ ≥15% | 0.0 | 0.0 | 0.0 |  |
|  |  |  |  |  |

P25: percentile 25th. P75: percentile 75th.

Educational levels groups are based on participants’ data in 2020-21.

^a^ Units used for computing the relative intraindividual change were base 10 logtransformed MFI for levels of isotype-antigen combinations, and base 10 logtransformed µg/mL for levels of total Igs.

^b^ Kruskal-Wallis test (two-tailed).

**Supplemental Figure 1**. Correlations between concentrations of cytokines and immunoglobulins

in 2016-2017.

Spearman’s ρ (rho)


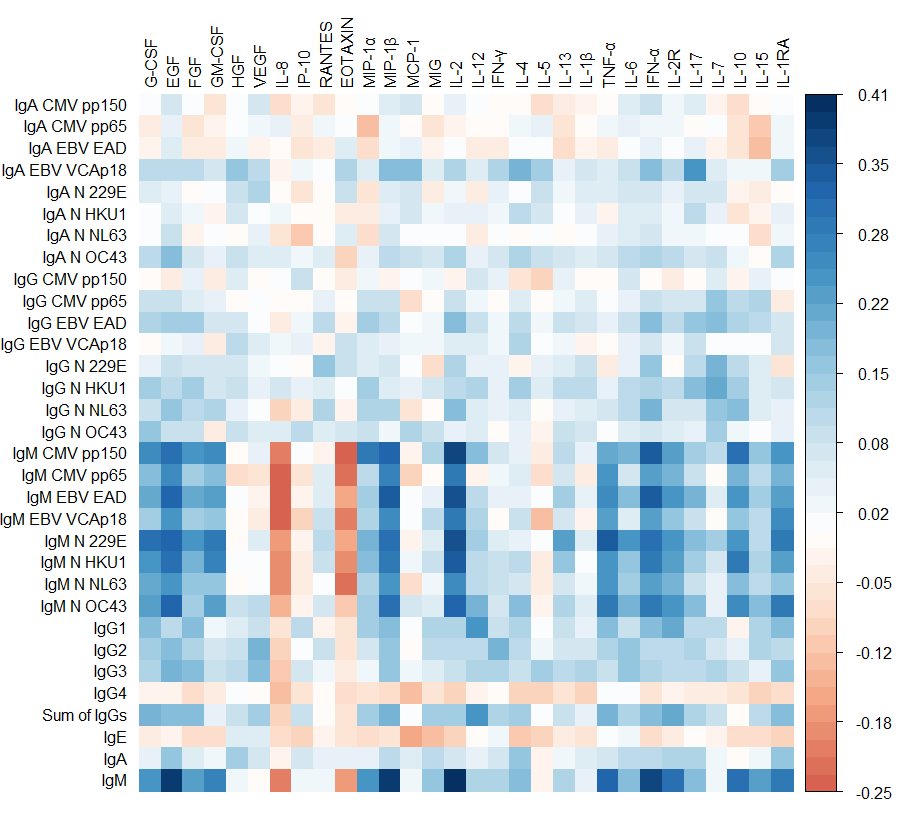


**Supplemental Figure 2**. Correlations between concentrations of cytokines in 2016-2017.


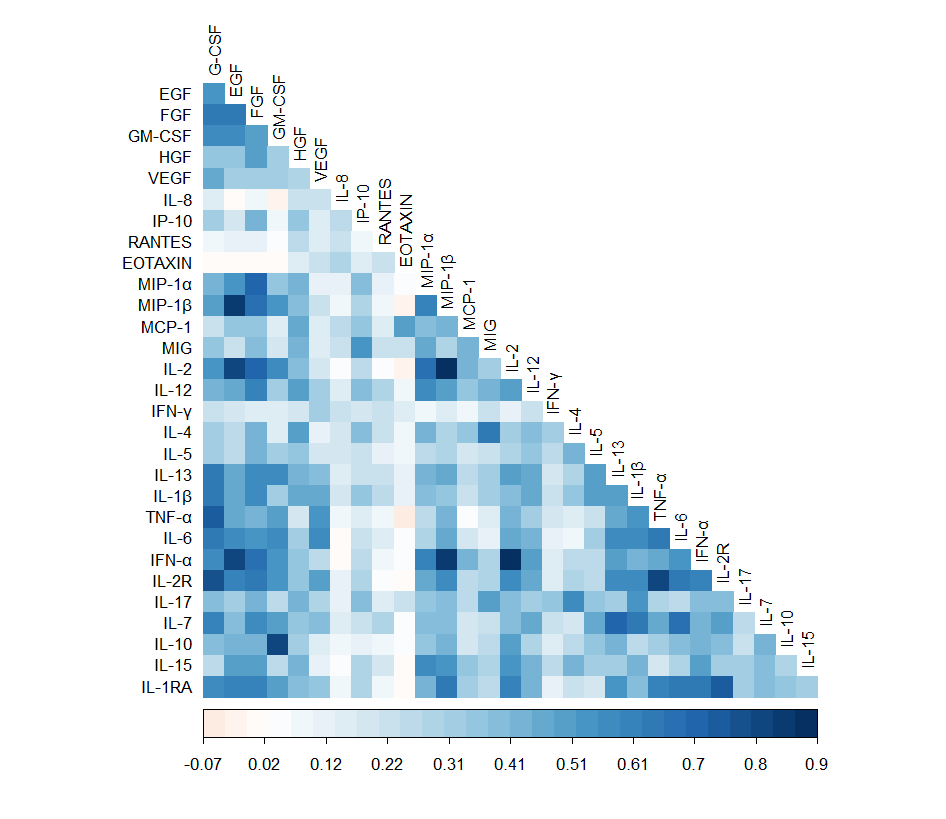


Spearman’s ρ (rho)

**Supplemental Figure 3.** Correlations between concentrations of immunoglobulins against CMV, EBV and HCoV, and total immunoglobulins in 2016-2017.


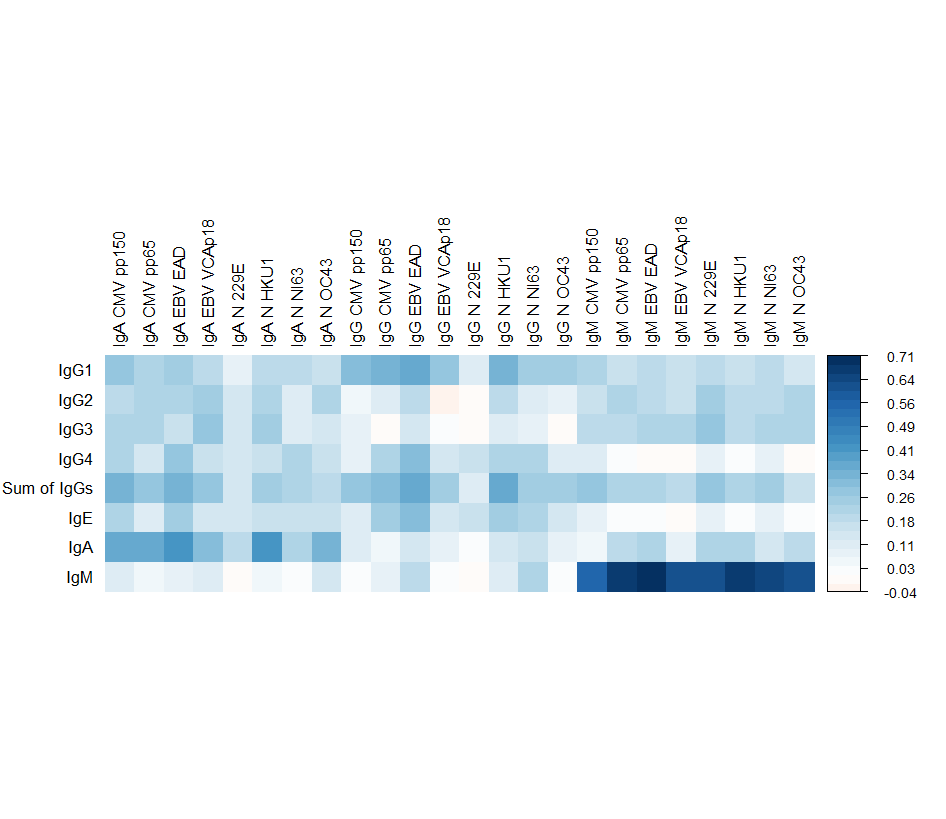


Spearman’s ρ (rho)

**Supplemental Figure 4a.** Correlations between concentrations of cytokines and comorbidities in 2016-2017.


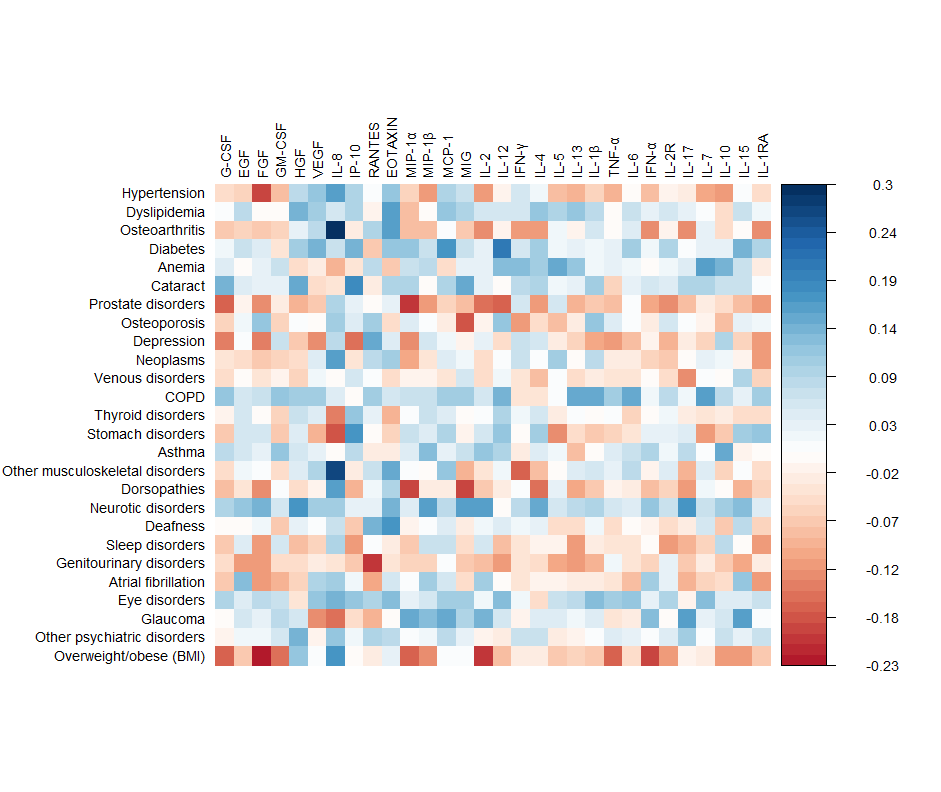


Spearman’s ρ (rho)

**Supplemental Figure 4b.** Correlations between concentrations of immunoglobulins against CMV, EBV and HCoV, total immunoglobulins and comorbidities in 2016-2017.


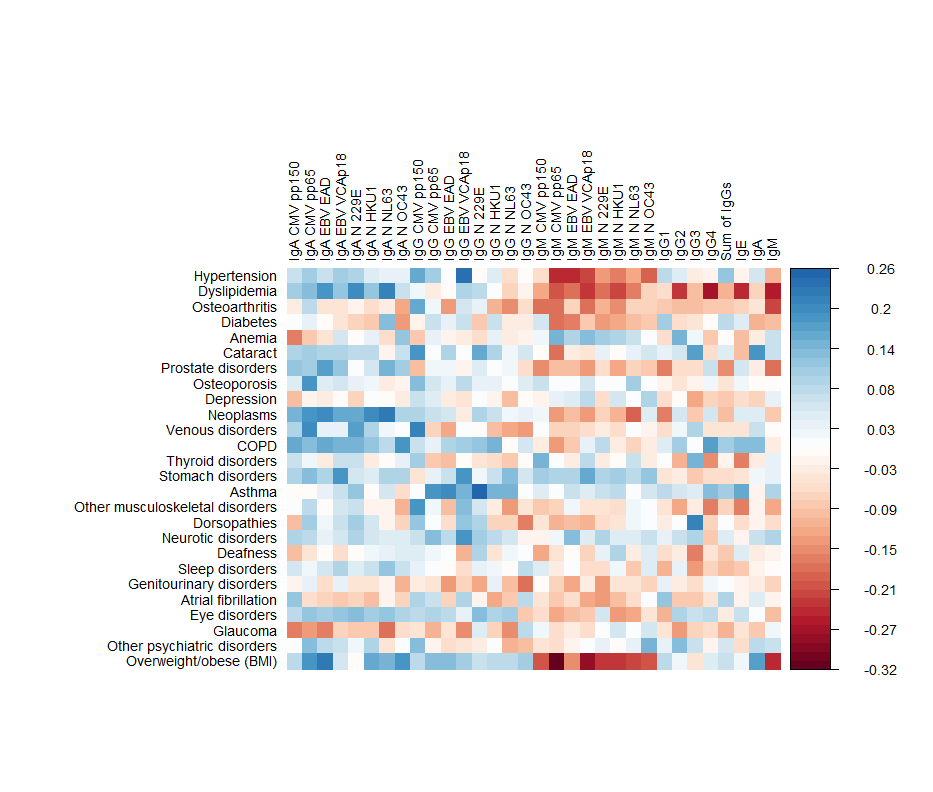


Spearman’s ρ (rho)

**Supplemental Figure 5**. Concentrations of IL-8, IL-2R, IL-6 and TNF-α in 2020-21 in the 20 participants who developed COVID-19 (panel a); and relative intraindividual change of IL-8, IL-2R, IL-6 and TNF-α concentrations in 2020-21 with respect to concentrations in 2016-17 in the 20 participants who developed COVID-19 (panel b). Vertical axis (panel a): Concentrations in 2020-21 (pg/mL). Vertical axis (panel b): Relative intraindividual change (%). Horizontal axis: Time interval from COVID-19 symptom onset to blood extraction (months).


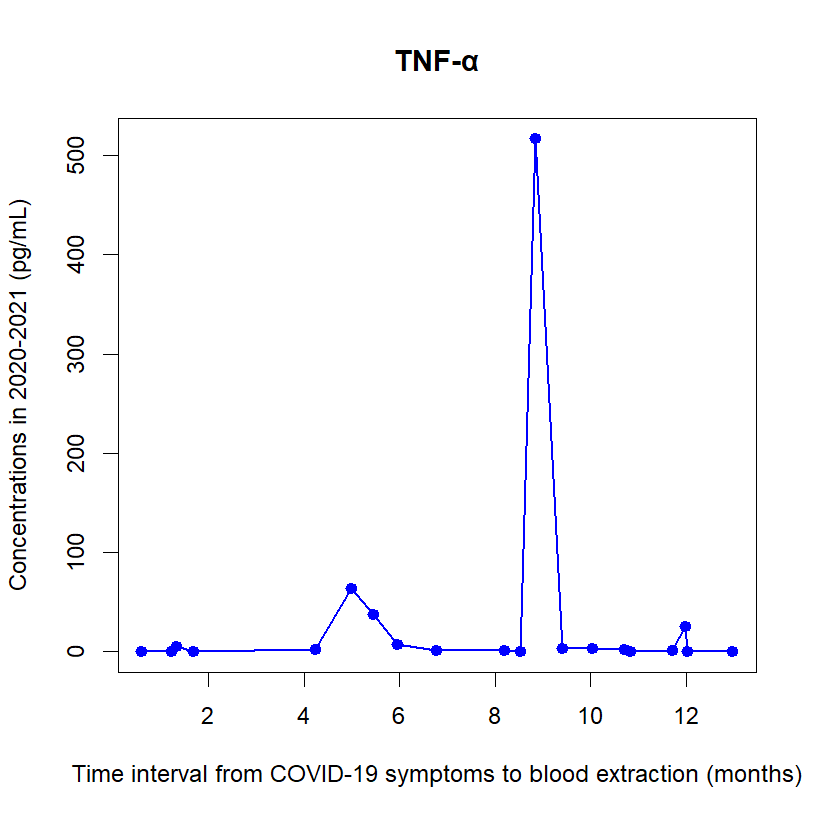

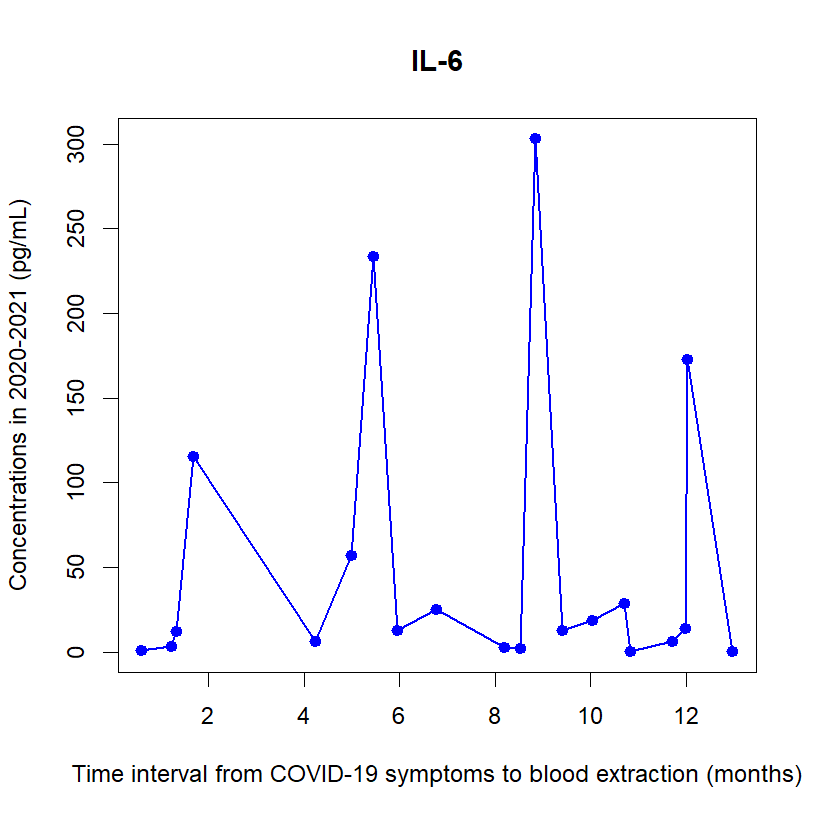

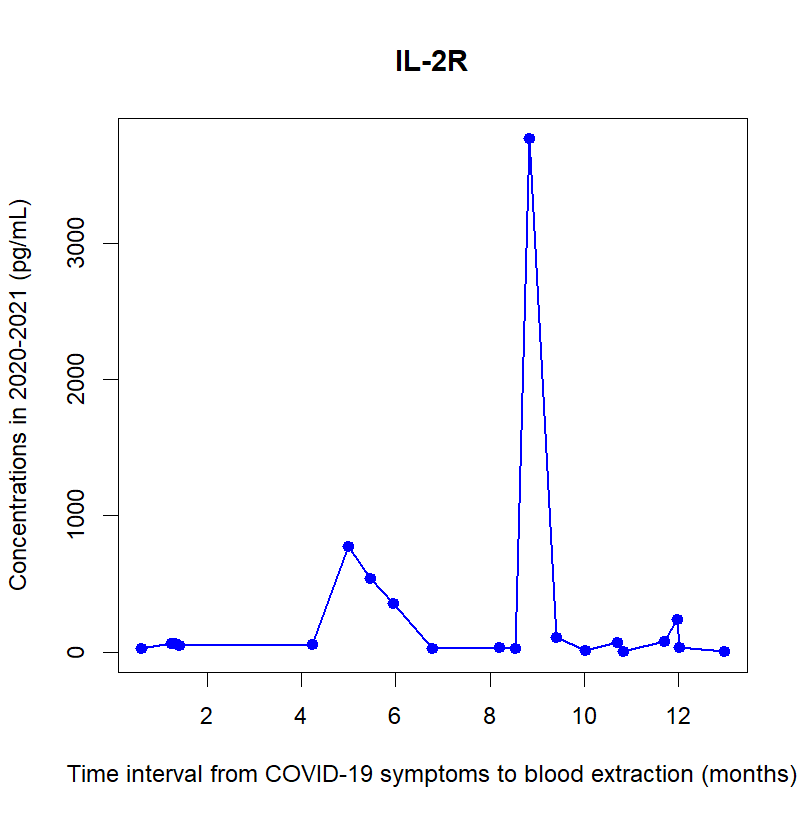

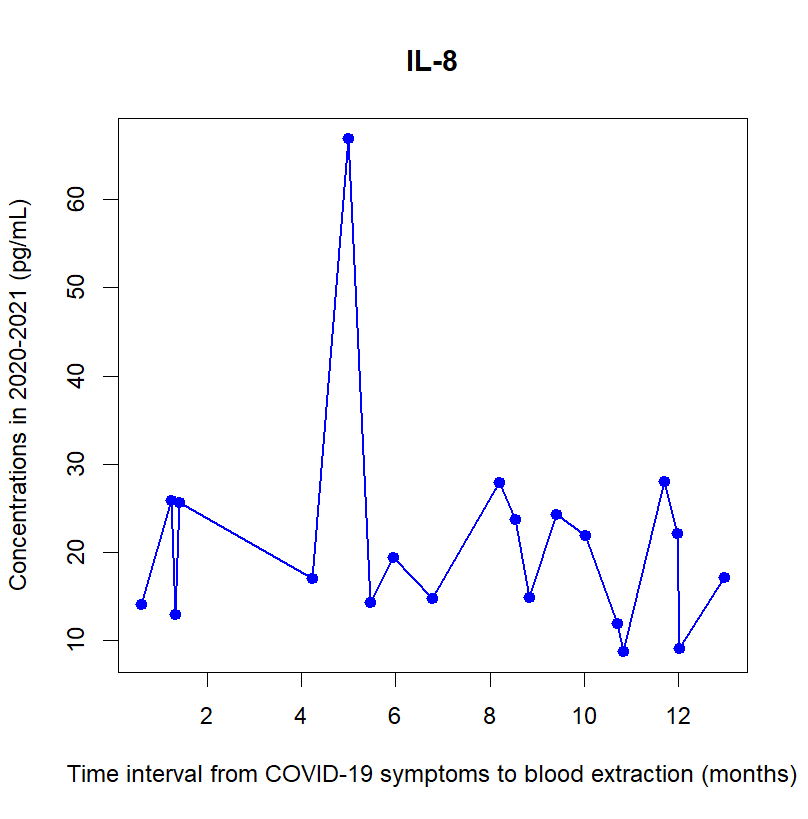


**a)**


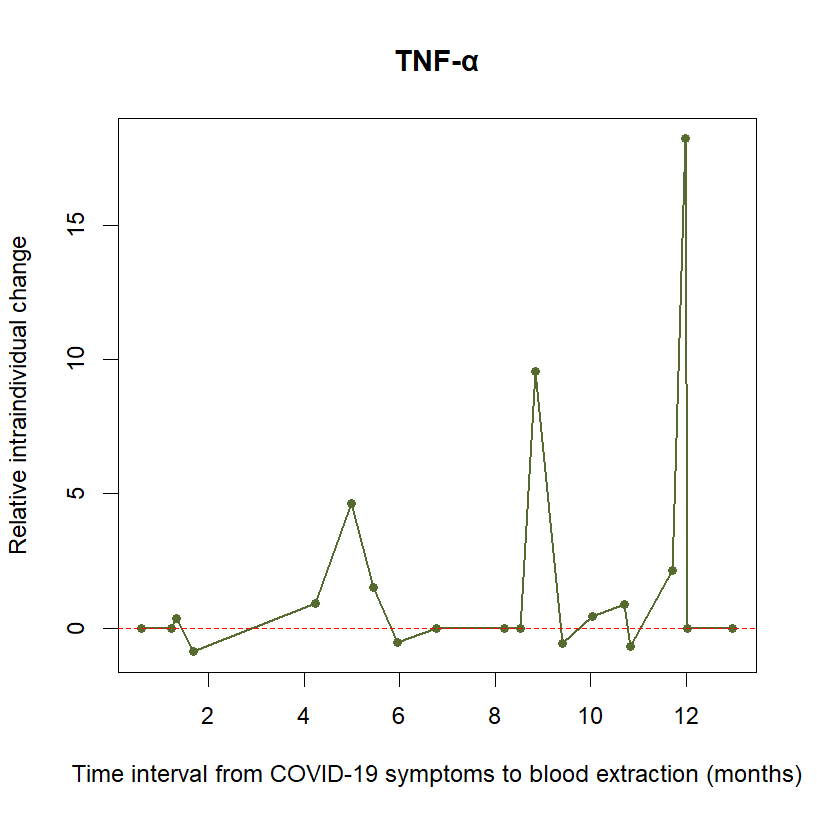

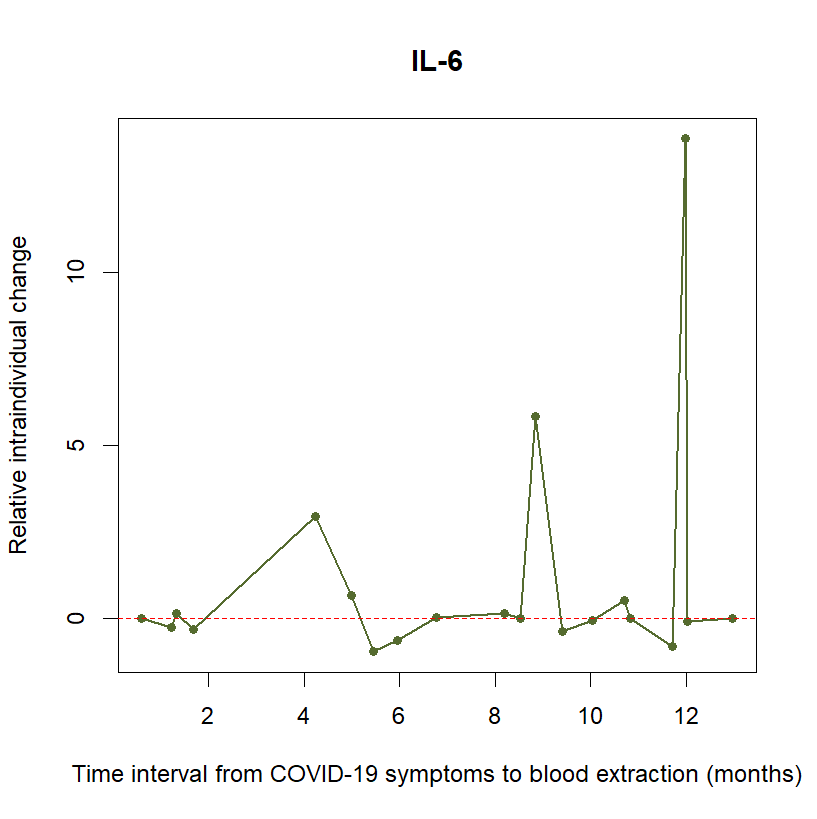


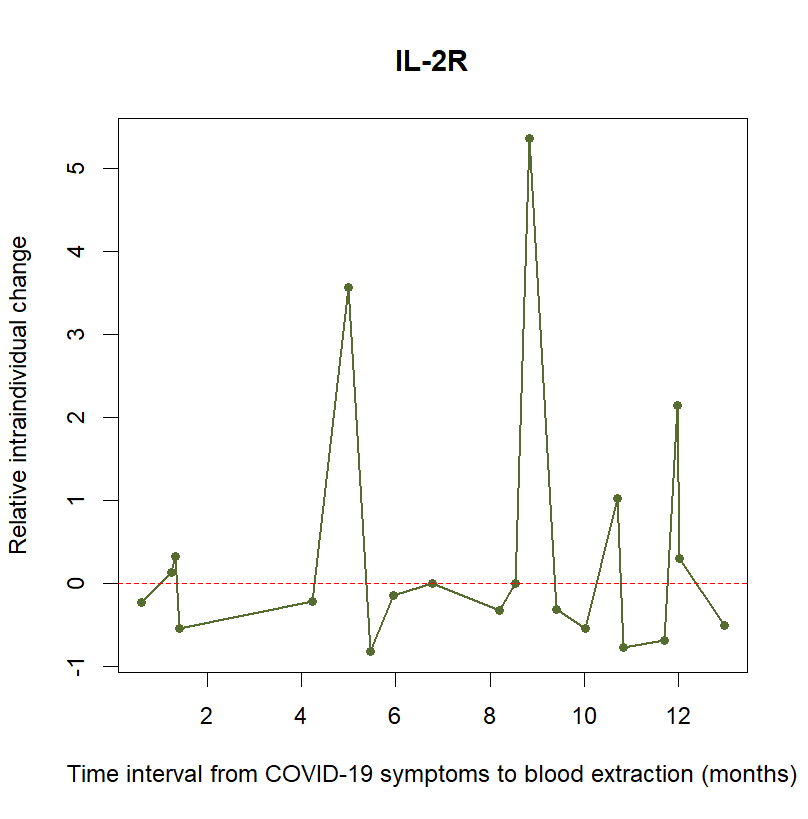

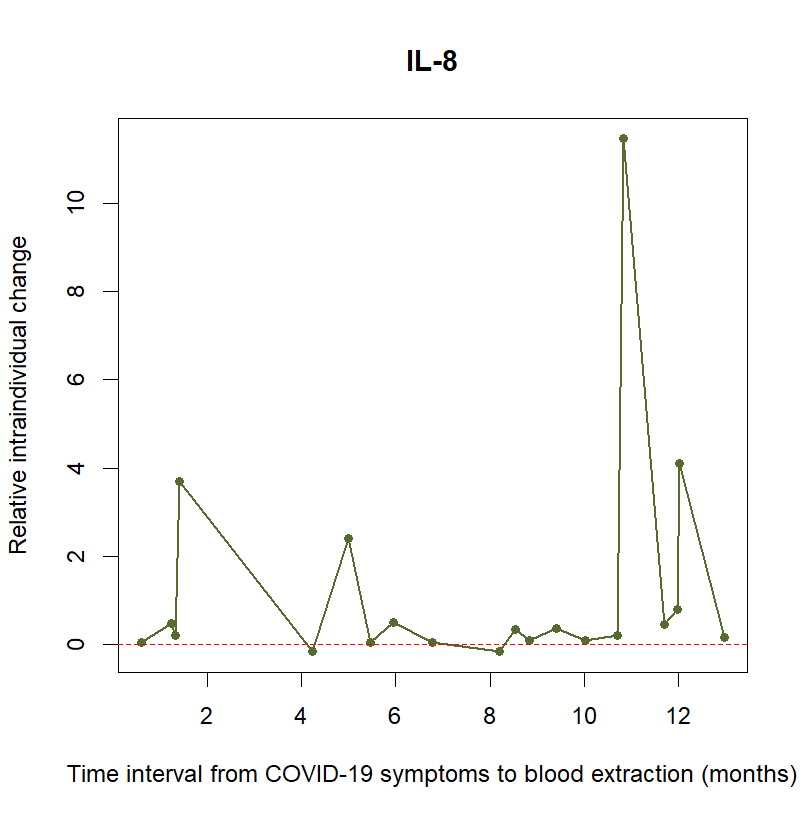
**b)**
